# The Roadmap of 2D Materials and Devices Toward Chips

**DOI:** 10.1007/s40820-023-01273-5

**Published:** 2024-02-16

**Authors:** Anhan Liu, Xiaowei Zhang, Ziyu Liu, Yuning Li, Xueyang Peng, Xin Li, Yue Qin, Chen Hu, Yanqing Qiu, Han Jiang, Yang Wang, Yifan Li, Jun Tang, Jun Liu, Hao Guo, Tao Deng, Songang Peng, He Tian, Tian-Ling Ren

**Affiliations:** 1 School of Integrated Circuits and Beijing National Research Center for Information Science and Technology (BNRist), Tsinghua University, Beijing, 100049 People’s Republic of China; 2 School of Microelectronics, Fudan University, Shanghai, 200433 People’s Republic of China; 3 State Key Laboratory of Dynamic Measurement Technology, Shanxi Province Key Laboratory of Quantum Sensing and Precision Measurement, North University of China, Taiyuan, 030051 People’s Republic of China; 4 High-Frequency High-Voltage Device and Integrated Circuits R&D Center, Institute of Microelectronics, Chinese Academy of Sciences, Beijing, 100029 People’s Republic of China; 5 School of Electronic and Information Engineering, Beijing Jiaotong University, Beijing, 100044 People’s Republic of China; 6 School of Integrated Circuits, University of Chinese Academy of Sciences, Beijing, 100049 People’s Republic of China; 7 IMECAS-HKUST-Joint Laboratory of Microelectronics, Beijing, 100029 People’s Republic of China

**Keywords:** Two-dimensional materials, Roadmap, Integrated circuits, Post-Moore era

## Abstract

Due to the constraints imposed by physical effects and performance degradation, silicon-based chip technology is facing certain limitations in sustaining the advancement of Moore’s law. Two-dimensional (2D) materials have emerged as highly promising candidates for the post-Moore era, offering significant potential in domains such as integrated circuits and next-generation computing. Here, in this review, the progress of 2D semiconductors in process engineering and various electronic applications are summarized. A careful introduction of material synthesis, transistor engineering focused on device configuration, dielectric engineering, contact engineering, and material integration are given first. Then 2D transistors for certain electronic applications including digital and analog circuits, heterogeneous integration chips, and sensing circuits are discussed. Moreover, several promising applications (artificial intelligence chips and quantum chips) based on specific mechanism devices are introduced. Finally, the challenges for 2D materials encountered in achieving circuit-level or system-level applications are analyzed, and potential development pathways or roadmaps are further speculated and outlooked.

## Highlights

- This review introduces the potential of 2D electronics for post-Moore era and discusses their current progress in digital circuits, analog circuits, heterogeneous integration, sensing circuits, artificial intelligence chips, and quantum chips in sequence.
- A comprehensive analysis of the current trends and challenges encountered in the development of 2D materials is summarized.
- An in-depth roadmap outlining the future development of 2D electronics is presented, and the most accessible and promising avenues for 2D electronics are suggested.

## Graphical abstract

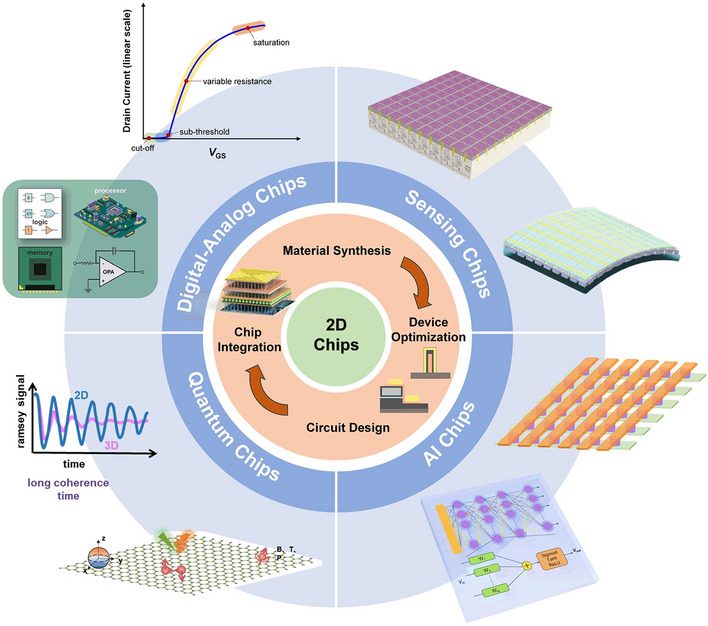

## Introduction

Since the advent of integrated circuits (ICs) in 1958, their technological advancements have been driven by the relentless progression of Moore's Law [1]. The development of silicon-based complementary metal–oxide–semiconductor (CMOS) technology, from the inception of the first CPU, Intel 4004 to the advent of FinFET-based circuits, has played a pivotal role in driving the advancement of the semiconductor industry and information technology. However, as transistor dimensions have reached the nanoscale, various physical phenomena, such as thickness-fluctuation-induced scattering [2], quantum tunneling, and other short-channel effects (SCEs) [3], have emerged in silicon-based devices, causing severe performance degradation and posing challenges to the continuous advancement of Moore's law. The conventional CMOS technology, which relies on size reduction for enhanced integration density, is now facing significant obstacles, but the demand for more powerful computing systems remains driven by rapid advances in applications like high-performance computing, Internet of Things (IoT), and artificial intelligence.

To foster further progress in microelectronics, three development directions have been proposed: More Moore, More than Moore, and Beyond Moore [4, 5]. The More Moore path seeks to persistently scale down transistor dimensions while mitigating the detrimental effects of short-channel phenomena through innovative approaches such as novel device structures or alternative materials. The More than Moore path emphasizes the optimization of circuit design, system algorithms, and advanced packaging techniques to improve chip performance. Furthermore, it aims to diversify the functionalities of electronic systems, encompassing fields such as sensing [6], RF applications [7], flexible electronics, bioelectronics [8], and the IoT [9]. The Beyond Moore direction, meanwhile, explores novel switch devices beyond traditional CMOS technology, including ferroelectric transistors [10], neuromorphic devices [11], and quantum devices [12], to realize advanced information processing capabilities.

To surmount the limitations of silicon materials and advance the development of post-Moore era, there is a need to explore novel semiconductor materials that can sustain stable and exceptional electrical properties even at reduced dimensions. 2D materials, characterized by their atomic-scale thickness, have garnered significant attention. Remarkably, 2D materials exhibit noteworthy carrier mobility and gate control performance even at the ultimate limits of miniaturization [13]. As a result, they have emerged as promising candidates for serving as channel materials in next-generation semiconductor devices within the More Moore domain. Furthermore, 2D materials demonstrate remarkable heterogeneity and compatibility with silicon and other 2D materials [14], allowing for seamless integration in advanced electronic systems. These materials also offer remarkable surface-to-volume ratios and surface activities, rendering them highly suitable for sensing applications within the More than Moore domain [15]. Moreover, their unique quantum properties and phenomena hold considerable potential for breakthroughs in the exploration of quantum devices. In summary, 2D materials hold substantial promise for advancing Moore’s law within the More Moore, More Than Moore, and Beyond Moore directions. They offer exceptional opportunities to overcome the current limitations and push the boundaries of microelectronics technology.

**Fig. 1:**
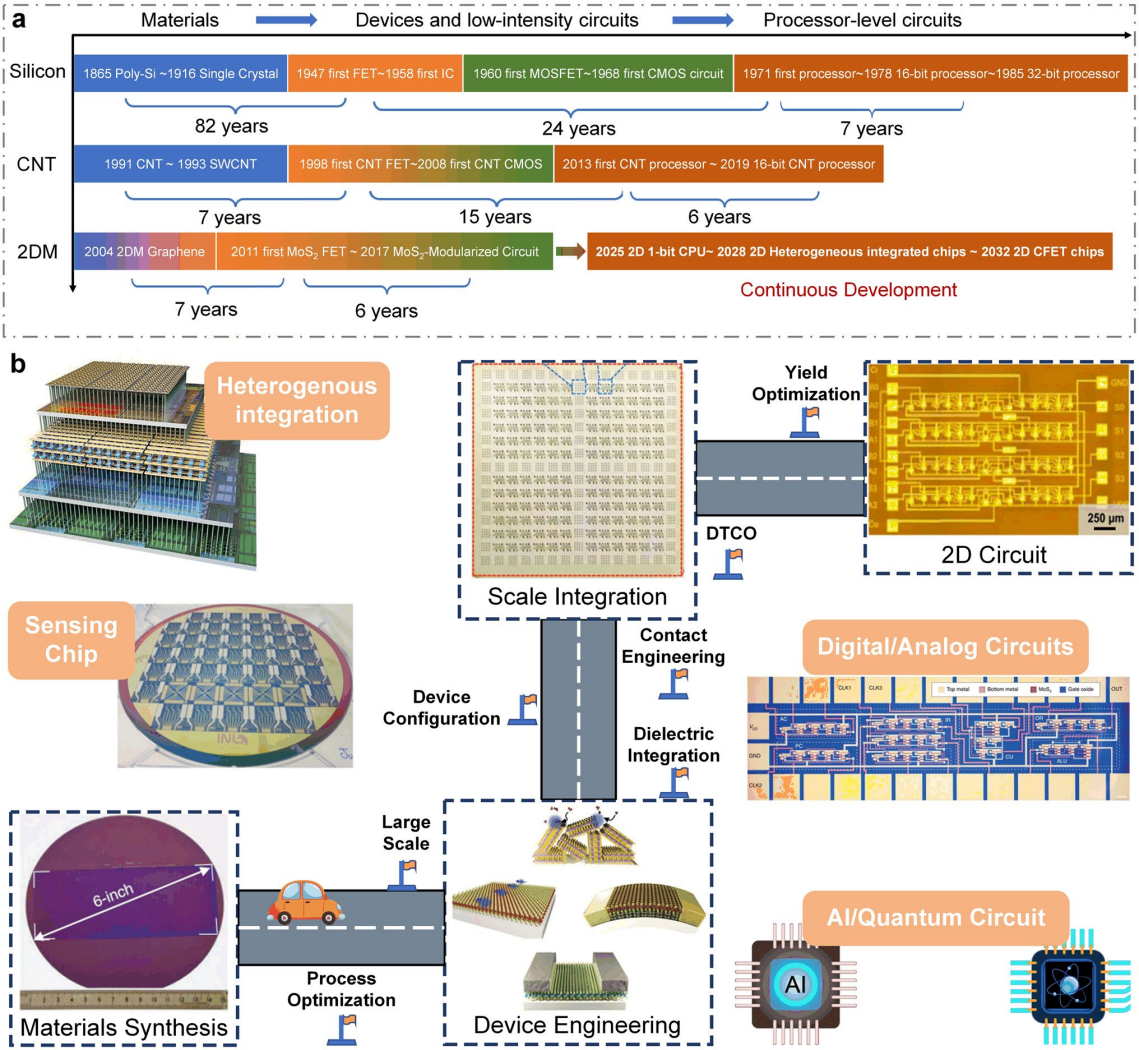
A schematic diagram of the general roadmap for 2D circuits. **a** Development timeline of silicon-based, carbon nanotube-based, and 2D ICs. **b** Route for the realization of 2D circuits and possible applications in the future. Reproduced with permission [17–20]. Copyright (2017), (2018), (2021), (2022), Springer Nature. Reproduced with permission [21]. Copyright (2023), American Chemical Society. Reproduced with permission [22]. Copyright (2017), Elsevier

At present, 2D materials encounter numerous opportunities and uncertainties. The development of mature silicon-based technology and another emerging material, carbon nanotubes (CNTs), could serve as valuable guidance and references for the advancement of 2D materials (Fig. 1a). However, the progression of 2D technology experiences a certain degree of delay due to factors such as limitations in material growth. It is still a challenge to achieve circuits beyond the processor level. More expectations are being placed on the substitution of silicon in specific domains. According to IMEC’s projection, it is anticipated that by 2032, 2D materials will genuinely find application in back-end-of-the-line (BEOL) processes [16]. Additionally, the development of 2D materials exhibits variations across diverse application domains. To further search for the most feasible pathway toward the realization of 2D materials’ application, the proposal of a roadmap becomes essential to provide assistance and insight.

In this review, we first present an overview of the characteristics and current development status of 2D materials. Subsequently, we discuss the advancements in 2D transistor fabrication processes including material synthesis, transistor engineering, material transfer and integration, and 2D package. Then, the recent progress and applications of 2D materials in typical fields are summarized, respectively (logic chips and heterogeneous integration chips in the More Moore domain, sensor chips in the More Than Moore domain, artificial intelligence (AI) chips, and quantum chips in the Beyond Moore domain). Finally, we address some of the challenges and issues that persist in the application of 2D materials, along with highlighting the potential directions where early applications of 2D materials are most likely to be realized.

## Promises of 2D Materials Toward Chips

**Fig. 2:**
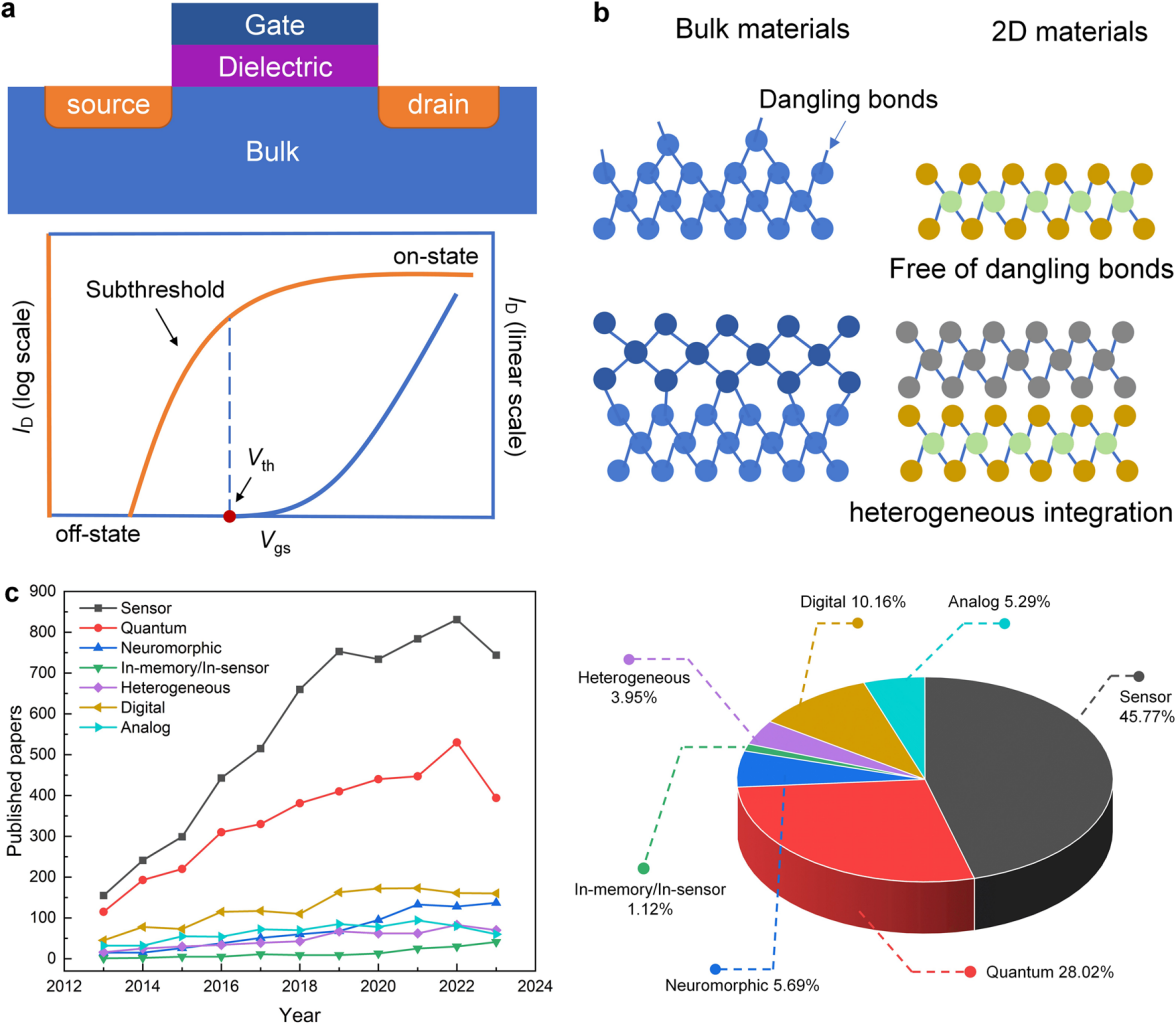
**a** Schematic of a traditional FET and typical transfer characteristic of FET device. **b** Partial advantages of 2D materials over bulk materials. **c** Number of 2D material publications from 2013 to 2023. (Keywords used for searching “two dimensional materials”, “wafer-scale OR circuit OR array” and the corresponding abbreviation for specific applications like “digital”, several typical 2D materials like MoS_2_ are also listed as keywords. Source: Web of Science Core Collection, accessed on January 3, 2024)

As the most important electronic device in modern ICs, the continuous advancement of the performance of field effect transistors (FETs) has led to rapid progress in electronic technologies. To accurately assess the digital and analog performance of FETs, a set of key device parameters are defined. The fundamental basis for digital logic applications is the ability to switch transistors on and off (Fig. 2a). Minimizing the off-state current (*I*_off_) is essential to reduce static power consumption. Achieving a large current ratio between the ON and OFF states (*I*_on_/*I*_off_) exceeding 10^5^ is imperative for enhanced control accuracy. The subthreshold swing (SS) determines the switching speed of FETs. When it comes to analog applications, the intrinsic gain (A_v_) is one of the most important analog parameters that determines the amplification capability of a single device. However, as the technology node goes down, all these device parameters degrade severely due to the second-order effects (e.g., SCEs) in Si-based FETs, limiting its further development [23]. The ideal semiconductor for sub-10-nm node should include the following features: (1) proper bandgap and excellent carrier transport properties with decreasing body thickness; (2) low defect density and good long-term stability; (3) producible in large scale, easy to integrate and compatible with Si CMOS technology [24].

The introduction of 2D materials as the channel is an attractive strategy to address the challenges associated with scaling down. Since the first 2D materials to be discovered, graphene was isolated in 2004 [25], a broad library of 2D materials has been explored broadly including transition metal dichalcogenides (TMDCs), black phosphorus (BP), hexagonal boron nitride (h-BN), MXene and so on [26]. Different from conventional bulk silicon materials, 2D materials exhibit lattice periodicity in the plane, resulting in the formation of Brillouin zones. Within this region, electron energy levels and wave functions are subject to stringent quantum constraints, allowing only specific energy levels to exist and giving rise to a quantized energy band structure. This quantum confinement effect gives 2D materials adjustable energy gaps, carrier confinement, and quantum tunneling effects. By controlling the geometry number of layers, heterostructures, etc., of 2D materials, or applying external strains, and electric fields to them, the lattice periodicity is changed, ultimately influencing the band structure and the size of the band gap. Furthermore, electrons are confined within the ultrathin body that allows excellent gate electrostatic control and has the potential of being immune to the SCEs. The mobility of 2D FETs has been enhanced to 350,000 cm^2^ V^−1^ s^−1^ based on graphene prepared by chemical vapor deposition (CVD) which is accessible for wafer-scale production [27]. In comparison, when the body thickness of silicon is below 5 nm, the fluctuation in material surface thickness and the pronounced scattering caused by surface dangling bonds significantly disrupt the structural periodicity of silicon [28]. The buckled structure of silicene breads the atomic plane’s symmetry, unlike graphene, resulting in particularly strong Zero-Acoustic (ZA) phonon scattering [29], leading to severe mobility degradation. Furthermore, due to the surface sensitivity of silicene derived from its mixed *sp*^2^–*sp*^3^ character [30], its instability in air necessitates specific preparation and usage conditions for its realization [31].

While graphene is not suitable as an active semiconductor material for electronic switching owing to its gapless band structure and graphene FETs show a limited *I*_on_/*I*_off_ ratio leading to high power consumption in standby mode [23]. Hence, graphene is usually used as metallic contact or gate materials instead of channel materials [32]. In addition to graphene, MoS_2_, WS_2_, and WSe_2_ from the family of TMDCs are developed to fabricate FETs due to their considerable bandgap (> 0.5 eV) [4]. The MoS_2_ FET with a 1-nm-wide gate electrode shows a nearly ideal SS of ~ 65 mV/decade and a large *I*_on_/*I*_off_ ratio of ~ 10^6^ [33]. The h-BN is the most common 2D insulator (*E*_g_ ≈ 6 eV) and is usually considered to be the most promising gate insulator in 2D-based FETs because of the ideal interface without scattering. Efforts have also been invested in the development of novel 2D dielectric materials, like CaF_2_ [34]. The diverse lattice structures and atomic arrangements of 2D materials give rise to distinct electronic band structures, resulting in a wide energy band range, covering semimetals, semiconductors, and insulators. Besides, with the dangling-bond-free surface, the carrier mobility of 2D transistors varies little with body thickness and it is easy for 2D materials to integrate with other kinds of materials without being constrained by lattice constant matching [35].

Furthermore, with the advent of the big data era, several novel computing architectures and mechanisms have been introduced for next-generation computing technologies. Benefiting from the fascinating characteristics of 2D materials, 2D device innovation has become the focus of intense research such as 2D memory devices, neuromorphic devices, and quantum-engineering devices. 2D memristor, memtransistor, flash memory, and ionic transistor are widely exploited for in-memory computing or artificial intelligence technology [11]. 2D materials can not only extend Moore’s Law to sub-5-nm nodes but also boost memory performance. For example, owing to the ultrathin body of 2D materials, 2D memory devices can use a low working voltage and reduce power consumption. Semi-metallic graphene was reported to be suitable as a contact material for electrodes presenting ultralow switching power or a blocking layer to inhibit efficient carrier injection due to its low density of states [36]. The h-BN could be applied as the switching layer for memristive memory devices with high endurance and long retention time [37]. What’s more, continuous discovery and research on novel physical mechanisms for applications in low-power switches have been proposed and investigated including tunneling transistors [38], ferroelectric transistors [39], and filament-based FET [40]. Those emerging FET devices have great potential to lower the SS and subsequently lower the supply voltage and energy consumption. S. Kim et al. reported a natural heterojunction-tunneling FET with a spatially varying layer thickness in BP without interface problems. And the device’s SS has achieved a record-low level (SS_ave_4dec_ ~ 22.9 mV dec^−1^) [38]. The unique quantum properties of 2D materials also have been developed. They have strong spin–orbit coupling strength leading to enhanced Rashba effect and easier modulation of the electron spin through an externally applied electric field. The atomic-scale thickness of 2D van der Waals (vdW) heterostructure can be utilized to maximize the spin-injection efficiencies [12]. And there’s plenty of application room for the computing capability drive after Moore’s law to 2D materials.

## Process Engineering for 2D Chips

Lots of efforts have been devoted to the exploration of 2D materials’ physical characteristics or device optimization. Most of the early demonstrations of 2D FETs are based on mechanically exfoliated flakes. And the research has been confined to individuals or a limited number of devices. However, to achieve circuit-level applications of 2D materials, the scale of devices should be expanded to the wafer-scale, which poses higher requirements on material synthesis, device processing, and integration technology. In this section, we will introduce and discuss the research progress in the field of process engineering for 2D chips.

### Material Synthesis

Device performance consistency is a crucial foundation for achieving 2D circuits, and device performance fluctuations need to be confined within a certain range [41]. In terms of material quality, the realization of chip-level 2D circuits requires the synthesis of wafer-scale single-crystal (WSSC) materials that are uniform and have low defect densities. Due to constraints related to material size, controllability, and repeatability in the fabrication process, traditional mechanical exfoliation methods are unsuitable for wafer-scale preparation and industrialization. In this section, we will introduce the preparation methods for wafer-scale 2D materials from various perspectives, including CVD, metal–organic chemical vapor deposition (MOCVD), mechanical exfoliation, and liquid-phase exfoliation, with a particular focus on the wafer-scale preparation of TMDCs materials.

#### Chemical Vapor Deposition

**Fig. 3:**
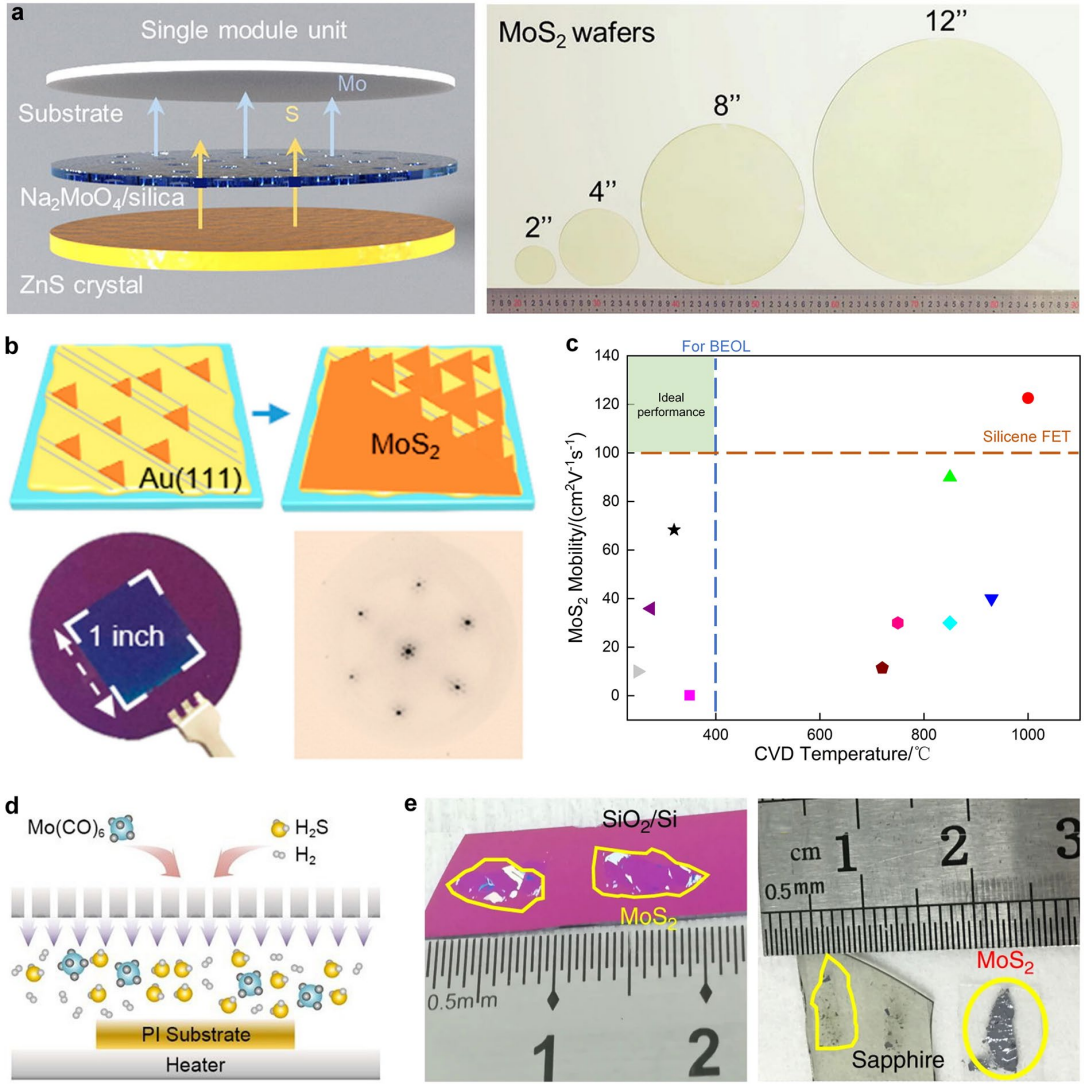
**a** Schematic of a single 2-inch producing module unit and photograph of uniform MoS_2_ film with wafer sizes ranging from 2-, 4-, 8- to 12-inch. Reproduced with permission [42]. Copyright (2023), Science China Press. **b** Schematic illustration of the MoS_2_ growth process on Au (111) substrate and representative μ-LEED pattern and photograph of a 1-inch uniform single-crystal MoS_2_ monolayer. Reproduced with permission [43]. Copyright (2020), American Chemical Society. **c** The relation between CVD temperature and MoS_2_ mobility in several reports [19, 44–52]. **d** Schematic illustration of the MOCVD process. Reproduced with permission [44]. Copyright (2021), Wiley–VCH. **e** Optical images of exfoliated MoS_2_ on SiO_2_/Si, sapphire, and plastic film. Reproduced with permission [53]. Copyright (2020), Springer Nature

The CVD method is currently one of the most promising approaches for the direct synthesis of large-area monolayer 2D materials [41]. Its advantages of low cost and scalability have sparked widespread research interest in the field of 2D materials. It has been reported that the uniform MoS_2_ wafer size could be extended to a 12-inch diameter which is crucial for the transition of TMDC materials from laboratory research to integrated components in next-generation semiconductor devices [42] (Fig. 3a). Due to the sensitivity of material growth, the substrate imperfection (e.g., grain boundaries) may lead to vacancies, impurities, wrinkles, and thickness fluctuations in the 2D sheet and the growth of polycrystalline 2D materials, which would threaten the yield and stability of devices or even result in circuit failure.

Orientation-controlled growth is considered an effective approach for achieving the preparation of WSSC TMDCs materials. By selecting a suitable substrate that matches the lattice of TMDCs, it is possible to grow TMDC films with high crystallinity, complete lattice structure, and controllable lattice orientation [54]. Substrates with specific crystal orientations, such as Au(111) and sapphire (0001), have been successfully employed for the preparation of WSSC TMDCs [43, 55] (Fig. 3b). L. Liu et al. reported the uniform nucleation of bilayer MoS_2_ on c-plane sapphire. Without the transfer step, the fabricated short-channel FET devices based on bilayer MoS_2_ exhibit an on-state current of 1.27 mA μm^−1^ [46].

While there is another challenge that must be noted——the thermal budget. The current CVD growth methods cannot avoid the use of high temperature to achieve good crystalline, while allowable thermal budgets for the BEOL technologies are lower than 400 ℃ [56]. The low growth temperature would lead to the formation of grain boundaries and stoichiometric defects in MoS_2_ which is detrimental to lateral transport [68]. The trade-off between CVD temperature and MoS_2_ mobility determines the potential widespread adoption of CVD-based MoS_2_ applications in the future (Fig. 3c).

#### Metal–Organic Chemical Vapor Deposition

Different from the CVD method, MOCVD employs metal–organic compounds as precursors and achieves uniform supply throughout the entire substrate (Fig. 3d). And most 2D TMDC materials could be synthesized without the limitation of substrate constraints benefited from the strong driving force generated through pyrolysis. This enables MOCVD to achieve arbitrary combinations of various thin films and substrates [57]. Additionally, MOCVD can adjust the reactant concentration by controlling the reactant partial pressures, thereby obtaining thin films with uniform electrical properties. Therefore, compared to the CVD method, the MOCVD method is more suitable for the production of high-quality, large-area 2D thin-film materials. Kang et al. reported the fabrication of 4-inch WSSC TMDC materials with high mobility (up to 30 cm^2^ V^−1^ s^−1^ at room temperature) using the MOCVD method [58]. Furthermore, MOCVD exhibits excellent process compatibility, enabling heterogeneous integration growth with LEDs while allowing for direct epitaxial growth at lower temperatures, making it highly compatible with CMOS technology [59]. Recently, scientists from MIT have proposed a low-thermal-budget synthesis method that enables the low-temperature rapid growth (< 300 ℃, growth time < 60 min) of single-layer MoS_2_ with a size of up to 200 mm and a high carrier mobility of 35.9 cm^2^ V^−1^ s^−1^. This breakthrough was achieved by separating the low-temperature growth zone from the high-temperature sulfur precursor decomposition zone [46]. However, the toxicity of commonly used precursors in MOCVD, like Mo(CO)_6_, H_2_S, is one of the main limiting factors for their widespread adoption, and further exploration is still required [60].

#### Mechanical Exfoliation Method

The mechanical exfoliation method is a technique that exploits the layered characteristics of 2D materials to achieve interlayer mechanical separation. The resulting 2D materials can retain a high degree of crystal structure and crystallinity, making them suitable for the preparation of small-area, high-quality single-crystal materials. Additionally, the operation and required equipment for this method are relatively simple, making it an important approach in current research for exploring novel structures or properties of 2D devices. To further increase the size of exfoliated 2D materials, enhancing the adhesion between the crystal source and substrate is a crucial prerequisite. Some noble metals, such as Au and Ni, have been proven to assist in achieving the large-area exfoliation of 2D materials [53, 61]. Y. Huang et al. introduced a one-step, and universal Au-assisted mechanical exfoliation method and successfully isolated 40 types of single-crystalline monolayers, most of which are millimeter-size and high-quality [53] (Fig. 3e). Although the scale of exfoliated 2D materials is extended to a considerable size, the repeatability and uniformity of the improved exfoliated method are still not guaranteed. On the other hand, the size and crystallinity of the source crystals are also critical factors limiting the scalability of the process. To achieve industrial-scale manufacturing in the future, it will be necessary to develop improved exfoliation processes and techniques for the fabrication of wafer-level single-crystal source materials.

#### Other Methods

Currently, the methodologies employed for the synthesis of WSSC monolayer TMDCs remain in a nascent stage of development. Factors such as production cost and the stability of manufacturing processes pose significant challenges, necessitating further dedicated research and optimization efforts for their prospective industrial-scale production. It is worth emphasizing that the cost-effective fabrication of WSSC 2D materials remains a formidable undertaking. However, it is crucial to acknowledge that not all applications mandate the utilization of high-quality, large-area single-crystal materials. The exceptional flexibility, high surface area, and sensitivity intrinsic to 2D materials render them suitable not only for applications in logic chip manufacturing but also for the development of flexible devices, sensors, wearable technology, and more [62]. These applications exhibit relatively lower demands regarding material quality [63]. Several cost-effective and scalable methods have been proposed, including liquid-phase exfoliation [64], screen printing [65], and inkjet printing [63], among others. These approaches possess the capability of wafer-scale fabrication, and some printing techniques facilitate patterned design as well. They offer significant advantages in terms of batch production, exfoliation efficiency, and product dimensions.

In summary, the fabrication of WSSC 2D materials remains a significant challenge in the current development of 2D chips. Researchers have been working on improving existing processes from various angles, including material growth mechanisms, source material selection, and process optimization, particularly in the case of CVD methods. Some progress has been made, but further enhancements are needed in terms of stability, scalability, and manufacturing cost. Additionally, unconventional growth strategies, such as geometric confinement [66] and defect engineering [16], have been proposed to address the limitations of material growth. Moreover, for applications that have relatively lower material quality requirements, some unconventional yet economically viable methods for 2D material fabrication, like printing techniques and liquid-phase exfoliation, hold promise for driving the industrialization of 2D materials [67]. Therefore, it is essential to continue refining the fabrication techniques for WSSC 2D materials while promoting the development of high-performance, cost-effective, and versatile devices based on 2D materials.

### FET Engineering Approaching the Theoretical Limit

The urgent requirement for the application of 2D materials into chips boosts the research enthusiasm and development of FET engineering. Many studies have endeavored to exploit the unique structure of 2D materials for the development of novel miniaturized transistor devices, aiming to achieve the ultimate scaling of gate length or channel dimensions. Simultaneously, device performance optimization has been pursued through the optimization of dielectric layer integration and electrode integration techniques. In this part, we will elucidate the approaches for the ultimate optimization of 2D materials from three perspectives: device structure, dielectric layer integration, and contact engineering.

#### Device Configuration

**Fig. 4:**
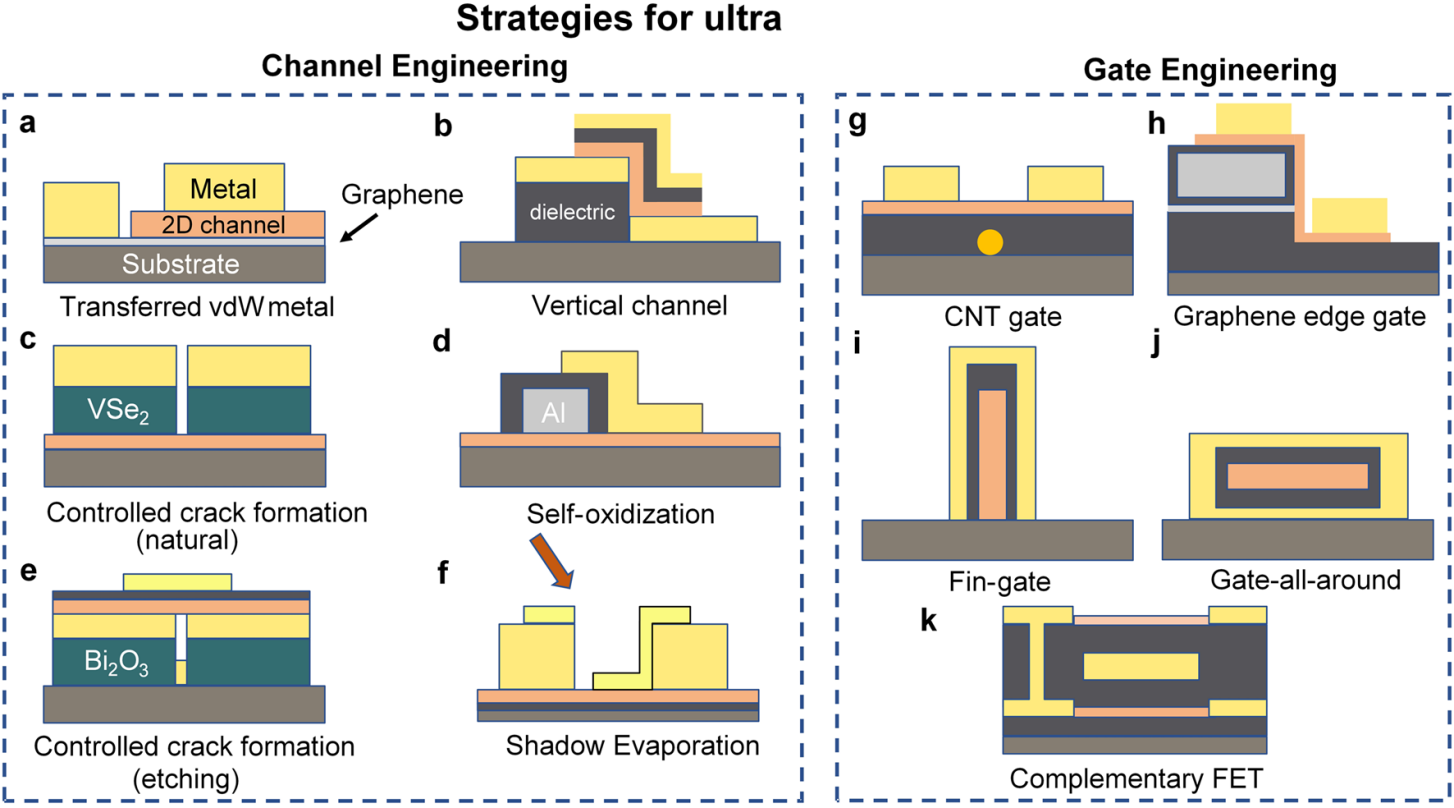
Typical strategies for ultra-scale transistors. Channel engineering: **a** Sub-1-nm MoS_2_ vertical transistors fabricated by transferred vdW metal electrodes [75]. **b** Ultrashort vertical-channel vdW semiconductor transistors [74].** c** Channel defined by controlled crack formation (natural) [72]. **d** 10 nm short channel transistor fabricated by self-oxidization of aluminum [70]. **e** Channel defined by controlled crack formation (etching) [73]. **f** Ultrashort MoS_2_ transistors fabricated by shadow evaporation [71]. Gate engineering: **g** buried CNT gate with a diameter of 1nm [33]. **h** Sidewall graphene edge gate with sub-1-nm thickness [69]. **i,j** 2D FinFET and GAAFET [77, 78]. **k** Complementary FET comprising p-type WSe_2_ FET and n-type MoS_2_ FET [79]

Currently, the typical structures of 2D FETs are top-gated and bottom-gated 2D FETs, which have been widely used for research on the basic properties of layered materials [13, 68]. And bottom-gated 2D FETs can be further divided into global back-gated FETs and buried-gated FETs. Based on these single-gate structures, efforts have been devoted to the size scaling process. In terms of scaling *L*_g_ (length of the gate electrode), Desai et al. demonstrated MoS_2_ transistors with a 1-nm physical gate length using 1D single-wall carbon nanotube (CNT) as the gate electrode [33]. Moreover, a recent report about the application of 2D single-layer graphene has scaled the L_g_ down to the physical limit. The 0.34 nm thickness of graphene can be regarded as the shortest *L*_g_, which approaches the physical limit among the known materials [69]. When it comes to the scaling down of *L*_ch_ (length of the channel layer), diverse processing methods have been developed to decrease the *L*_ch_ such as aluminum self-oxidization technology [70], shadow evaporation method [71], crack formation [72, 73], h-BN spacer [74], and vdW metal electrodes transfer method (Fig. 4) [75]. R. Wu et al. demonstrated sub-100-nm-L_ch_ bilayer tungsten diselenide (WSe_2_) transistors using vdW epitaxy and controlled crack formation processes. The 20-nm-long WSe_2_ transistor shows comparable performance to silicon transistors under similar channel lengths and driving voltages [72]. L. Liu et al. fabricated the sub-1-nm MoS_2_ vertical transistors through the low-energy vdW metal integration technique. Similar to the above-mentioned graphene-gated transistors utilizing the intrinsic thickness of 2D materials, this report utilized the 0.65-nm-thick MoS_2_ as the vertical channel layer with I_on/off_ of ~ 26 [75]. The poor switching performance can be attributed to the SCEs and strong tunneling effect in the off-state.

To further enhance the gate-control performance of 2D transistor devices, several emerging structures applied in advanced process nodes such as double-gate FET (DGFET) [76], FinFET [77], gate-all-around FET (GAAFET) [78], and complementary FET (CFET) [79] have also been proposed in 2D technology against SCEs. Dorow et al. fabricated DGFETs by optimized atomic-layer-deposition (ALD) process for 2D material surfaces. A steep SS of 75 mV dec^−1^ and a low DIBL of 12 mV V^−1^ were achieved owing to the enhanced electrostatics from double gates and high-quality gate oxide layer [80]. In 2022, the first GAA monolayer MoS_2_ nanosheet FET was demonstrated by TSMC. And the performance is remarkable with 410 μA μm^−1^ *I*_D_ at 1V *V*_D_ at 40 nm gate length and nearly zero DIBL that opens the path for stacked sheet GAA devices using 2D materials [78]. The clean surface of 2D materials makes it an ideal candidate for 3D stacking technology in the future. When it comes to 2D FinFET, the first demonstration was fabricated by the template-growth method, while the device performance was unsatisfactory with SS obtained to be 300 mV dec^−1^ and extracted mobilities to be in the range of 1 ~ 6 cm^2^ V^−1^ s^−1^ [81]. Recently, another 2D FinFET based on Bi_2_O_2_Se/Bi_2_SeO_5_ epitaxial heterostructures was reported. This FinFET exhibits exciting performance including high electron mobility up to 270 cm^2^ V^−1^ s^−1^, ultralow *I*_off_ down to ~ 1 pA μm^−1^, and high *I*_on_/*I*_off_ up to 10^8^ [77]. The inspiring results open up new avenues for the further extension of Moore’s law. However, it should be noted that most of these emerging structures used electron beam lithography (EBL) to pattern electrodes and channel layers, which are expensive and time-consuming. More consideration should be focused on its compatibility with Si-based processes and the feasibility of large-scale production. Simultaneously, the optimization of individual devices remains insufficient; the considerations of universality, process repeatability, and the potential for significant enhancement of integration density must also be taken into account.

#### Dielectric Integration

To achieve device size miniaturization, simultaneous progress is required in the development of complementary 2D dielectric systems in parallel with channel materials. Moreover, the atomic-level thickness channels are more sensitive to the interface with gate dielectric, which limits further improvement of device performance [82]. Therefore, researches about dielectric integration of 2D FETs are focused on two aspects: One is the search for low equivalent-oxide-thickness (EOT) dielectric materials, and the other one is the optimization of preparation methods and interface quality.

As the downsizing of transistor’s size, the decrease of gate oxide thickness is also one of the critical technologies. Unfortunately, the relatively low permittivity of SiO_2_ (ε_r_ ≈ 3.9) limits its application in FETs with gate lengths scaled down to sub-10 nm [83]. Thus, novel gate dielectrics with higher dielectric constants should be developed. After decades of search for the appropriate high-k materials, Hf-based oxides, such as HfO_2_ (κ ≈ 19), stand out as the first-generation CMOS products, which are also commonly used materials in 2D technologies [84]. Whereas, considering the continuous scaling requirements and possible damage to the channel materials from the deposition process, further efforts have been devoted into the development of 2D dielectrics. 2D vdW insulators, in which atomic layers are stacked via vdW interaction, are regarded as the ultimate solution for dielectric miniaturization. h-BN has been widely investigated in 2D transistors as a dielectric layer. The preparation of h-BN has achieved wafer-level and is used for device arrays [85]. However, the excessive tunnel currents through ultrathin h-BN limit its further application in nanoscale 2D devices [86]. Besides, several emerging 2D insulators, like CaF_2_, Bi_2_SeO_5_, and SrTiO_3_, are explored and exhibit excellent dielectric performance [34, 87, 88]. For example, Zhang et al. created top-gated 2D transistors with sub-0.5-EOT dielectrics, Bi_2_SeO_5_ (κ =  ~ 22), and exhibit leakage current below the low-power limit of 0.015 A cm^–2^ at 1 V gate voltage [89]. Furthermore, the proper range should be estimated in conventional Si MOSFET due to the effects of both drain-induced barrier lowering and fringing-induced barrier lowering (FIBL). However, benefiting from the vdW gap between 2D semiconductors and dielectrics, the unfavorable FIBL effect is mitigated [87].

To help improve 2D materials’ actual performance, the optimization of the preparation process and improvement of interface quality are another important issue. ALD is a common method to prepare a dielectric layer which has been applied in industrial production [90], while, due to the dependence of chemical reactions on surface dangling bonds and nucleation sites, corresponding requirements are put forward for the substrate materials. Considering the clean surface of 2D materials and the existence of organic residues during the transfer process, it is necessary to introduce the optimized ALD method to improve the uniformity and quality of dielectric layer growth [91].

Many interfacial activation layers and processes have been developed for the uniform deposition of high-κ oxides on 2D materials including chemical pretreatment of the surface and the introduction of a seeding layer. For example, Li et al. presented a technique to integrate ultrathin high-κ dielectric (EOT =  ~ 1nm) on 2D materials using a PTCDA molecular crystal as a seeding layer. The resulting PTCDA/HfO_2_ gate dielectric exhibits low leakage current and high breakdown field, meeting the ITRS requirement for low-power devices [92]. When selecting a seeding layer for depositing high-κ dielectrics on 2D channels, it is crucial to consider its potential impact on the properties of the channel materials. The ideal seeding layer should help form a uniform and conformal interface with the dielectric without sacrificing the performance of the channels. When it comes to the pretreatment method, functional groups and dangling bonds are introduced by using plasmas [93]. Although this method avoids the introduction of additional layers, the physical plasma damage would result in the degradation of device performance attributed to bond disruption and alignments [94]. In addition to ALD, several gentler integration methods are also developed to minimize the damage to 2D layers like native oxide method and the transfer of wafer-scale insulators [95]. Certainly, the issues that need to be considered are still the scalability of methods, the uniformity of material quality, and compatibility with current Si-based technologies.

#### Contact Engineering

As a bridge connecting 2D materials with a three-dimensional system, contacts in 2D devices are one of the key aspects to be considered when exploring the electronic properties of 2D materials. The difference in electronic energy levels and lattice structures between the electrode material and 2D material leads to electron transfer and the formation of new interface states, i.e., metal-induced gap states (MIGS). The MIGS results in the Fermi level of 2D materials being pinned to a specific position, known as the Fermi level pinning (FLP). In addition, impurities and interface defects introduced by electrode deposition can also lead to substantial contact resistance. These restrict electron flow and adjustment of contact potential, leading to hysteresis or current saturation in transistor devices, the inability to achieve bipolar transport, an increase in device leakage current, and slower switching speeds [99]. Therefore, achieving high-quality contacts in 2D devices is very important, which can help exhibit the expected excellent performance of 2D materials.

**Fig. 5:**
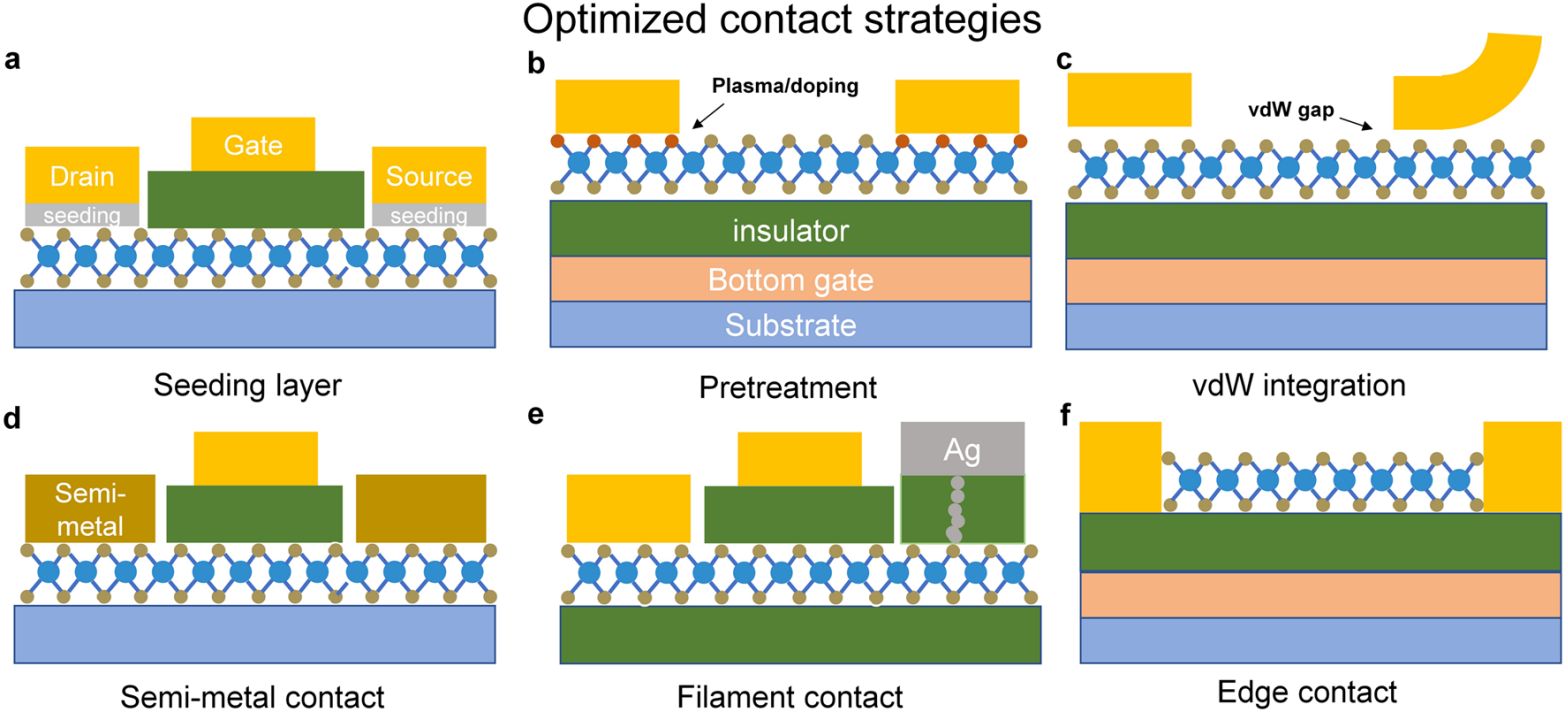
Typical optimized contact strategies for 2D transistors. **a** Introduction of a seeding layer for contact integration or improving contact quality [92]. **b** Doping of contact region or pretreatment of the interface [93]. **c** VdW integration process [96]. **d** Development of novel materials such as semi-metal contact [87, 97]. **e, f** Novel contact modes, filament contact, and edge contact [40, 98]

Currently, there are three main strategies for improving contacts: adopting novel contact materials, optimizing the interface structures, and developing new contact processes. Figure 5 summarizes some novel technologies for achieving high-quality contacts. And the development of novel contact materials is a highly active research direction for suppressing MIGS and lowering Schottky barriers in FETs. Among the various metals and semimetals, Scandium (Sc) [100], Bismuth (Bi) [97, 100], and Antimony (Sb) [101] have been successfully verified to effectively optimize the contact conditions. Specifically, Shen et al. demonstrated that the orbital resonance between Bi and MoS_2_, along with the near-zero density of states at Fermi level, enabled the fabrication of Bi-MoS_2_ FETs with reduced contact resistance reduced (123 Ω μm) and improved carrier concentration (1.5 × 10^13^ cm^–2^) [97]. Similarly, Li et al. reported that the band structure of Sb (0112) overlaps well with MoS_2_, which can be further enhanced by strong vdW forces. As a result, the use of Sb as a contact material in 2D FETs led to a significant reduction in contact resistance (42 Ω μm) and a high *I*_on_/*I*_off_ over 10^8^. Notably, these performances outperformed equivalent silicon CMOS technologies and met the 2028 roadmap target [101]. We could find that by selecting appropriate electrode materials, excellent electrical properties such as low contact resistance can be achieved. However, it is important to evaluate the stability and consider its applicability to other 2D materials. And it is crucial to take into account issues like cost and process compatibility, particularly for large-scale manufacturing.

Besides, interface modification is considered a viable strategy for mitigating contact resistance, with phase engineering gaining widespread attention due to its strong process compatibility. It has been experimentally observed that some 2D materials like MoS_2_, MoTe_2_, MoSe_2_, and other TMDCs demonstrate varying characteristics in different phases. Utilizing this theory, researchers have proposed phase engineering to reduce FLP and lower the Schottky barrier height (SBH). Various techniques have been proposed and investigated to induce phase transitions, including laser-induced phase patterning [102], electrostatic doping [103], argon (Ar) plasma processing [104], and so on. For instance, laser-induced phase patterning and Ar plasma handling achieve the transition of TMDCs from 2H to 1 T phase through the introduction of chalcogen vacancies. An atomically sharp interface and similar WF between 2H and 1 T phase result in a low contact resistance. Furthermore, these methods are compatible with standard lithography techniques, allowing for the formation of clean homojunctions and mitigating contamination by extraneous chemical components [102, 104]. It is noteworthy, however, that some chemical processing steps can introduce defects and impurities in TMDCs [105]. Additionally, it is essential to recognize that physical methods may not be readily adaptable to large-scale preparation. Moreover, in the pursuit of establishing more durable and dependable interfaces, research endeavors can be directed toward investigating approaches and mechanisms to stabilize the thermodynamically metastable phases of TMDCs [99, 105].

Apart from utilizing the inherent properties of the materials, altering the mode of contact represents a methodology for optimizing contact resistance. VdW integration is thought to be a promising strategy for addressing the FLP phenomenon, which is attributed to the presence of surface dangling bonds and surface reconstructions. This integration mechanism facilitates the establishment of a physical connection between materials without direct chemical bonding. To date, several methods have been employed to realize vdW contacts, including the growth of buffer layers [106] and physical transfer techniques. For instance, Liu et al. demonstrated a method in which metal electrodes were fabricated on sacrificial substrates. Through a pretreatment procedure, the metal layers were detached from the sacrificial substrate using PMMA and then physically bonded to 2D semiconductors. This approach enabled the attainment of high carrier mobility levels that are compatible with vdW graphene hybrid contacts and strong contact doping [107]. Nevertheless, it is important to highlight that as devices continue to shrink in size, achieving precise alignment between materials becomes increasingly challenging. Additionally, the weak bonding of vdW contact may lead to the deviation of metal electrodes, consequently introducing variations in device performance [108].

Except for strategies mentioned above, novel techniques have been put forward to lower the contact resistance. Notably, in 2013, Wang et al. first introduced a novel methodology that used O_2_ and CHF_3_ plasma etching on a BN-graphene-BN stack, followed by the deposition of metal leads at the stack’s edge, establishing direct electrode contact with the edge of graphene. This strategic configuration facilitated more efficient carrier injection in comparison with conventional top contacts, thereby yielding reduced resistance [109]. Nevertheless, it is imperative to acknowledge that the stability and fabrication complexity of edge contacts present ongoing challenges [110], prompting extensive research efforts aimed at enhancing their performance, such as the combination of reactive ion etching, in situ Ar^+^ sputtering and annealing [111] and heterostructure growth [112]. Furthermore, an innovative filament contact approach has emerged as a promising avenue for achieving sub-10 mV dec^−1^ subthreshold swing (SS) and high on-current, thus offering a novel strategy for addressing the contact resistance issue [40].

In summary, as the prerequisite for harnessing the full potential of 2D materials, realizing good electrical contacts remains the focus field of 2D technology. Numerous strategies aimed at enhancing contacts have been proposed and have yielded significant results. Nevertheless, there are still several factors that demand consideration: (1) The scalability of contact methods, assessing whether the chosen materials or techniques possess broad applicability across different types of 2D materials while remaining cost-effective; (2) Uniformity and compatibility, ensuring that the selected methods compatible with other processes, maintain device uniformity during large-scale production and support circuit-level applications; (3) The scalability of these methods, as most research directions mentioned earlier still operate at the micrometer scale for electrode sizes. Given the ongoing trend of device size reduction, contact-related challenges will become increasingly prominent. Therefore, the development of devices with nanoscale footprints holds equal importance.

### Material Transfer and Integration

A plethora of studies have reported the fabrication of 2D material-based electronic devices with excellent performance, such as low contact resistance, low leakage current, high on-state current, and short *L*_g_ [69, 72, 76]. However, it remains uncertain whether such superior device performance can be replicated when scaling up to wafer-scale ICs produced through CVD. Due to the atomic layer thickness and delicate lattice of 2D materials, achieving large-scale integration of 2D devices should fully consider device yield, device-to-device variability, stability, and reliability. Such considerations must account for defects inherent to the 2D materials themselves, including vacancies, impurities, wrinkles, and thickness fluctuations, as well as external environmental factors such as changing trap states or residual organic materials. These fluctuations can result in deviations in device performance, ultimately leading to logic function errors, device failure, and decreased device yield [41].

**Fig. 6:**
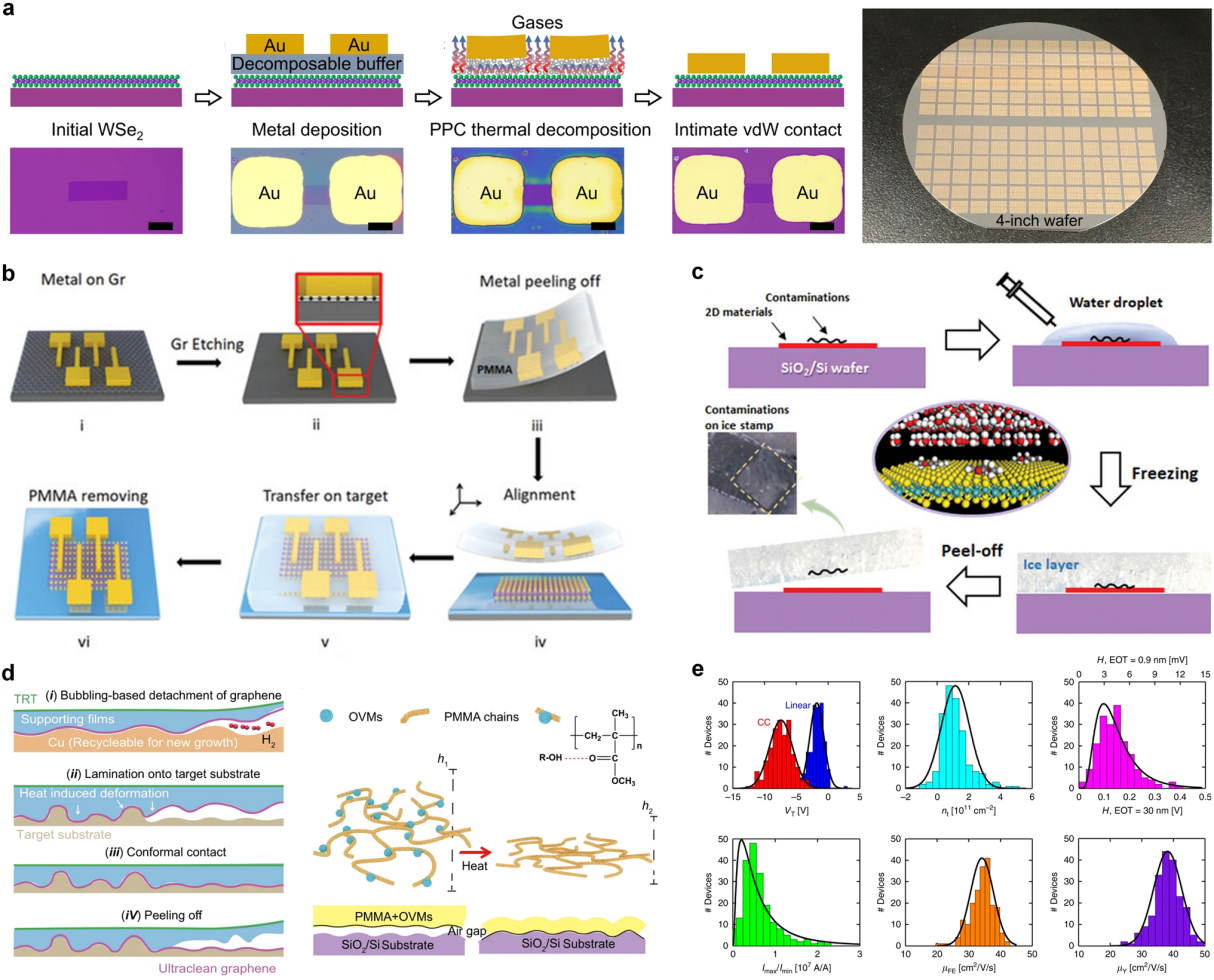
**a** Wafer-scale vdW integration processes with four steps and optical images of large-scale vdW contacts. Reproduced with permission [113]. Copyright (2023), Springer Nature. **b** Schematic illustration of graphene-enhanced vdWs integration. Reproduced with permission [96]. Copyright (2023), Wiley–VCH. **c** Illustration of the ice-cleaning process. Reproduced with permission [114]. Copyright (2023), Wiley–VCH. **d** Illustration of techniques and mechanism of the crack-free transfer techniques. Reproduced with permission [115]. Copyright (2022), Springer Nature. **e** Variability analysis of multiple parameters of hundreds of MoS_2_ FETs. Reproduced with permission [116]. Copyright (2017), American Chemical Society

So far, several strategies have been reported to form atomically flat interfaces and reduce the surface states from defects, residues, and strains on the 2D semiconductors. For example, to overcome the interface damage and FLP induced by high-energy metal deposition and lithography processes. Wafer-scale vdW integration method has attracted widespread attention. Liu et al. achieved vdW contact between metal and 2D semiconductor by spin-coating a decomposable poly (propylene carbonate) (PPC) as a buffer layer (Fig. 6a). And this method can be readily applicable to different metals and semiconductors [113]. Qi et al. demonstrated an approach of graphene-enhanced vdWs integration, which was realized by the physical transfer of 2D monolayer graphene/3D metal heterojunctions onto 2D semiconductors. The method exhibits a maximum resolution capability at the nanometer scale while yielding a device array with a near-perfect success rate of ~ 100% (Fig. 6b) [96]. The vdW integration method is characterized by its facile integration process, universal applicability, and capacity for enhancing device design flexibility. Nevertheless, the challenge of achieving higher alignment accuracy for small-sized electrodes and higher-density integration needs to be addressed. Moreover, the weak vdW forces demand careful consideration of potential structural deviation or performance degradation caused by subsequent processes, especially those involving high temperature. Additionally, the multiple steps of contact and release during the integration process may introduce unwanted defects or oxidation which necessitates further investigation and exploration of these issues.

In addition to the metal deposition, the presence of organic residue from polymer-assisted transfer process represents another source of defects. So far, the types of CVD growth substrates for 2D materials are still limited and the transfer process is indispensable. The most commonly used approach currently is to utilize PMMA as a supporting layer for protecting the 2D materials from tension and perturbation. And the insufficient removal of supporting polymer films will degrade the material quality [117]. Better cleaning methods should be developed. Zhao et al. incorporated oxhydryl groups-contained volatile molecules into PMMA, which can be deformed under heat. The optimized supporting films could achieve a controllable conformal contact without cracks, contamination, and wrinkles (Fig. 6d) [115]. In addition to improvements to traditional organic-assisted transfer methods, Liu et al. proposed novel ice-aided transfer and ice-stamp transfer methods, which utilize the controllable adhesion between ice and various 2D materials. And the new transfer methods can yield ultrahigh quality and exceptional cleanliness (Fig. 6c) [114]. Furthermore, dielectric integration is another critical factor that affects device yield. Several researchers have attempted to adjust process steps to improve the interface quality. The gate-first process, which fabricates all the critical components before the MoS_2_ transfer step, is believed to reduce variability effectively compared with gate-last process [118].

The integration process of 2D materials is exceedingly complex and currently immature, involving numerous process steps and parameters. On the one hand, it is necessary to establish an appropriate evaluation system to assess the impact of various parameters on device variability. Smithe et al. fabricated and measured hundreds of back-gate MoS_2_ FETs and gave a statistical evaluation of the key parameters (Fig. 6e) [116]. This can aid in designing appropriate redundancy in critical parameters and structures to mitigate the effects of variability. On the other hand, it is worth considering the incorporation of machine learning techniques to assist in analyzing and optimizing the preparation process of 2D materials, assisting in the selection of optimal preparation conditions, and accelerating the iteration speed of device processes.

### 2D Package

Electronic packaging is one of the essential steps in chip-scale integration, serving multiple functions such as signal interconnection and chip protection. However, due to the predominant focus of 2D materials research on the device level and small-scale circuits, there is still relatively limited research dedicated to the packaging of 2D chips. Considering the air sensitivity of 2D materials and their relatively limited voltage tolerance, research and development of 2D packaging technologies remain crucial. In this section, we provide a summary of the current research related to the packaging of 2D devices.

#### Signal Interconnection

Wire bonding was always used to achieve the electrical interconnection between 2D device and printed circuit board (PCB) since it was usually applied in small I/O numbers at low cost. Smith et al. [119] reported that the graphene pressure sensor and humidity sensors were wire bonded to the substrate in the ceramic dual inline package (DIP). Similar work has been reported in Susan’s work [120]. They used 25-μm-thick AlSi wires to connect the optoelectronic devices based on graphene with Ti/Ag contacts to PCB.

With the I/O of devices based on 2D materials increasing, flip chip technologies can be used in the packaging of 2D devices, which needs further study on the adhesion of 2D materials on the substrate. With more I/O and high performance needed, Chiplets [121], 2.5-dimensional integration [122], and three-dimensional integration [123] can also be adopted to achieve the large-scale integration and improve the integration density.

#### Chip Protection

Protecting layer before packaging: Compared to the traditional semiconductors, most 2D materials exhibit heightened sensitivity to water and oxygen when they are exposed to the ambient air owing to their elevated surface-to-volume ratio [124]. For example, it has been demonstrated that BP is highly reactive to oxygen and water exposed to ambient air, leading to degradation in the electronic and optical properties [125]. Consequently, the encapsulation strategies for 2D materials discussed in the work involve the creation of a protective layer that isolates 2D material from oxygen and water in the air to prevent the reaction.

The protecting layer encapsulated on 2D materials is primarily divided into organic and inorganic protective layers. Polymethyl methacrylate (PMMA) [126], polyvinyl pyrrolidone (PVP) [127], fluoropolymer CYTOP [128], and parylene C [129] were commonly used as the organic protective layers to protect 2D materials. For example, Jia et al. [130] and Li et al. [131] reported that the PMMA-encapsulated BP FETs exhibit high stability over several weeks and achieve a high field-effect mobility up to 1150 cm^2^ V^−1^ s^−1^. Besides PMMA, the other organic materials also process the same function to protect 2D materials in ambient air.

Besides organic protective layer, the inorganic protective layer was commonly used to protect 2D materials. Oxide (Al_2_O_3_) [132], zirconium oxide (ZrO_2_) [133], and hafnium dioxide (HfO_2_) [134] were the most widely materials with the deposition process of atomic layer deposition (ALD). For example, Li et al. [135] directly encapsulated MoS_2_ FETs with Al_2_O_3_ by ALD, and then, the MoS_2_-based FETs had a high I_on_/I_off_ ratio exceeding 10^8^ and the mobility around 70 cm^2^ V^−1^ s^−1^ at room temperature. ZrO_2_ and HfO_2_ are also used to protect h-BN and MoS_2_ to improve the device’s performance.

**Other protections in the packaging and its perspective:** When 2D devices are used in the high-performance memory, molding compound as the package must be used in the future. However, there is still a relatively limited amount of research. Effects of the warpage and stress during the molding process on the 2D devices are worthy of deep studying.

Electrostatic discharge (ESD) protection is another concern for the packaging of 2D devices. The nano-electronics-based 2D materials process high *I*_on_/*I*_off_ ratios in FETs [13] and band gap tunability [136]; thus, it has been widely used in low-power transistors [137], sensors [138], and photodetectors [139]. Meanwhile, the 2D materials also can withstand high breakdown voltage. For example, the breakdown voltage of MoS_2_ semiconducting gate (SG) FET is 408 V [140]. Thus, 2D devices provide a large design window for ESD protection.

However, there is a lack of strategies for ESD protection of 2D devices. Thus, we make prospects of ESD protection way based on Si devices: firstly, designing a switch around the device that can be precisely controlled such as a nanophase-switch [141] and gNEMS ESD switch [142] and secondly designing a protection structure in the PCB to discharge any ESD transients such as setting a mixed-mode ESD protection circuit [143] and dual-polarity nano-crossbar array ESD protection structure [144] of the device based on 2D materials. It is worth noting that 2D materials can withstand high breakdown voltage; thus, their ESD protection can be installed on PCB.

With the development of devices based on 2D materials, their package become more important for application. Therefore, we need more research on package of devices based on 2D materials to achieve the holistic development of 2D materials application.

## 2D Materials for Digital and Analog Chips

Integrated circuit (IC) technology breaks the separation of devices and wires in traditional electronic technology, leading the trend of electronic components and lines and even the whole system toward integration [1]. Among all kinds of circuits, digital and analog circuits are the two main streams. Compared to bulk silicon, the ability to achieve extremely thin channels is the most distinctive advantage of 2D materials [145]. This characteristic naturally provides 2D materials with atomically thin channel lengths, excellent electronic transport properties, lower carrier scattering, and tunable band structures [146] in the fabrication of digital and analog ICs, where these advantages offer unique opportunities and potential for the application of 2D materials in the field of IC.

**Fig. 7:**
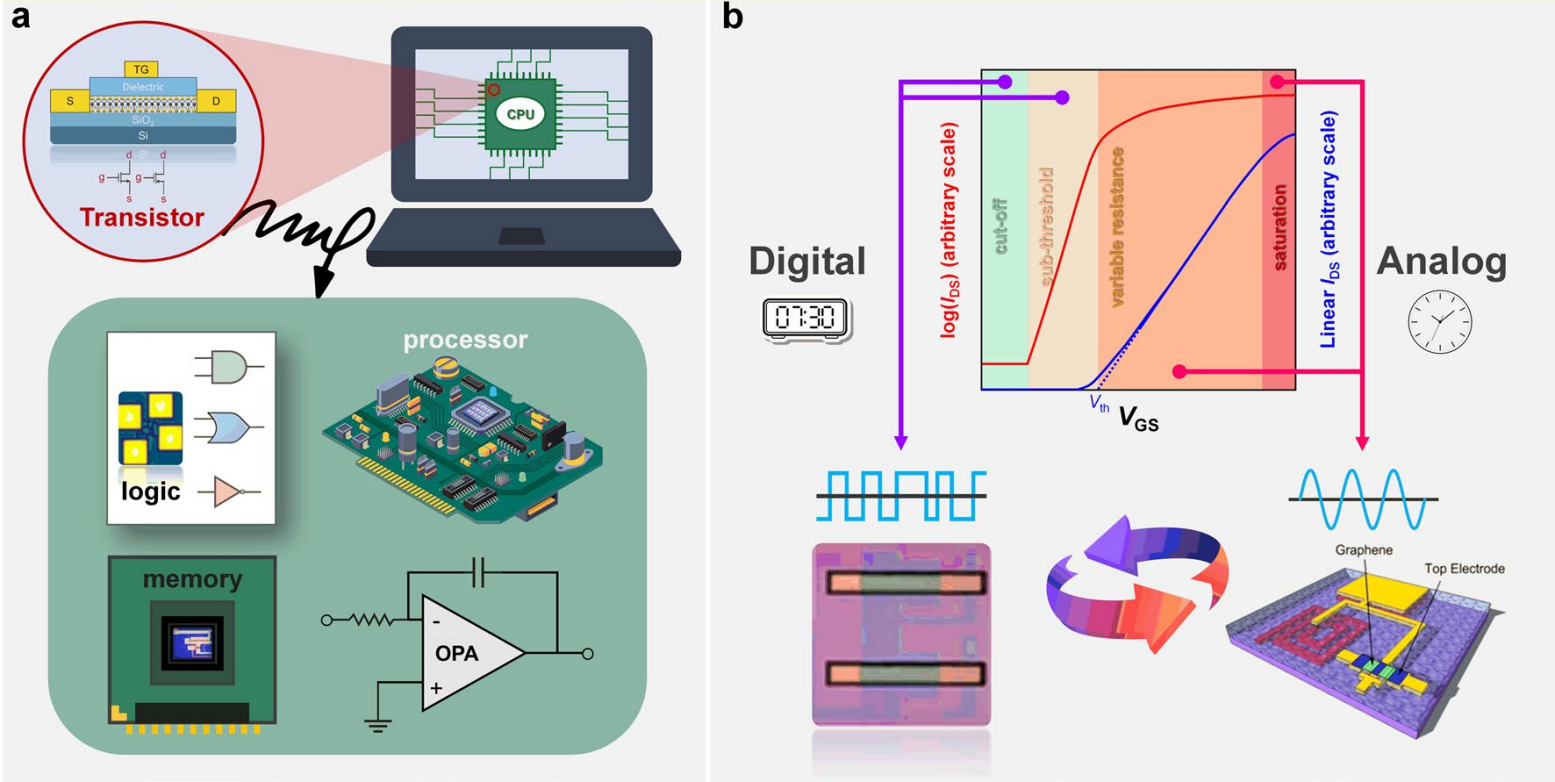
**a** Schematic illustration of the enlarged interior of the CPU and applications of transistors, where transistors serve as the dominant functional devices. **b** Transfer characteristic curve of transistors with four working regions suitable for digital or analog circuits: cutoff, subthreshold, variable resistance, and saturation. X-axis is the gate–source voltage, and y-axis is the drain–source current in logarithmic (left y-axis, red line) and linear (right y-axis, blue line) coordinates

Transistors serve as the dominant functional devices, whose performance is critical to circuits. Taking the central processing unit (CPU) as an example, as shown in Fig. 7a, the interior of CPU is almost N-type metal–oxide–semiconductor (NMOS) and P-type metal–oxide–semiconductor (PMOS) FETs, and other circuits can also be realized based on them. As shown in Fig. 7b, in *I*_DS_-*V*_GS_ curve, we can easily find that each gate-controlled transistor has four working regions. The cutoff region and the variable resistance region are often used in digital circuits to conduct on–off state converting, and the subthreshold region and saturation region are often used in analog circuits through special electrical connections. Based on transistors prepared with 2D materials mentioned above [72, 97, 101, 107], small-scale circuits and large-scale circuits with relatively complex data processing abilities can be fabricated. Furthermore, 2D materials can be easily combined with or stacked with other materials to form composite structures or heterostructures enabling the integration of multiple functions into a single chip.

In this section, we will first review the recent research progress of 2D materials in digital and analog circuits, respectively. Then, we will combine the development history of traditional silicon-based CMOS circuits to further summarize the status of digital and analog circuits based on 2D materials and give a design flow chart that can be used for future research. Based on the above content, we finally make an outlook for digital and analog chips based on 2D materials.

### 2D Digital ICs

Given the essential requirements of high switching speed and low power consumption in digital ICs [147], 2D materials offer significant advantages as they have high carrier mobility, excellent electron transport properties, mediate bandgaps, low electron scattering, as well as flexibility and scalability. Compared with analog ICs, digital ICs mostly use transistors, which is conducive to the completion of preliminary 2D circuit design, to establish logic gates with less utilization of capacitors and inductors, and different logic gates are then employed to achieve arithmetic computation. Therefore, the most likely form of mass production of large-scale circuits based on 2D materials is digital chips. The large-area synthesis of 2D materials and the continuous improvement of the performance of 2D transistors mentioned above have created important prerequisites for the fabrication of 2D digital ICs. However, the optimization of transistors is not the ultimate goal, and more important is the working performance of transistors in practical circuits. Therefore, in the past 5–10 years, more and more researchers focused on digital circuits based on 2D transistors, small-scale logic circuits [118, 148, 149], memory chips [150–153], microprocessor [20], and so on, which have been demonstrated.

#### Logic Function Realization

To achieve logic functions, the fundamental components of a circuit are various logic gates (AND, OR, NOT, XOR, XNOR, etc.). Since inverters are the most basic and simplest modules among various logic gates, we first start with some inverters based on 2D materials and review the research progress of small-scale logic circuits at the same time.

**Fig. 8:**
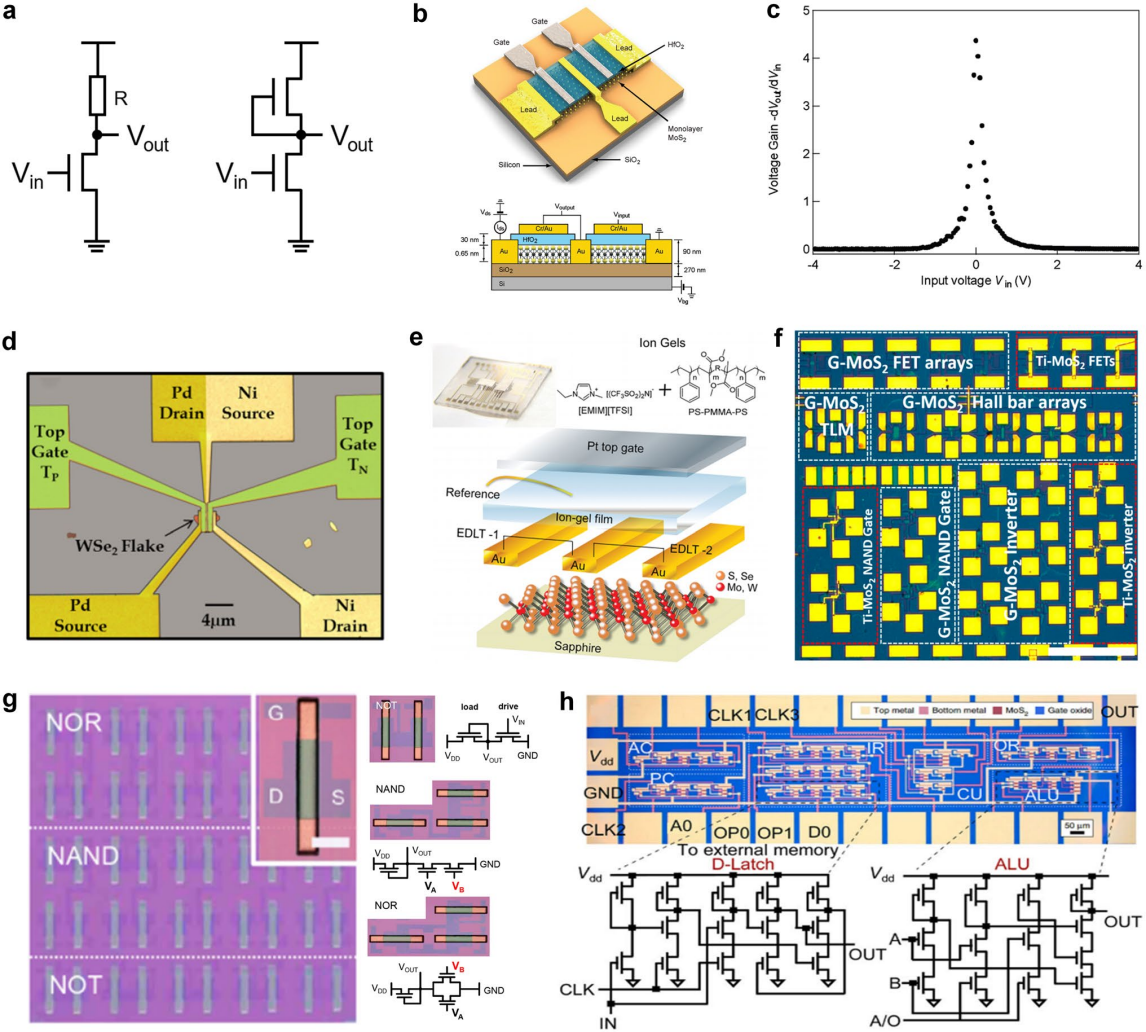
**a** Circuit schematic diagram of a resistor-load inverter and NMOS-load inverter. When the NMOS-load inverter works, only one transistor is active, and the upper transistor acts as a resistor. Similarly, the PMOS-load inverter has a similar structure. **b** Mechanically exfoliated monolayer MoS_2_ flake (upper) and the schematic illustration of monolayer MoS_2_ NMOS inverter (bottom). Reproduced with permission [167]. Copyright (2011), American Chemical Society. **c** Voltage transfer characteristics of a monolayer MoS_2_ logic inverter with corresponding voltage gain. Reproduced with permission [167]. Copyright (2011), American Chemical Society. **d** False color optical image of the inverter (scale bar = 4 µm). Reproduced with permission [158]. Copyright (2014), AIP Publishing. **e** Structure of ambipolar EDLTs with ion-gel thin-film coupling based on monolayer CVD-MoS_2_ onto a sapphire substrate. Reproduced with permission [159]. Copyright (2016), WILEY‐VCH Verlag GmbH & Co. KGaA, Weinheim. **f** Optical microscope image of the large-scale chip of MoS_2_ devices and circuits. The white dashed box is CVD graphene as electrodes and interconnects and the red dashed box is control devices and circuits using Ti/Au electrodes in adjacent. The scale bar is 500 μm. Reproduced with permission [164]. Copyright (2014), American Chemical Society. **g** Photos of large-area CVD-ReS_2_ logic circuits after patterning process (scale bar = 500 µm). Reproduced with permission [165]. Copyright (2017), American Chemical Society. **h** Optical microscope image of 1-bit MoS_2_ microprocessor with circuit schematics of D-Latch and ALU. The scale bar is 50 µm. Reproduced with permission [20]. Copyright (2017), Springer Nature

Inverters realized with 2D MOSFET mainly have three types, including NMOS-only, PMOS-only and CMOS. As shown in Fig. 8a, NMOS-only and PMOS-only inverters use two transistors of the same type above and below, and only one of these two transistors is energized at a time. The other transistor’s gate electrode and source electrode are shorted together as a pull-up or pull-down resistor load. CMOS inverter is formed by connecting the drain electrode of NMOS with that of PMOS. Graphene, as the first 2D material synthesized by humans [25], served as the foundation for early research on 2D logic circuits. Floriano Traversi et al. [154] fabricated a complementary logic inverter by integrating p-type and n-type transistors on the same monolayer graphene in 2009, where the Boolean inversion function was obtained by Dirac points modulation. Although this inverter’s voltage gain was only 0.044 and its operating bias was as high as 10 V due to the operation through a conventional silicon bottom gate, limited for direct cascading because of the bad drive capacity, it showed the feasibility of using graphene to form digital circuits. To make full use of the ambipolar feature of graphene for practical use, Song-Lin Li et al. conducted a variety of explorations. In 2010, the first example of graphene voltage inverters with voltage gain up to 4–7 was demonstrated by them [155]. They resolved the issues of bottom-gated-based graphene by employing high-efficiency top gate stacks with natural alumina as the dielectric layer. One year later, they developed graphene logic devices advanced than inverters with electrostatic doping method, introducing a bandgap into the channels and fulfilling elementary NOR and NAND logic gates on bilayer graphene (BLG) channels [156].

However, pristine graphene’s inherent zero bandgap structure will increase fabrication complexity if we need p-type and n-type transistors simultaneously. Early logic circuits adopted 2D transistors of the identical conductivity type and the fabrication of 2D NMOS transistors is more mature than that of PMOS [13, 157], so only NMOS technique was really popular at that time. Its simple circuit topology allows for fast switching times and low power consumption compared to other types of inverters that require complementary MOSFETs or ambipolar transistors. In 2011, Branimir Radisavljevic et al. [13] fabricated the first MoS_2_-based logic inverter by connecting two top-gated n-type 2D transistors, as shown in Fig. 8b, enhancing the voltage gain to 4 directly (Fig. 8c), in which single-layer MoS_2_ exfoliated from bulk crystals served as the channel material. This work provided ideas for the implementation of digital logic in 2D materials at room temperature and could open the way to using MoS_2_ for applications in flexible electronics. Indeed, the methods mentioned above are based on mechanical exfoliation, which is reasonable for achieving high-quality samples, especially for small-scale logic gates where large area coverage is not required. However, to realize large-scale circuits, mechanical exfoliation becomes impractical, and alternative approaches such as CVD need to be employed to achieve the growth of high-quality 2D films on a larger scale. Benefiting from the advancements in 2D material growth techniques, the first monolayer MoS_2_-based inverter and circuits using CVD growth were successfully fabricated [52, 148]. By connecting a MoS_2_ n-type FET in series with a MoS_2_ resistor, the inverter exhibited a voltage gain close to 20 and a wide voltage operation range from 0.5 to 5 V. Moreover, the MoS_2_-based inverter was also integrated with NAND logic gate, presenting the capability of realizing any Boolean logic functions.

Inverters made from single-polarity transistors have power dissipation and other limitations. Gradually, some researchers embarked on implying ambipolar 2D materials and complementary conductivity types of 2D transistors aimed at electricity energy saving, improved noise margin, wide voltage range, and so on. 2D MoS_2_ flake has been applied in NMOS-only circuits, but it is still challenging to create CMOS inverters based on MoS_2_. The pinning of metal Fermi level close to the conduction band of MoS_2_ prevents efficient hole injection into the valence band of MoS_2_, causing the unavailability of MoS_2_ p-type FETs [158]. WSe_2_, as another significant member of TMDCs’ family, is available for both electron transport and hole transport because metal Fermi levels are pinned close to the middle of its bandgap. In 2014, a fully complementary logic inverter based on a bilayer WSe_2_ FET by employing contact work-function engineering and electrostatic doping was demonstrated [158]. Saptarshi Das et al. used 20-nm SiO_2_ as the bottom gate dielectric to facilitate better electronic gate control and used Pd as the source/drain electrode for p-type FET and Ni as the source/drain electrode for n-type FET to gain better carrier injection from metal to WSe_2_, as shown in Fig. 8d. The maximum voltage gain was ~ 25, and the noise margin was close to its ideal value of ~ 2.5 V (V_DD_ = 5.0 V). Without connecting output and gate electrode to realize MOS-load, the identical ambipolar transistors based on TMDCs reduced power dissipation [149, 159]. Another example is shown in Fig. 8e, a quasi-CMOS inverter [159], where two single-layer WSe_2_ ambipolar electric double-layer transistors (EDLTs) were connected on the same wafer-scale film, was fabricated with maximum gain up to 80 (*V*_DD_ = 2.8 V, *V*_th_ = 1.4 V). Meanwhile, by combining p-type WSe_2_ and n-type MoS_2_ EDLTs, Jiang Pu et al. [159] also realized CMOS inverters among 2D materials, using ion gels as gate electrolytes, promoting the highest voltage gain of 110. In addition to utilizing the polarity characteristics of 2D materials themselves for complementary logic circuits, some researchers have recently published work on utilizing different h-BN thicknesses to modulate electrical conductivity of materials [160]. Furthermore, an increasing number of digital logic functions are being implemented on 2D materials [161–163], creating conditions for the advanced study of more complex circuits.

#### Complex Digital Functional Circuits

Complex 2D digital functional circuits, which build up a basic foundation for data processing, not only require a great number of logic gates to perform intricate logic operations but also need the establishment of combinational logic and sequential logic circuits. Therefore, the overall performance of the circuit in the case of cascades, timing, and feedback is a more important issue. To fabricate complex digital circuits, the first step involves integrating multiple logic gates together, and the following is the quantity or scale increase. Here, we present several representative works as examples to illustrate the process of implementing complex logic functions. Subsequently, we categorize and discuss the research progress in the areas of adders, static random-access memory (SRAM), and ring oscillators (RO). Finally, we provide a further overview of the largest and most functionally complex 1-bit microprocessor fabricated using 2D materials to date.

After optimizing the discrete electronic components and their basic logic function circuits, a significant next step is the fabrication of fully integrated multistage circuits for complex applications. In 2012, Han Wang et al. [150] demonstrated the fully integrated systems including an inverter, a NAND gate, a SRAM, and a five-stage ring oscillator based on a direct-coupled transistor logic technology. On a single sheet of bilayer MoS_2_, 2 to 12 transistors were seamlessly integrated side-by-side. In 2014, Lili Yu et al. [164] demonstrated a novel scalable all-2D-material electronics platform using both CVD-grown monolayer MoS_2_ and graphene, which is the first logic circuits using CVD-grown 2D heterostructures that are mass-producible. In that work, single-layer MoS_2_ served as the channel material, and graphene is used as both the electrode contacts and the interconnects for the circuit system, where the tunable Fermi level in graphene allows excellent work-function match with MoS_2_ and hence leads to low contact resistance. Figure 8f shows the demonstration of the large-scale chip containing MoS_2_-based NMOS inverter arrays and NAND gates (scale bar = 500 μm). In research [165], the gate voltage was reduced under 2 V by applying an ion gate as the gate dielectric with larger gate capacitance. Therefore, the realization of low power PMOS-only logic inverters was available. Figure 8g shows the large-area ReS_2_ transistors that were successfully assembled to fabricate logic devices, such as NOT, NAND, and NOR gates. The fabrication of logic gate arrays using large-scale growth of 2D materials through CVD holds significant importance in expanding the scale of 2D digital circuits. Similar to the current mature CMOS technology, the future development of 2D circuits also requires addressing challenges in material growth over large areas and the fabrication of large-scale circuits. Additionally, considerations need to be given to simulation modeling and circuit design processes. Lili Yu et al. [118] presented a feasible approach for large-scale fabrication of MoS_2_ circuits using CVD growth in 2016, focusing on the aspects of design, modeling, and fabrication. They initially utilized a gate-first process (all the components were built and optimized before transferring MoS_2_) to fabricate the highly uniform enhancement-mode FETs (with mobility as high as 80 cm^2^ V^−1^ s^−1^) and extracted device parameters to develop Verilog-A compact models for performance prediction. Then, by constructing this computer-aided design (CAD) flow, the researchers proceeded to design combinational logic gates and sequential logic circuits (AND, OR, NAND, NOR, XNOR, latch, edge-triggered register) as well as switched capacitor converter. The co-optimization of fabrication process and circuit performance paved the way for designers to further develop the potential of 2D materials. On this basis, further optimization is to increase operating speed and decrease the power dissipation with complementary logic circuits.

It is not sufficient to only achieve computational functions, and the vast amount of data generated during the computation process needs to be stored for subsequent retrieval and to facilitate further calculations. In the von Neumann architecture, a storage region is required to be integrated within the logic chip, which is commonly realized by using SRAM digital circuits. SRAM consists of a flip-flop and transmission gates, and the key to fabricating it lies in achieving stable storage units. To achieve stable storage, a low-power SRAM with 2 transistors and 2 resistors (2T2R) [152] and a wafer-scale SRAM and RO based on pseudo-NMOS [153] were successfully fabricated and tested. Here, RO is often used to generate a clock signal and provide the way for frequency measurement. The adder is another common small-size module in digital circuits, that is composed of multiple logic gates and flip-flops. Due to the similarity between these two modules, some researchers tend to realize adders and SRAM in one work. In 2022, a new advancement in monolithic 3D integration of CFET with 2D materials channels has been demonstrated [166], in which the 4-transistor SRAM and 16-transistor half-adder circuit units based on CFET design were experimentally realized for the first time. In this work, Xiong et al. used low-temperature post-metal annealing method to avoid the deteriorated performance of p-channel 2D transistors, with a record high *I*_on_ of ~ 594 μA μm^−1^ and *G*_m_ of ~ 244 μS μm^−1^ (*V*_d_ =  − 2 V, *L*_ch_ = 135 nm).

In recent years, some researchers have been exploring the possibility of using 2D materials as potential candidates for the next generation of IC chips. However, there have only been limited successful demonstrations of 2D microprocessor with 1-bit computing ability until now. In research [20], the 1-bit microprocessor consists of 115 transistors with different width/length ratios of gate electrodes. And the arithmetic logic unit (ALU) with inputs A and B, accumulator (AC), control unit (CU), instruction register (IR), output register (OR), and program counter (PC) were all fabricated on the same MoS_2_-based substrate, whose architecture is shown in Fig. 8h. The 1-bit microprocess dealt with CLK signal generation and memory in an off-chip way. But it’s a pity that there are only a few percent of the fully functional devices within the whole system, even if the yield for subunits was 80%, such as ALU. This is possibly due to mismatch problems resulting from uniformity issues between transistors, especially variation in *V*_th_. In this case, we still need to further improve the yield and uniformity of our 2D devices in large-scale systems practically.

### 2D Analog ICs

Despite the dominant market share and chip area occupied by digital circuits, analog circuits are indispensable due to the intrinsic continuity of signals in the physical world. Therefore, although digital circuits are gradually replacing analog circuits with the advancement of IC processing techniques, certain analog circuits such as amplifiers, analog-to-digital converter (ADC), clocking, and power management remain indispensable. However, as functions become diverse and density gets higher, the performance improvement of analog ICs with traditional silicon-based CMOS technology has encountered bottlenecks owning to noise and bandwidth limitation. Therefore, researchers aim to enhance the performance, functions, and integration capability by exploring new materials [168–170], device structures [171, 172], and circuit design techniques [173, 174].

Among various materials, 2D materials with atomic body thickness show prominent advantages in realizing analogue functions. For example, 2D materials offer unique electrical and optical properties, exhibiting excellent carrier mobility, high thermal conductivity, and low noise characteristics, which are beneficial for high-speed signal processing and low-power operation. Moreover, some 2D materials have inherent characteristics to simplify circuit design directly, such as ambipolar transport properties of graphene and tunable bandgap properties of BP. On the other hand, it is really necessary to further consider the completeness of 2D system-level circuits. So far, a series of researches on 2D digital circuits and sensors have been demonstrated. In the future, 2D materials will establish their development system, and analog circuits are essential to bridge the gap between sensing and computation. In this section, we focus on recent research progress toward the realization of analog electronics and circuits based on different 2D materials.

**Fig. 9:**
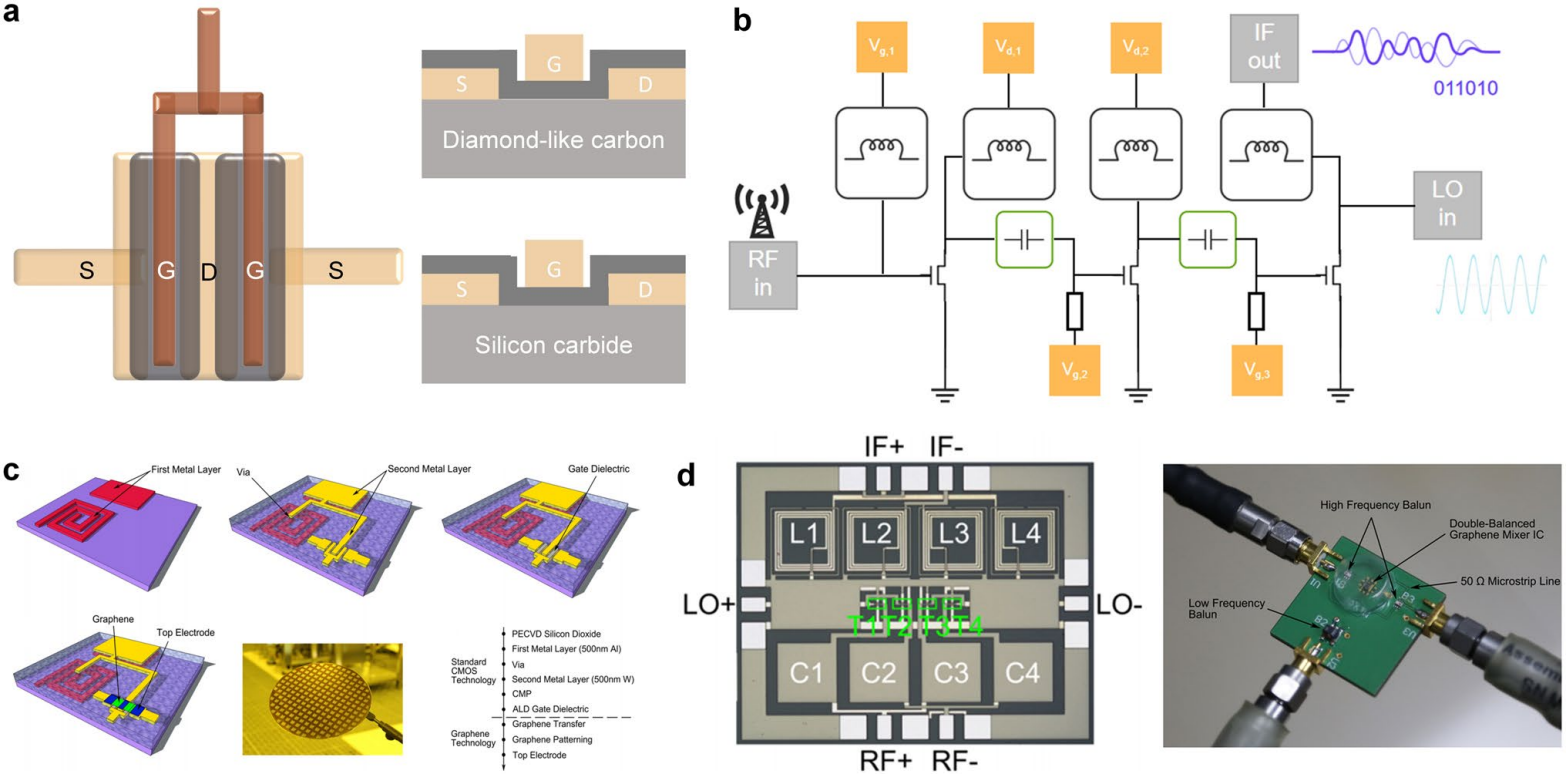
**a** Ground-signal-ground (GSG) pad design in dual-channel graphene RF transistor (on the left) and structure diagrams of devices on different substrates (diamond-like carbon & silicon carbide) (on the right). **b** Schematic diagram of graphene RF receivers including 11 active and passive components. **c** Schematic diagram of the fabrication process of the graphene IC with the inverted process, a photograph of the 200 mm wafer and the integration flow. Reproduced with permission [180]. Copyright (2015), American Chemical Society. **d** Optical image of the mixer (on the left) and fabricated double-balanced mixer on PCB (on the right). Reproduced with permission [180]. Copyright (2015), American Chemical Society

In terms of transistors in analog circuits, the continuous voltage and current signal can be processed and amplified, within the conversion from off state to on state. Graphene, with ambipolar transport properties, extremely high mobility [175] and its tunable bandgap through electric field modulation, can be used to develop a new form of nonlinear electronics for RF and mixed-signal applications. Han Wang et al. [176] fabricated a frequency-doubling device with just a single graphene FETs (GFETs) in 2009 for the first time. This frequency multiplier can give very high purity at the output without any additional filtering, which was back-gated by the p-type Si wafer (Fig. 9a), where its V-shaped transfer characteristic curve displays two polarities of carrier transport on both sides of the minimum conductivity point. Despite the limitation imposed by the 10 kHz input signal frequency, the guiding significance of this research is undeniable. Later, the fabrication of a new kind of single-transistor RF mixer device was demonstrated [177], which can effectively suppress odd-order intermodulations. The calculated *f*_T_ of these devices was about 190 MHz, limited by the device gate length and gate capacitance. In 2011, Yanqing Wu et al. [178] reported top-gated CVD-graphene RF transistors with gate lengths scaled down to 40 nm, leading to cutoff frequencies as high as 155 GHz. One year later, they enhanced the cutoff frequency of GFET above 300 GHz based on CVD-graphene grown on copper and epitaxial graphene grown on SiC [179], surpassing previous records. Based on the GFETs, a wafer-scale integrated graphene amplifier circuit with voltage and power gains reaching 20 dB was demonstrated. Additionally, graphene RF receivers [168] with a 16-finger T-shaped gate (Fig. 9b) and double-balanced graphene mixer IC [180] with passive-first-active-last fabrication flow (Fig. 9c, d) offer insights into on-chip integration strategies and demonstrate the potential to compete with other semiconductor technologies in RF front-end applications.

Nevertheless, the absence of a bandgap in pristine graphene hampers its on–off ratio, because it tends to exhibit linear output characteristics with limited current saturation, making it difficult to achieve high voltage and power gains. In parallel to graphene’s ambipolarity and tunable bandgap properties, BP has emerged as a potential candidate for high-frequency analog circuit applications [68]. Its high carrier mobility, which results in high transconductance and amplifier gain, combined with the ability to tune the bandgap width through thickness modulation, has garnered significant interest among researchers. In 2015, Saptarshi Das et al. [181] reported an analog small signal amplifier based on a BP load resistor and a BP FET integrated on a single flake. The signal gain of this amplifier was ~ 9 for frequency up to 15 kHz, and the direct current (DC) performance was observed with a record-high ON current of 200 μA/μm (*V*_DD_ =  − 0.5 V). In 2016, Sk. F. Chowdhury et al. [169] realized BP-based RF circuits including common source amplifier, mixer and amplitude modulation demodulator, totally working at megahertz range for the first time. However, the difficulty of large-scale synthesis of BP films and the stability of BP in air conditions impose restrictions on its further development.

While designing an analog circuit, we should consider trade-offs between factors, such as intrinsic gain, power consumption, noise, speed, and linearity [182]. TMDCs have excellent gate electrostatic control, resulting in constantly flat output curves for high output impedance and high intrinsic gain, which is crucial and beneficial to overcome device scaling-down problems. As a result, TMDCs are one of the most promising channel materials for future analog electronics. Traditional mechanical exfoliation method is not suitable for industrial-scale processes, and thus, researchers [183] and [184] chose to use monolayer CVD-MoS_2_ FETs to realize RF electronics and circuits. For better performance in high-frequency electronics, bilayer MoS_2_ with higher carrier mobility than that of monolayer can enhance performance owing to the higher density of states and small bandgap. To this end, Gao et al. [170] dealt with the problems of large domain bilayer CVD-grown MoS_2_ by adjusting the weight of MoO_3_ precursor on molten glass during growth. Recently, increasingly complex analog circuits based on MoS_2_ have been successfully implemented, including operational amplifiers (OPA) [185], current mirrors [172, 173], and phase-locked loops (PLL) [174]. OPA, which demands high uniformity of constituent components strictly, is a basic building block of analogue electronics. In 2020, Dmitry K. Polyushkin et al. [185] demonstrated the first operational amplifier circuits (64 OPAs) based on 2D MoS_2_ as the active material from the aspect of design, fabrication and characterization. Each OPA contains 12 n-type enhancement-mode FETs with low variability. In the same year, a MoS_2_-based current mirror [173] was fabricated by Shunli Ma et al., with the measurement results of the current gain from 2.72 to 171. They also constructed an industrial design flow for the design, simulation, and layout of MoS_2_-based chips, which will provide a general idea of the process from the laboratory to the factory.

However, it should be noted that the current state of research in analog domain is still relatively nascent, with a wide range of analog circuit types awaiting further exploration and development. The continuity of analog signals makes them highly sensitive to changes in the external environment, while 2D materials have high surface area-to-volume ratios and sensitivity, making them prone to interference from external temperature variations and noise. Therefore, further development of standardized techniques and complementary packaging technologies is needed.

**Fig. 10:**
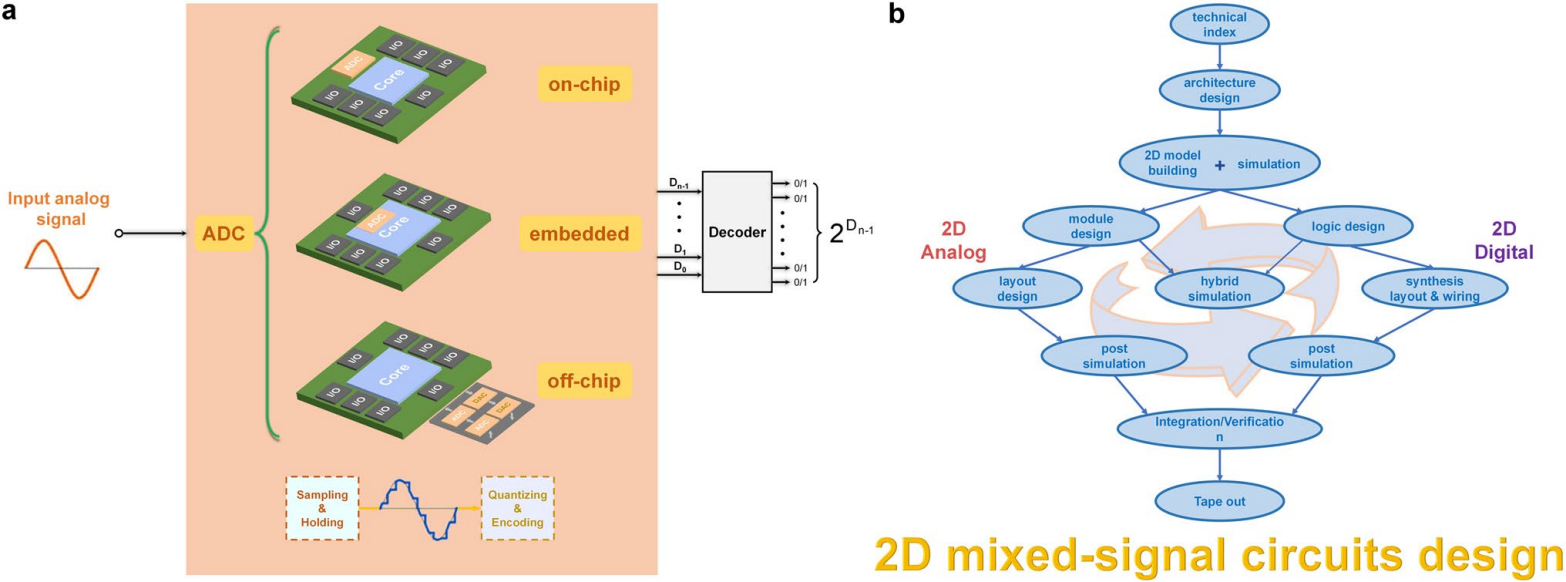
**a** Diagram of several connection forms between the core circuit and the ADC converter: on-chip, embedded, off-chip. The analog signal with continuous amplitude is sampled, maintained, quantized and encoded by the ADC converter and then output to the decoder to generate the binary value. **b** Feasible design process of 2D mixed-signal circuits

Comparing the developmental trajectory of 2D materials with silicon-based circuits [67], it becomes evident that the progress in developing essential devices for large-scale digital and analog circuits based on 2D materials is similar to silicon-based technology To a certain extent, the design of circuits based on 2D materials is similar to that of silicon-based circuits because both of them consist of layered stacking in layout design softwares, but with some different fabrication processes. Silicon-based circuits rely on doping for polarity control, whereas 2D materials exhibit unique advantages as they have two kinds of conductivity in the 2D material library. Furthermore, it is noted that future research will focus on the implementation of mixed-signal circuits based on 2D materials, so that subsequent circuits will depend on ADC to realize signal transformation, in other words, to quantize values within a certain range. As shown in Fig. 10a, some researchers are dedicated to designing 2D materials-based ADC on-chip circuits [148] and addressing integration challenges [172] due to the limited space available and the need for isolation between analog and digital components, making digital–analog mixed circuits come true. Other efforts are put into exploring embedded ADC converters [186] and other assembly methods such as off-chip connected ADC converters [187]. Here, we provide a valuable reference for the design process of 2D mixed-signal circuits, as shown in Fig. 10b.

Nevertheless, although there is a positive trend in the development of 2D digital and analog ICs, it is undeniable that numerous limitations and technical challenges still exist. In the realm of 2D digital circuits, firstly, the circuit scale is still limited by the synthesis area and quality of 2D materials. Secondly, different from silicon-based circuits via processing, the multilayered metal interconnects of 2D materials may cause damage to the underlying films, and on-plane interconnections between devices can result in crosstalk. Thirdly, improvements are required in device yield, uniformity, and the integration of modules such as storage and clocks, which currently rely on external connections. In the realm of 2D analog circuits, firstly, the design process demands high expertise from researchers, necessitating collaborative efforts between material researchers, device researchers, and design researchers. Besides, the lack of Process Design Kits (PDK) and design tools results in longer design periods. Secondly, implementing devices with excellent performance based on only 2D materials, such as bipolar junction transistors, inductors, and capacitors still poses difficulties. Thirdly, present research efforts largely focus on achieving specific functions of analogue devices, leaving considerable space for enhancing performance parameters. To achieve silicon-like performance, the performance metrics of 2D transistors used in digital and analog circuits need critical assessment and accurate measurement. For example, widely used device parameters in research papers (such as carrier mobility and contact resistance) could not be reliable because of frequent misestimation [28]. To this end, in Table 1, we show a set of key performance parameter values of silicon-based and 2D FETs in circuits. Although 2D digital and analog circuits have yet to demonstrate obvious advantages over that of silicon, their significance will increase as the technology progresses toward sub-1 nm nodes.

**Table 1:** Performance metrics of 2D FETs and silicon-based FETs in circuits

Parameters	Explanation	2D material-based FETs^1^		Silicon-based FETs^2^
		Fundamental Indicators	Advanced Indicators	
*I*_on_ (mA·μm^−1^)	$I_{{{\text{DS}}}} = \frac{{W_{{{\text{ch}}}} }}{{2L_{{{\text{ch}}}} }}\mu_{{{\text{FE}}}} C_{{{\text{ox}}}} [2(V_{{{\text{GS}}}} - V_{{{\text{th}}}} )V_{{{\text{DS}}}} - V_{{{\text{DS}}}}^{2} ] ({\text{Linear}}\;{\text{ region}}) I_{{{\text{DS}}}} = \frac{{W_{{{\text{ch}}}} }}{{2L_{{{\text{ch}}}} }}\mu_{{{\text{FE}}}} C_{{{\text{ox}}}} [(V_{{{\text{GS}}}} - V_{{{\text{th}}}} )^{2} ] ({\text{Saturation}}\;{\text{region}})$	0.5 (LP), 1 (HP)	2 (LP), 3 (HP)	0.6 (LP), 1.5 (HP)
*V*_DD_ (V)	supporting the high level (1) voltage potential for logic gates	1.4	0.5 ~ 0.7	0.65
*v*_sat_ (cm·s^−1^)	limiting the operating frequency of the device	0.5 × 10^7^	1 × 10^7^ ~ 2 × 10^7^	1.6 × 10^7^
*R*_ds(on)_^3^ (Ω·μm)	including *R*_Cds_, *R*_Css_, *R*_series_ metal–semiconductor contact resistance	400	150 ~ 200	172
*μ*_*FE*_^4^ (cm^2^ V^−1^ s^−1^)	$\mu_{{{\text{FE}}}} = \frac{{L_{{{\text{ch}}}} g_{m} }}{{W_{{{\text{ch}}}} C_{G} V_{{{\text{DS}}}} }} = \frac{{L_{{{\text{ch}}}} }}{{W_{{{\text{ch}}}} C_{G} V_{{{\text{DS}}}} }} \cdot \left. {\frac{{dI_{{{\text{DS}}}} }}{{dV_{{{\text{GS}}}} }}} \right|_{{V_{{{\text{DS}}}} = {\text{const}}}}$	200	1000	100
*SS* (mV·dec^−1^)	${\text{SS}} = \frac{{{\text{d}}V_{{{\text{GS}}}} }}{{{\text{d}}\lg I_{{{\text{DS}}}} }}$ influencing switching speed	65	60 ~ 62	65
*f*_T_^4^ (GHz)	$f_{T} \approx \frac{{g_{m} }}{2\pi }\frac{1}{{(C_{{{\text{GS}}}} + C_{{{\text{GD}}}} )[1 + g_{{{\text{ds}}}} (R_{S} + R_{D} )] + C_{{{\text{GD}}}} g_{m} (R_{S} + R_{D} )}}$ the frequency when the voltage gain is equal to 1, representing the maximum bandwidth that a device is able to achieve	150	350 ~ 450	340
*f*_max_^5^ (GHz)	$f_{\max } = \frac{{f_{T} }}{{\sqrt {4R_{G} (g_{ds} + 2\pi f_{T} C_{{{\text{GD}}}} )} }}$ the frequency when the power gain is equal to 1	150	400 ~ 500	370

HP: High Performance. LP: Low Power. V_GS_, V_DS_: terminal d.c. voltages. I_DS_: drain-source current. W_ch_: channel width. L_ch_: channel length. C_ox_: oxide dielectric capacitance. C_G_: gate capacitance. gm: intrinsic transconductance. C_GS_: gate-source capacitance. C_GD_: gate-drain capacitance. gds: drain conductance. R_S_: source series resistance. R_D_: drain series resistance. R_G_: gate resistance.

^1^The 2D materials here do not specifically refer to one single type of 2D material. Fundamental Indicators: the performance that 2D materials inherently possess and have been reached in the literatures. Advanced Indicators: the performance that have not been realized or is difficult to achieve at present. These two types of indicators are listed as references

^2^The data of *I*_on_ in the “Silicon-based FETs” column derives from the 2021 target in the ITRS 2015 report [188] and the data of radio-frequency performance parameters (*f*_T_ and *f*_max_) derives from a stacked Si nanosheet [189]

^3^*R*_ds(on)_ is the on-state resistance of the FET, including the drain-semiconductor contact resistance (*R*_Cds_), the source-semiconductor contact resistance (*R*_Css_), and the series resistance (*R*_series_)

^4^The calculation expressions of *μ*_FE_ and *f*_T_ are from [190]. In the expression for *μ*_FE_, *C*_G_ is the gate capacitance per unit area. ^5^The calculation expression of *f*_max_ is from [191]

## Heterogeneously Integrated 2D Devices and ICs

The big data era has increased the demand for computing systems that can perform functions beyond the capabilities of single-purpose chips. In response to this challenge, heterogeneously integrated technology is emerging as a promising solution. This technological approach entails the stacking of two or more distinct materials in a specific manner, resulting in the formation of a distinctive structure with adjustable properties. The properties of a heterostructure can be adjusted by modifying the composition, thickness, and stacking sequence of its constituent layers.

Conventional silicon-based technology is faced with several challenges, which include the need for scaling down to tackle problems like short channel effects, high leakage current, high power consumption, and high contact resistance [192, 193]. Therefore, it is imperative to replace conventional electrodes, channel materials, and dielectrics with advanced materials and structures that can alleviate these limitations. To this end, 2D materials and their heterostructures are considered reliable alternatives. As mentioned above, 2D materials can maintain low leakage characteristics at a thickness of just one atomic level, resulting in transistors with high drive current and low energy consumption [194]. In addition to traditional ICs, electronic systems with multiple functions can be achieved by integrating a new generation of circuits through 2D heterogeneous integration. For example, in the realm of optoelectronics, photodetectors prepared through heterogeneous integration enable both light-sensing and computing functions. Furthermore, heterogeneously integrated displays can drive micro-LEDs (μ-LEDs) using the good photoelectric performance of 2D FETs. The method of heterogeneous integration has improved many properties of multifunctional applications, such as the capability of photoelectric detection systems to capture light perception and process optical information. Furthermore, circuits made of 2D material FETs can improve problems such as linear amplifier distortion on analog circuits.

In this section, we examine work on 2D heterostructure devices and circuits that provide insight into how the tunable properties of 2D materials and their heterostructure can enhance performance. We address recent studies in terms of representative 2D materials heterostructures, the fabrication process, and the crucial role of physical parameters that affect the quality of 2D heterostructures and their IC.

### Heterogeneously Integrated 2D Devices

Heterogeneously integrated 2D devices are composed of two or more different 2D materials that are integrated as channel materials to form a functional device.

These devices are created by stacking layers of 2D materials with different physics/chemical properties to create interfaces with unique electronic, optical, or mechanical properties. The integration of these different materials allows for the creation of new types of devices with enhanced or novel functionalities that cannot be achieved through a single material or structure alone, such as complementary field-effect transistors. This configuration can reduce the area by 42%-50% compared with conventional layouts [79]. It could be utilized to further increase device density in ICs. However, silicon-based CFETs have several limitations that can cause performance degradation. These limitations include extra demands on the thermal budget, difficulties with epitaxial growth of the source, complex integration processes, and compensation of electron/hole mobility mismatch and V_th_ tuning. This last issue is well suited to be solved by the incorporation of 2D semiconductors, as their mobility and V_th_ can be easily tuned to match with another material [195]. It is worth mentioning that a 2D-CFET with a GAA structure has been invented, which provides a potential way for high-performance and low-power electronic applications. In this review, we classified 2D heterostructure CFET based on the process flow, device structure, and channel material. This CFET can be classified into laterally integrated CFET, vertically integrated CFET [79, 195, 196], and GAA CFETs [197].

#### Complementary Field-Effect Transistor 2D Heterostructure

The CFET, which stands for complementary field-effect transistor, signifies a progressive device paradigm in the realm of three-dimensional vertical integration, building on the advancements made in FinFET technology. In CFET units, the vertical stacking of p-n-type field-effect transistors is employed through three-dimensional integration, resulting in a substantial reduction in the layout area of ICs. Furthermore, this approach effectively reduces the negative impact of parasitic effects and interconnect overheads. It achieves this reduction by simplifying access to the transistor terminals through the internal shorting of the n/p drain contacts, which improves high performance, low power consumption, and minimizes area requirements. It is worth mentioning that devices with 2D materials as channels also have the aforementioned characteristics. The weak van der Waals force between layers of 2D materials makes them a promising channel material in stacked nanosheets (NS) and CFETs [194]. As 2D materials with flat surfaces and no hanging bonds, high mobility, and low leakage characteristics can be maintained at a thickness of just one atomic level. This property is advantageous for transistors as it allows for high drive current, and low leakage current, and effectively reduces energy consumption [198]. Additionally, these materials can be integrated with various types of substrates at low temperatures, without the need to consider lattice mismatch. 2D transition metal dihalides exhibit properties that are matched to low power applications, as their electronic energy gap is close to 2 eV, resulting in off-state currents down to the (Fa μm^−1^) order of magnitude, high performance and low energy consumption of transistors can be achieved [194].

Despite the continued miniaturization of traditional silicon-based transistors, their performance is limited by the interaction between the I_ON_ and I_OFF_. Specifically, as the transistor size decreases, an increase in I_ON_ is often accompanied by a corresponding increase in I_OFF_, resulting in a significant increase in power consumption. Therefore, 2D materials are more advantageous in terms of channel material selection and more compatible [196]. Compared to materials such as Si, 2D materials are more suitable for stacking at lower temperatures, which is compatible with 3D-integrated backchannel processes. Furthermore, the preparation and layer shifting of 2D materials can also be carried out at lower temperatures, which ensures the utilization of 2D materials in 3D-stacked heterogeneous CFET devices.

Notably, the scaling of 3D-stacked heterogeneous CFET devices is hindered by several significant barriers, including complex integration processes and challenges in reducing thermal budgets. Challenges arise in the optimization of contact resistance in 2D transistors. Higher current densities flowing through atomically thin materials result in significant thermal effects. In addition, it is worth noting that 2D materials exhibit distinct thermal conductivities in both the in-plane and between-plane models. In the in-plane model, it is observed that 2D materials exhibit favorable thermal conductivity. However, the thermal conductivity of a stacked structure of 2D materials decreases when they are stacked together. Furthermore, the integration of p-type 2D materials into high-performance FETs, logic gates, and ICs poses significant challenges. This is primarily attributed to the limited availability of large-area-grown p-type 2D materials [166]. The modulation of carrier mobility in p-type and n-type FETs is a crucial aspect in addressing the mobility mismatch between electrons and holes [195].

In order to further advance the scaling down of complementary FETs, three established scaling methodologies are recognized. The first approach involves substituting CFETs with a novel structure to facilitate additional scaling down. However, the potential for improving gate topology is limited. The second strategy entails reducing the EOT. However, gate leakage current becomes untenable when physical thickness scaling falls below 3 nm. The third and feasible alternative for overcoming the CFET scaling barrier involves reducing the thickness of the semiconductor channel. However, for the traditional Si channel, when the body thickness is below 5 nm, the mobility drastically degrades with the sixth power of thickness (μ ∝ tb^6^) owing to thickness-fluctuation-induced scattering, which poses a critical limit to the continued transistor scaling [28]. Future CFETs necessitate channel materials with superior mobility and lower leakage current in small footprints. 2D semiconductors with pristine surfaces will be one of the most promising candidates for further scaling, as they can maintain high mobility and low leakage current at an atomic-scale thickness [197].

Vertical stacking of atomic layer thin channels has been a significant challenge in the field of nanoelectronics. This challenge is primarily due to the complexity of integrated top-gate dielectrics and the associated fabrication processes, which result in deteriorated device performance. In 2021, Xiong et al. [79] demonstrated a novel approach to address this issue by vertically stacking MoS_2_/WSe_2_ complementary field-effect transistors that achieved a 50% reduction in footprint, thereby opening up new opportunities for monolithic 3D integration. However, the deterioration of the p-type bilayer transistor using a top-gate structure remains unresolved. In 2022, Xiong et al. [166] improved upon this work by successfully integrating CFETs with 2D materials channels for low-power ICs, solving the longstanding problem of poor electrical properties that prevent p-type and n-type devices from being matched. Specifically, the authors optimized the top gate p-channel bilayer WSe_2_ transistor fabrication processes using low-temperature post-metal annealing, achieving record-high *I*_on_ of ~ 594 μA μm^−1^ and *G*_m_ of ~ 244 μS μm^−1^ at *V*_d_ =  − 2 V with a short *L*_ch_ = 135 nm. Furthermore, the authors demonstrated the first-ever 4T SRAM and 16T half-adder circuit units based on a 2D heterostructure CFET design, presenting its superiority in performance, power, and area.

**Fig. 11:**
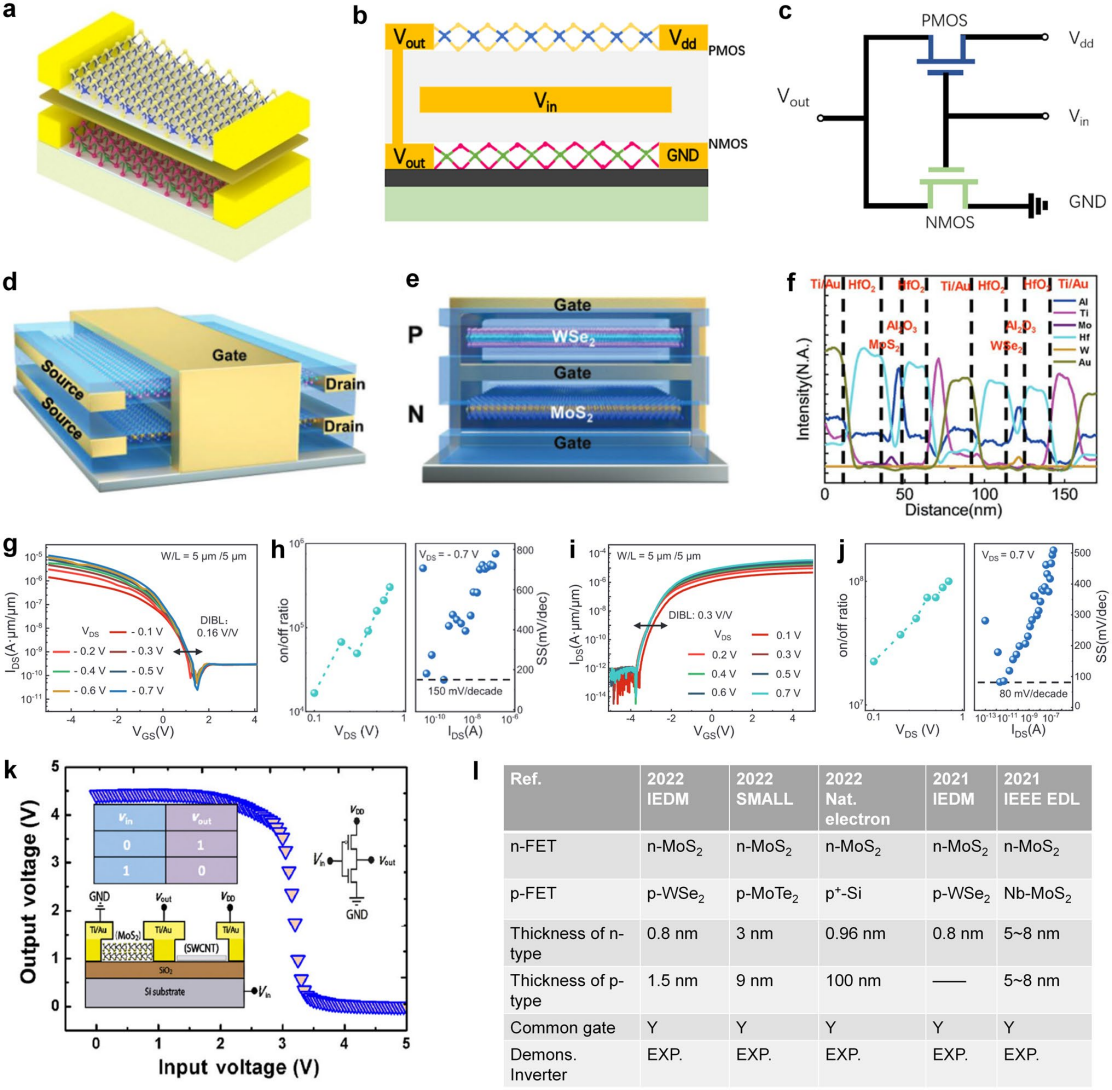
**a** Structure schematic of the vertical stacked CFETs. **b** Structure schematic of the MoS_2_/WSe_2_ CFET. **c** The corresponding equivalent circuit diagram of CFET. **d** Schematic structure and cross-sectional view of the 2D-CFET device structure. **e** The top WSe_2_ FET and bottom MoS_2_ FET are stacked vertically and share a common gate with a gate-all-around structure. **f** EDX line scan across the gate-all-around structure (Fig. 11e). **g, h** Electrical properties of the top WSe_2_-NS-p-FET in the 2D-CFET. Transfer characteristics at the source–drain voltage from − 0.1 to − 0.7 V. Current on/off ratio versus *V*_DS_ (left) and SS versus *I*_DS_ at *V*_DS_ =  − 0.7 V (right). **i,j** Electrical properties of the top WSe_2_-NS-p-FET in the 2D-CFET. Transfer characteristics at the source–drain voltage from − 0.1 to − 0.7 V. Current on/off ratio versus *V*_DS_ (left) and SS versus *I*_DS_ at *V*_DS_ =  − 0.7 V (right). Reproduced with permission [197] Copyright (2023), John Wiley and Sons. **k** Output voltage as a function of the input voltage for the MoS_2_/SWCNT heterogeneous inverters. Reproduced with permission [199] Copyright (2016), Springer Nature. **l** Comparisons of monolithic 2D CFET devices in previous studies [194, 200–203]

However, there remain challenges that need to be addressed in fully silicon-based systems, such as balancing electron and hole mobility and addressing the high thermal budget. Ling Tong et al. [195] presented a study on a 3D-stacked heterogeneous CFET containing SOI-based p-FETs and MoS_2_-based nFETs, presenting an integration approach that leverages the maturity of the silicon process, the low thermal budget of MoS_2_, and the low aspect ratio of the device structures to reduce process complexity and device degradation. The authors argued that thickness modulates carrier mobility to match, thereby alleviating the mobility mismatch between electrons and holes in n-FET/p-FET, that is the reason why we take thickness into account in Fig. 11i. In addition, further improvement of the SS can be achieved by reducing the thickness of the gate oxide and channel.

In addition to using 2D material as the channel material of the CFET, a heterogeneous structure combining 2D material and 1D material as the channel material of the CFET also has its advantages, such as high ON/OFF ratios up to 10^6^. As shown in Fig. 11k, Zhixin Li et al. [199] prepared heterogeneous complementary inverters by adopting bilayer MoS_2_ as an n-channel FET and SWCNTs as a p-channel FET. The n-channel MoS_2_-FET exhibited an ON/OFF ratio greater than 10^6^ and a high carrier mobility of 19 cm^2^ V^–1^ s^–1^, and the p-channel SWCNT-FET had an ON/OFF ratio of nearly 10^5^ and a large ON current. A schematic of the inverter is shown in the insets of Fig. 11k. This device achieves fundamental logic functions, exhibiting a maximum voltage gain of 15, a minimum power consumption of approximately 10 nW, and a noise margin of 0.45 *V*_DD_. Furthermore, the optimization of width-to-length ratios for the CFET presents a promising way to enhance power efficiency, rendering the device well-suited for prospective logic-circuit implementations.

#### GAA Nanosheet Vertical-Stacked CFET

NS vertical-stacked complementary field-effect transistors, where the NS n-FET and NS p-FET are vertically stacked and controlled using a common gate, would result in maximum device footprint reduction [200]. The 3D schematic view and cross-sectional view of the GAA nanosheet vertical CFET device structure are shown in Fig. 11d, e, respectively. Here GAA structure is regarded as a contributing factor to the high drive current and low leakage current. As shown in Fig. 11e, the n-FET and p-FET are vertically stacked and are controlled using a common gate, allowing the device to achieve maximum footprint reduction. M. Liu et al. [197] demonstrated a GAA NS vertical-stacked CFET based on chemical vapor deposition 1L MoS_2_ NS FET and WSe_2_ NS FET. The *I*_DS_–*V*_GS_ characteristics of bottom MoS_2_-NS-n-FET and top WSe_2_-NS-p-FET with channel width/length = 5/5 μm in fabricated 2D-CFET at different drain voltages (*V*_DS_) from 0.1 to 0.7 V are shown in Fig. 11g, i, demonstrating that 2D-CFET’s extremely strong current control capability at one-atomic thickness channel, which is shown more obviously in the extracted relationship between on/off ratio and V_DS_ bias in Fig. 11h, j. Furthermore, the extracted *D*_it_ values are 9.7 × 10^11^ and 4.3 × 10^12^ cm^−2^ eV^−1^ for n-FET and p-FET, respectively. These values are lower than other CVD-2D-based CFETs, attributing to the GAA NS structure and the clean fabrication process, highlighting a potential method for high-performance transistor scaling. This demonstration of vertical stacked NS CFET could pave the way for high-performance and low-power electronic applications. We believe that there is an urgent need for such studies to realize ideal device schemes in 2D CFET heterostructures and interfaces.

The outlook for stacked heterogeneously integrated 2D devices includes footprint reduction and lower power consumption. This approach effectively reduces the negative impact of parasitic effects and interconnect overheads in devices, showing great promise in terms of further scaling down. However, challenges such as thermal management, manufacturing costs, and reliability need to be addressed. The properties of a 2D heterostructure can be adjusted by modifying the composition, thickness, and stacking sequence of its constituent layers. With advancements and innovation, stacked heterogeneously integrated 2D devices are expected to play a crucial role in next-generation high-performance device configurations.

### Monolithic Heterogeneously Integrated 2D ICs

In comparison with traditional circuits that primarily use single-channel materials, heterogeneous circuits have been considered a suitable and reliable strategy for multi-function applications and performance optimization of conventional chips. In addition, 2D materials are among the most promising candidates for the next generation of electronic systems. The potential benefits of combining the unique advantages of 2D materials and traditional circuits on a monolithic large scale could lead to intriguing outcomes, such as combining sensing and computing in one system, reducing power consumption, and enhancing operation speed. In this section, the design of heterogeneously ICs will be discussed, with a focus on both high- and low-density interconnects at the circuit level.

#### Monolithic 2D ICs Heterogeneously Integrated of Low Density

Low-density heterogeneous ICs can combine the excellent properties of 2D materials, such as ultrathin channels and weakened short-channel effects, with data processing circuits to achieve a variety of functions. The 2D materials family exhibits numerous outstanding electrical properties. In this section, we will list classic works that integrate 2D materials with traditional circuits while maintaining a low density of interconnect. Additionally, the potential development prospects for this field will be discussed.

**Fig. 12:**
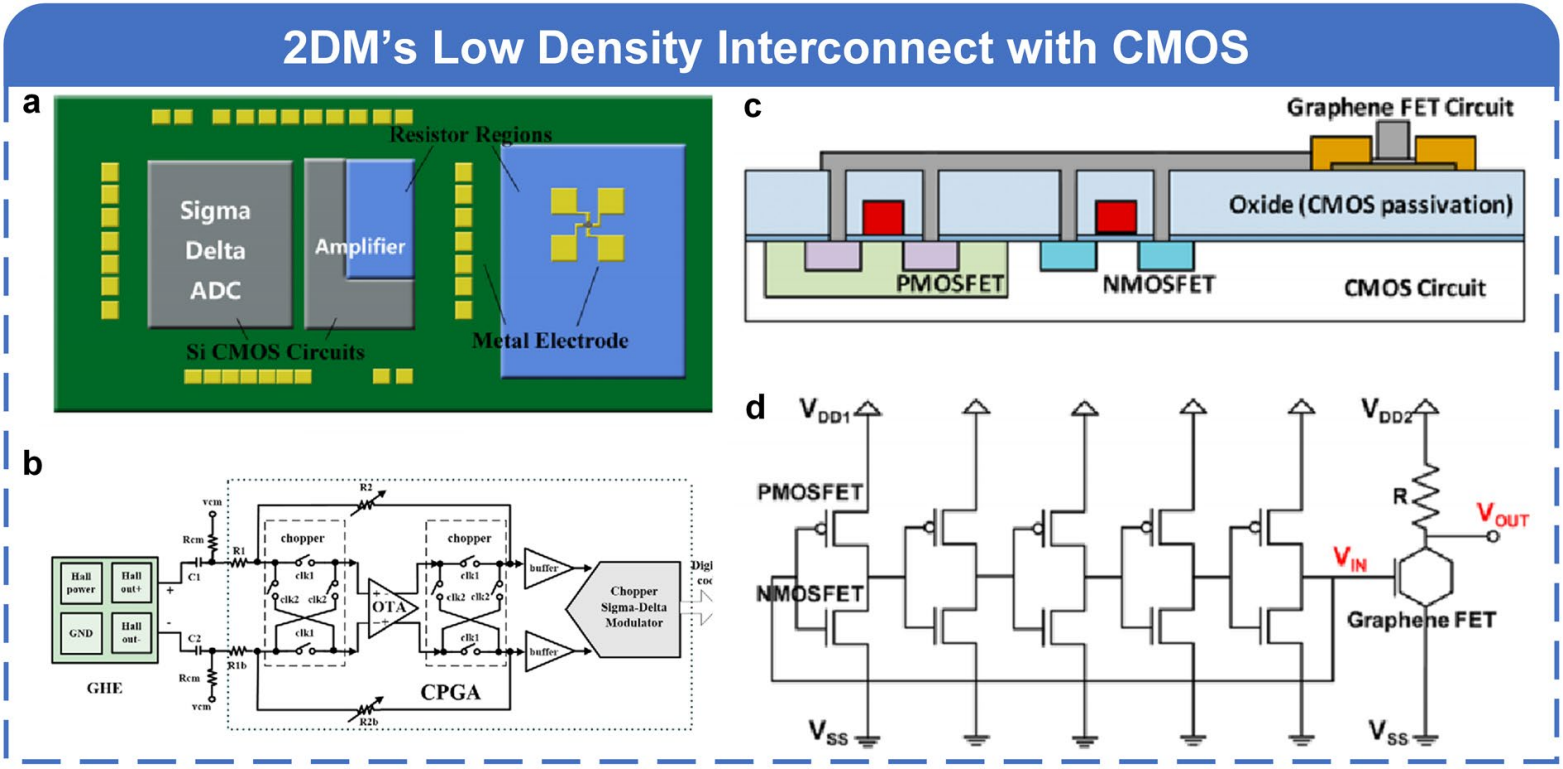
**a** Structure design of GHEs with CMOS ICs. **b** Circuit design of GHEs with CMOS ICs. Reproduced with permission [203]. Copyright (2020), American Chemical Society. **c** Structure design of integration of 2D FET analog circuit and CMOS digital circuit. **d** Circuit design of 2D FET analog circuit and CMOS digital circuit. Reproduced with permission [204]. Copyright (2016), American Chemical Society

The graphene Hall elements (GHEs) have attracted much attention due to their excellent sensitivity, low noise, nice linearity, and temperature stability. However, the performance of chips made only of GHEs is far from being suitable for commercial use. By heterogeneously integrating 2D materials with Si-based circuits, the performance of such chips will be improved. Here, Tongyu Dai et al. [203] achieved a method of three-dimensional building structure using heterogeneous integration of silicon-based CMOS ICs. They demonstrated a heterogeneous integrated Hall ICs that exhibit current and voltage magnetic sensitivities up to 64,000 A V^−1^ T^−1^ and 6.12 V V^−1^ T^−1^, respectively. The structure of this device and its circuit design are shown in Fig. 12a, b. Using an optimized fabrication process and refined design of processing ICs, the hybrid integration of GHE and Si CMOS ICs shows advantages in presenting higher performance and more functions than a single chip alone.

GHEs play an essential role in magnetic field sensors. In addition, graphene FETs also have strong advantages in analog applications due to their high carrier mobility and high saturation velocity. In order to fully utilize advantages of both graphene FET circuits in analog ICs and CMOS FET circuits in digital ICs, Seul Ki Hong et al. [204] utilized the 3D hybrid integration technology to fabricate an analog–digital mixed signal circuits on a chip. As shown in Fig. 12c, d, the researchers fabricated a graphene FET multimode phase shifter onto a silicon CMOS FET ring oscillator. The mode of the graphene phase shift can be easily controlled by adjusting the bias voltage of both the CMOS circuit and the graphene inverter. The utilization of this technique presents an opportunity to leverage the exceptional inherent properties of 2D materials and the advanced state of existing silicon CMOS technology, thereby facilitating the development of future electronics.

The successful fabrication of an analog–digital mixed signal chip is just the beginning of heterogeneous integration between 2D materials and conventional circuits. Although silicon-based chips dominate the semiconductor industry, their application in analog ICs is limited due to the inherent quadratic control relationship of FET, which distorts the amplification. To achieve high linearity in a FET, the extra complexity of inevitable circuits is necessary for conventional chips. In order to overcome the nonlinear distortion in Si-based analog ICs, Liangliang Xu et al. [205] reported the integration of graphene FET and Si-FET components. They successfully engineered a graphene-silicon hybrid analog amplifier IC with excellent linearity characteristics. It’s worth emphasizing that the judicious fusion of 2D materials with established Si-based technologies is a recommended approach.

Other than the low density of heterogeneous ICs, it is worth mentioning that we have found a lot of large area 2D FET arrays with high density. These works offer a positive outlook for high-density hybrid ICs. Due to its high on/off current ratio, weakened standby current, and near theoretical limit subthreshold slope, the 2D FET exhibits high speed and low power consumption. It is suitable for building processor and logic-in-memory circuits. A micro-processor consisting of 115 transistors was realized by Stefan Wachter et al. [20]. Guilherme Migliato Marega et al. [206] showed that 2D materials can enable scaling below 12 nm and increase reliability of chip with a high ON/OFF current ratio. Yikai Zheng et al. [207] used 2D-material-based memtransistor to fabricate a space-saving and lower energy consumption of Bayesian networks. In the field of near-sensor security, Akhil Dodda et al. [208] demonstrated an 8 × 8 crossbar array of fully integrated crypto engines, with each engine having 5 monolayer MoS_2_ memtransistors. In short, the research on large area 2D FET arrays could offer more opportunities for future heterogeneous ICs.

With the development of large-scale ICs using 2D materials, we will have a greater opportunity to achieve heterogeneous structures. However, what cannot be ignored is that there are still a lot of challenges waiting for solutions in fabricating hybrid chips. Completing the complex process of transferring a large area 2D materials is a significant challenge that restricts the number of research groups capable of exploring more efficient solutions for high-density interconnects. In addition, 2D materials have excellent optoelectronic performance, including tunable bandgap and strong light-matter interaction. In the field of photoelectric chips, the display system using 2D materials or other hybrid circuits requires a high-quality 2D FET array. Therefore, the connection between 2D FETs is crucial. Achieving high-density integration of 2D FETs is not an easy task. There is still a long way to go for the application of hybrid circuits in real life.

#### Monolithic 2D ICs Heterogeneously Integrated of High Density

We have discussed the low density of 2D materials interconnects with Si-based circuits. However, for complex tasks, a greater number of FETs will be required. Thus, low-density interconnect will no longer satisfy the needs of electronic applications, particularly in heterogeneously integrated optoelectronic systems. Many large-scale arrays of 2D FET arrays have been utilized in image sensors and micro-LED displays. There is potential for high-density 2D materials interconnect with traditional circuits.

**Fig. 13:**
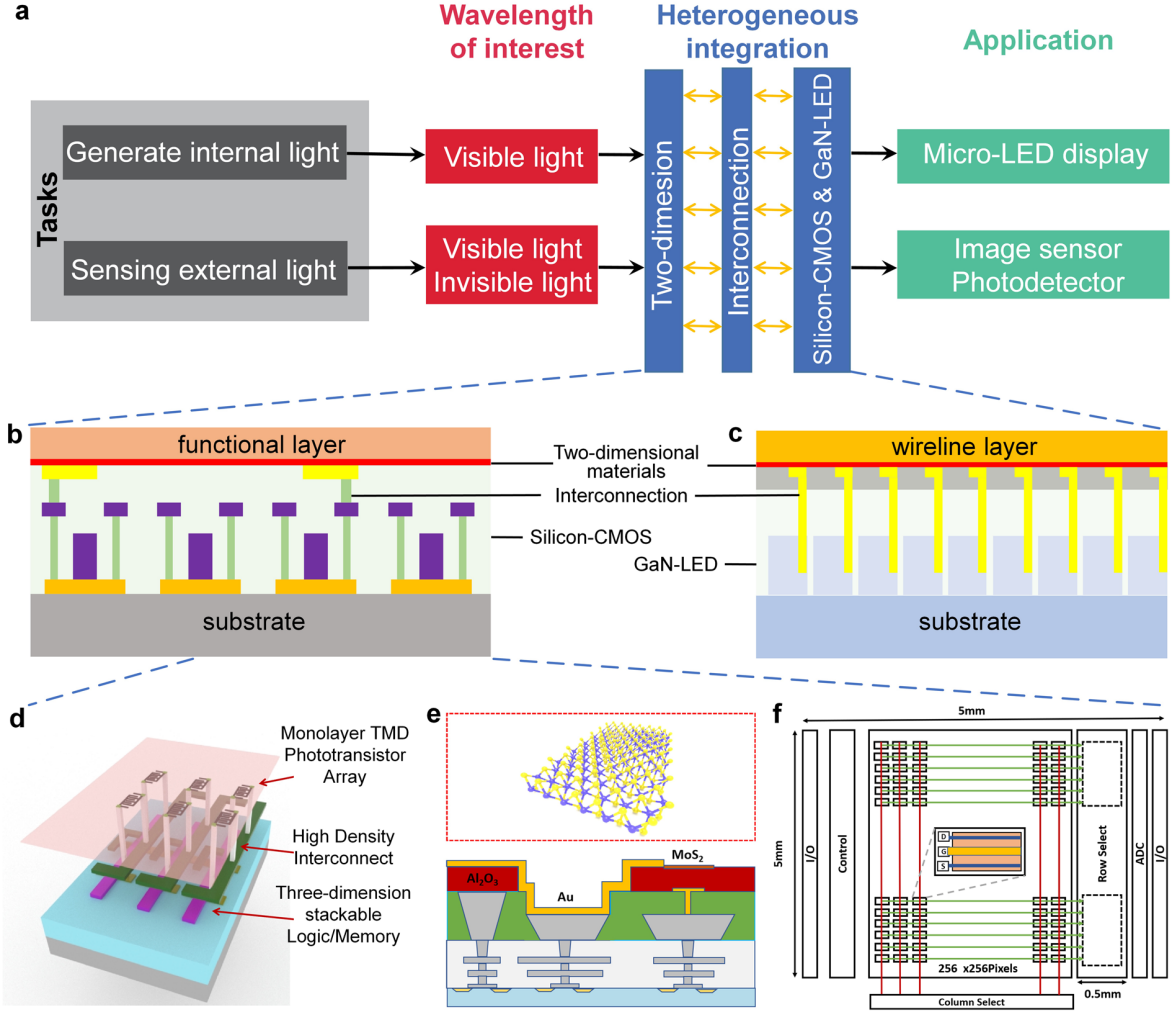
**a** Application in photoelectron when using 2D materials. **b** General heterogeneous integrated structure of sensing external light. **c** General heterogeneous integrated structure of generating internal light. **d** Structure of hybrid 3D^+^IC image sensor. **e** Structure design of the image sensor. **f** Circuit design of the image sensor

To better exploit the advantages of 2D materials, their exceptional photoelectric properties are used to fabricate high-density heterogeneous integrated optoelectronic chips. In the field of optoelectronics, heterogeneous ICs of 2D materials play an important role. As shown in Fig. 13a, we suggest that main function of electronic chips in the field of optoelectronics is to generate and sense light. The heterogeneous integrated chips that produce light are mainly in the visible light band, such as miniature LEDs and high-resolution displays. The perception of light is mainly through photodetectors, and these photodetectors are no longer limited to detecting only visible light. As shown in Fig. 13b, based on the works in optoelectronics, we have concluded a simplified structure for high-density interconnection. Its distinctive feature is three-dimensional stacking.

The development of microcircuits and visible-light cameras has been impeded in the past due to the challenge of integrating non-Si electro-optical materials with Si-based circuits. However, combining 2D materials with Si-CMOS is an advisable approach to achieve imaging capabilities beyond the visible range, on-chip low-power optical data communications, and compact sensing systems. In 2017, Stijn Goossens et al. [209] presented an image sensor that heterogeneously integrated graphene with CMOS technology. Their work reopens the door to heterogeneously integrated structures, utilizing a 388 × 288 array of high-quality graphene-quantum dot photodetectors. The resulting image sensor possesses high-mobility phototransistors, which have been interconnected with traditional CMOS circuits. This integration shows a broadband image sensor with compact and cost-effective advantages. This device exhibits an ultrahigh gain of 10^8^ and excellent responsivity above 10^7^ A W^−1^, which is a huge progress for quantum-dot-based imaging systems. In contrast to current methods of heterogeneous integration, there are no fundamental limits to shrinking the pixel size and enhancing resolution of the image. This work is a milestone for low-cost and high-pixel broadband imaging systems.

Integration of 2D materials and Si-based FET could combine the advantages of both technologies. This structure enables the low power and low-cost monolithic chips. The bulky indirect bandgap of Si is not suitable for portable and flexible electronics. Additionally, organic or polymeric detector arrays have not been certified for combination with Si-FET backplanes [210]. However, ultra-thin monolayer TMDCs with various energy bandgaps could be combined with a Si-FET backplane to enhance the performance of the circuit. In 2016, Chih-Chao Yang et al. [210] demonstrated a monolithic three-dimensional (3D) image sensor, which made use of a TMD phototransistor array heterogeneously integrated with a 3D logic/memory hybrid structure. As shown in Fig. 13d, this 3D image sensor has a high-density interconnect. On the top of this device, the monolayer MoS_2_ phototransistor shows excellent responsivity above 20 A W^−1^ and exhibits an outstanding linear response to the incident laser power density. At the bottom of this device is a 3D stackable silicon-based FET with a steep subthreshold swing and high driving current. The author obtained a TMD phototransistor array and a 3D stackable silicon-based FET, both of which have demonstrated excellent performance individually. Furthermore, the high interconnection density within them allows for a combination of their advantages, resulting in the rapid emergence of portable and flexible electronics.

It is promising that the performance of image sensors will be enhanced by 2D semiconducting materials in the near future. Using TMD instead of a Si photodiode array on a chip could produce diverse spectral sensitivities, surpassing the capabilities of a Si CMOS image sensor. Because of TMD monolayer crystals, they can exhibit different bandgaps or absorption wavelengths from infrared to visible. In 2022, Henry Hinton et al. [211] developed a 256 × 200 image sensor. They did not use additional layers, such as quantum dots, for light absorption [209]. Instead, they utilized TMD monolayers above CMOS time-to-digital converters. This chip relies on MoS_2_ photo-FETs to convert incident light into a photocurrent, which is then read out by CMOS time-to-digital converters. The cross-section schematic of a pixel is shown in Fig. 13e, and its 51,200-pixel circuits and 200 row-wise time-to-digital converters are shown in Fig. 13f. The design is clearly depicted in this floor plan. In the field of image sensors, the use of 2D materials will foreseeably continue.

While 2D materials play an outstanding role in image sensors or photodetector, they are also used in the production of micro-LEDs. Micro-LEDs have attracted much attention because of numerous advantages, such as quick response and low power consumption. Furthermore, due to the excellent electric performance of 2D FETs, the micro-LED can exhibit superior properties, such as improved resolution, high-speed operation, and increased brightness limit. Wanqing Meng et al. [212] have reported a monolithic heterogeneously integrated active-matrix display with a resolution of 1270 pixels per inch. In this design, the team utilizes a fast and bright micro-LED driven by 2D FETs. It only requires less than 8 V that could have 7 × 10^7^ cd m^−2^ luminance. It is worth mentioning that the backend of the line in this device’s production is at a low temperature and compatible with the traditional microelectronic manufacturing process. The different heterogeneous integration approaches enable the fabrication of micro-LED chips to have a higher chance of reaching a higher stage. In order to improve the bonding between LED chips and the backplane circuit, Sumin Hwangbo et al. [59] utilized a method that involved the direct growth of MoS_2_ on a GaN epitaxial wafer and the printing of quantum dots. This method was used to demonstrate a full-color micro-LED display using MoS_2_ transistors. The production yield of micro-LED pixels was approximately 92%. In addition, by the method of vertical stacking, Jiho Shin et al. announced a full-color micro-LEDs with the smallest size (4 μm) and highest array density (5, 100 pixels per inch (PPI)), reported to date (2023–02-01) [213]. 2D FET, which can drive micro-LEDs at low voltage and reduce the power consumption, is proposed as a heterogeneous component in this field.

Worth mentioning, that in addition to the image sensor and micro-LED display, 2D materials also play an important part in photonic chips due to their adjustable bandgaps. Fabrication of MoS_2_-based high responsivity photodetectors has already been reported [214], but an integrated photonic chip with 2D materials is still lacking. This gap has been bridged by heterogeneous integration between MoS_2_-based photodetectors and Si_3_N_4_ photonic circuits. Juan Francisco Gonzalez Marin et al. [215] demonstrated the integration of MoS_2_-based high responsivity photodetectors with Si_3_N_4_ photonic circuits. The chip that owns photoresponse times below 1 ms is achieved by using a h-BN substrate. Additionally, graphene local gates are used and low power operation is achieved in the final chip. A fast operation speed and using low operation voltages photodetector is made. Usage of 2D materials in different electronic systems will be continue.

Through heterogeneous integration, a new way to reach excellent device performance has been established. Maybe in the near future, we could see an increasing integration of compound semiconductors and 2D materials in various chips. And now, the primary application of this heterogeneous structure is in optoelectronics systems. Therefore, finding another field to apply this idea is crucial for the development of 2D materials hybrid integration.

In summary, the transfer of high-quality, large-area 2D materials remains a major technical challenge that limits chip performance and the creation of high-quality interconnects within circuits. Specifically, the challenge lies in integrating p-type 2D materials into high-performance FETs, logic gates, high-quality arrays, and ICs due to the lack of large-area growth of p-type 2D materials. For the device level, there are also challenges in the performance deterioration after integrating the top gate dielectric, the difficulty of reducing the thermal budget, and how to optimize the carrier mobility of p-type and n-type FETs. For the circuit level, improved interconnections for transistors and enhanced design in the driver and functional sections are required to obtain high-quality arrays.

Technology of heterogeneous integration that combines 2D materials and conventional circuits has realized multi-function and exhibited excellent performance in a single chip, such as technologies combining sensing and computing with low power consumption and fast operation speed. Furthermore, manufacturing processes of heterogeneous integration are compatible with conventional silicon-based processes. These hybrid process flows are damage-free for devices.

Conventional silicon-based circuits are facing difficulties in scaling down. To overcome this challenge, heterogeneous integration with 2D materials emerged as it can enhance space utilization rate. Besides, the advantages of 2D materials and silicon-based CMOS can be exploited this way. In the near future, more hybrid chips will be based on 2D materials and CMOS circuits, such as image sensors that exhibit fast speed, high sensitivity, and wide band. In addition, a mixed-signal chip fabricated by integrating 2D materials with silicon-based circuits can present fast operation speed with linear amplifier. Furthermore, heterogeneous integration of 2D materials and mature silicon-based technology has potential in building wide band sensor, fast in-memory computing, and advanced CMOS logic circuits in one chip.

## 2D Materials Sensor Chips

In the era of the Internet of Everything (IoE), sensors determine the comprehensiveness, accuracy and ease of access to information, and for this reason sensors are known as the essential technology to be developed in the future. Recently, complex applications such as artificial intelligence have placed higher demands on sensors’ number, type, and performance to enable large-scale data collection, storage, and analysis. Dimension scaling is a crucial technology for solving the high cost, high power consumption, and low integration of sensor networks [216]. To this end, the new generation of sensors is developing toward intelligent sensor chips. Although conventional silicon-based sensor chips have been heavily researched and commercially used [217], they usually face restrictions such as short-channel effects, thermal dissipation, gate modulation, long response times, and insufficient sensitivity [218]. To address these issues, researchers have turned their attention to 2D materials with atomic layer thicknesses [219]. The utilization of 2D materials in sensor technology offers the distinct advantage of exhibiting high sensitivity, rapid response, tunability, and high stability [220]. With the rapid development of wafer-scale 2D material growth technology and transfer technology, 2D material sensor chips have made remarkable progress in the past decade.

**Fig. 14:**
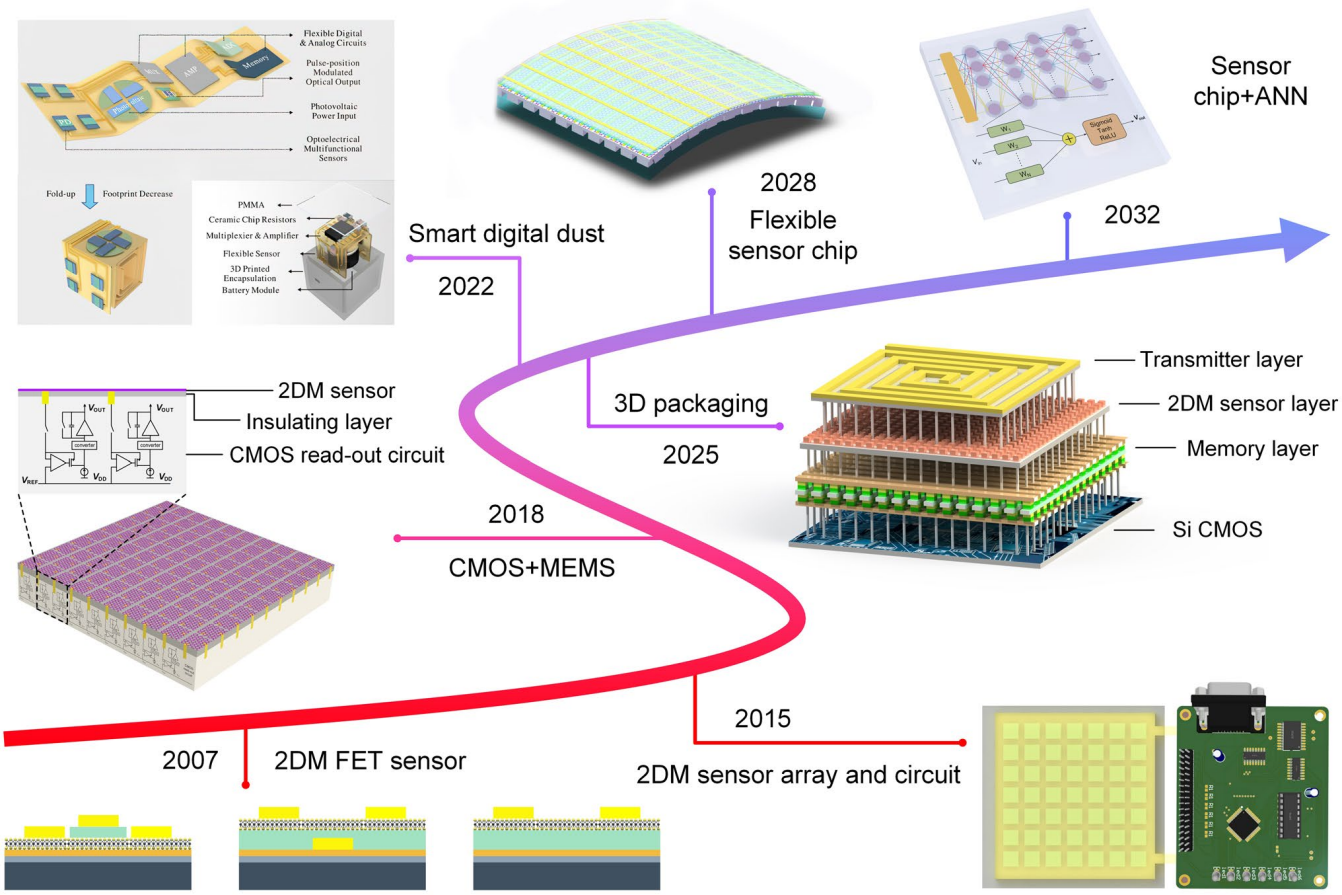
Developments of 2D material sensors toward chip. After the discovery of graphene, individual 2D material sensors with different gate structure were also demonstrated in 2007. The advent of 2D materials sensor arrays in conjunction with an external processing circuit in 2014 has increased the scale and utility 2D materials of sensors. In 2018, the combination of CMOS and MEMS processes has led to a reduction in the area of 2D materials sensor chips. In 2022, Mei et al. proposed the concept of digital micro-dust to achieve a high-density integrated, low-cost distributed 2D materials sensor chip. Reproduced with permission [222]. Copyright (2022), Elsevier. In 2025, the emerging 3D packaging technology will overturn the distribution structure of sensing networks and open a new era of ultracompact intelligent sensing systems. In 2028, 2D materials sensor chips will evolve toward full flexibility for enhanced sensing capabilities and close cooperation between humans and machines. In 2032, budding sensor technologies integrate sensor networks and advanced algorithms on a chip to reduce the significant power consumption required for transmission and computation

As is known to all, the most basic unit of the chip is the FET and the development of a 2D material sensor chip also starts from the 2D FET sensor, as summarized in Fig. 14. 2D materials FET sensors not only convert specific physical quantities into electrical parameters, but also can modulate the performance by divergent kinds of gates and are considered a fast, sensitive, and easy-to-operate advanced detection technology [221]. The primary focus of this phase is to assess whether the sensor performance of an individual device aligns with the established standards and validate the theoretical feasibility of the sensor chip. Subsequently, with the advent of silicon-based chip processing techniques, 2D material FET sensors have been progressively advancing toward the realization of large-scale arrays. Arrayed sensors offer significant advantages over individual sensors in terms of accuracy and information, while also enabling richer functionality, such as visual imaging of multiple physical quantities such as light, heat, and force. One of the primary challenges faced by arrayed sensors lies in the realm of large-scale signal processing. There are two primary methodologies: one involves computer-based data collection and processing, while the other entails connecting it to external circuits for achieving functions such as data acquisition, amplification, and transmission. On this basis, external circuits are replaced with specialized chips and combined with sensor arrays to obtain more minor distributed sensor chips. Smart dust is a typical representative of one of these devices, integrating sensors, digital and analog circuits, RF chips and power supply modules [222], although its size has been reduced to the size of a grain of sand. Considering that 2D material sensors predominantly employ micro-electro-mechanical system (MEMS) processes, the integration of MEMS and CMOS processes on a single chip is also highly desirable. The chip comprises two fundamental modules: the CMOS area, encompassing peripheral circuits such as control and signal processing circuits; and the MEMS device area, primarily responsible for the fabrication and processing of 2D material sensors. In the post-Moore era, as chip manufacturing processes gradually approached the physical size limit, researchers opened up a new direction of "More than Moore", and 3D packaging is one of the important implementation paths. 3D packaging, also referred to as stacked chip packaging, encompasses the advanced technology of vertically stacking two or more chiplets within a package while maintaining its original dimensions. Unrestricted by lattice mismatches or process compatibility issues, 3D packaging allows various layers (e.g., transmitter, 2D materials sensors, memory, and Si CMOS) to be stacked vertically to provide additional functionality [223]. Most sensor chips currently use rigid substrates, limiting their use in many applications such as intelligent wear, health detection, and haptic sensing [223]. In contrast, soft and stretchable sensors based on 2D materials can solve these problems and provide disruptive solutions for future flexible sensor chips. With the continuous advancement of 2D material analog ICs, the development of fully flexible sensor chips based on 2D materials is expected to progress rapidly. Apart from that, the explosive development in artificial intelligence has led to an increasingly close connection between sensor chips and artificial neural network algorithms. However, a large amount of data transfer and the lack of computing resources of traditional computer architecture led to a slower overall system speed and massive power consumption. The incorporation of sensors and neural network algorithms for signal preprocessing at the front-end is anticipated to decrease both hardware and software components, thereby simplifying and reducing the cost of the system.

According to the working mechanism of sensors, sensors can be generally divided into physical, chemical, and biological types. This section summarizes the latest research progress of sensor chips based on 2D materials, discusses physical chemical and biological sensors in terms of sensitive materials, sensing performance and scale, and provides an outlook on their key technologies and application prospects.

### Physical Sensors

Physical sensors are specifically designed to detect variations in the physical properties of a substance, including but not limited to temperature, pressure, strain, and light. The 2D material sensors not only outperform traditional physical sensors in some performance but also align with the trend of miniaturization and integration. Here, we summarize four representative applications of 2D materials physical sensors and chips, and compare their sensing performance in Table 2.

**Table 2:** The performance of the physical sensors based on 2D materials

Type	Materials	Sensitivity	Time	Range	Integration level	References
Temperature sensor	Graphene	–	–	283–303 K	Device	[261]
Temperature sensor	MoS_2_	TCR: ∼1 − 2% K^−1^	∼36 μs	27–85 °C	4 × 4 array	[267]
Thermocouple	Graphene	ΔS: 39 μV K^−1^	–	–	On-chip	[262]
Temperature sensor	Graphene	|TCR|: ∼ 1% K^−1^	∼30 ms	6.6–300 K	On-chip	[265]
Pressure sensor	MoS_2_	Δ*R*/*R*0: 0.011 kPa^−1^	180 ms	1 − 120 kPa	8 × 8 array	[270]
Pressure sensor	MoS_2_	1.8 MPa^−1^ (< 500 kPa)	25/27 ms	70 Pa to 5 MPa	20 × 20 array	[271]
Pressure sensor	Graphene	47.8 aF Pa^−1^ mm^−2^	–	0 − 100 kPa	Sensor chip	[272]
Pressure sensor	Graphene	5.32 × 10^−4^ kPa^−1^	–	10 − 100 kPa	MEMS chip	[43]
Pressure sensor	Graphene	5.51 × 10^−5^ kPa^−1^	–	0 − 20 MPa	MEMS chip	[273]
Strain sensor	Graphene	-	~ 4/7 ms	0 − 1.6%	8 × 8 array	[276]
Photodetector	MoS_2_	119.16 A W^−1^	44/41 ms	405, 532, 638 nm	8 × 8 array	[279]
Photodetector	PtTe_2_/graphene	∼0.52 A W^−1^	∼8.4 μs	405–1850 nm	wafer-scale	[281]
Photodetector	MoS_2_	5.2 × 10^4^ A W^−1^	50 ms	638, 532, 405, and 852 nm	wafer-scale	[282]

#### Temperature Sensors

**Fig. 15:**
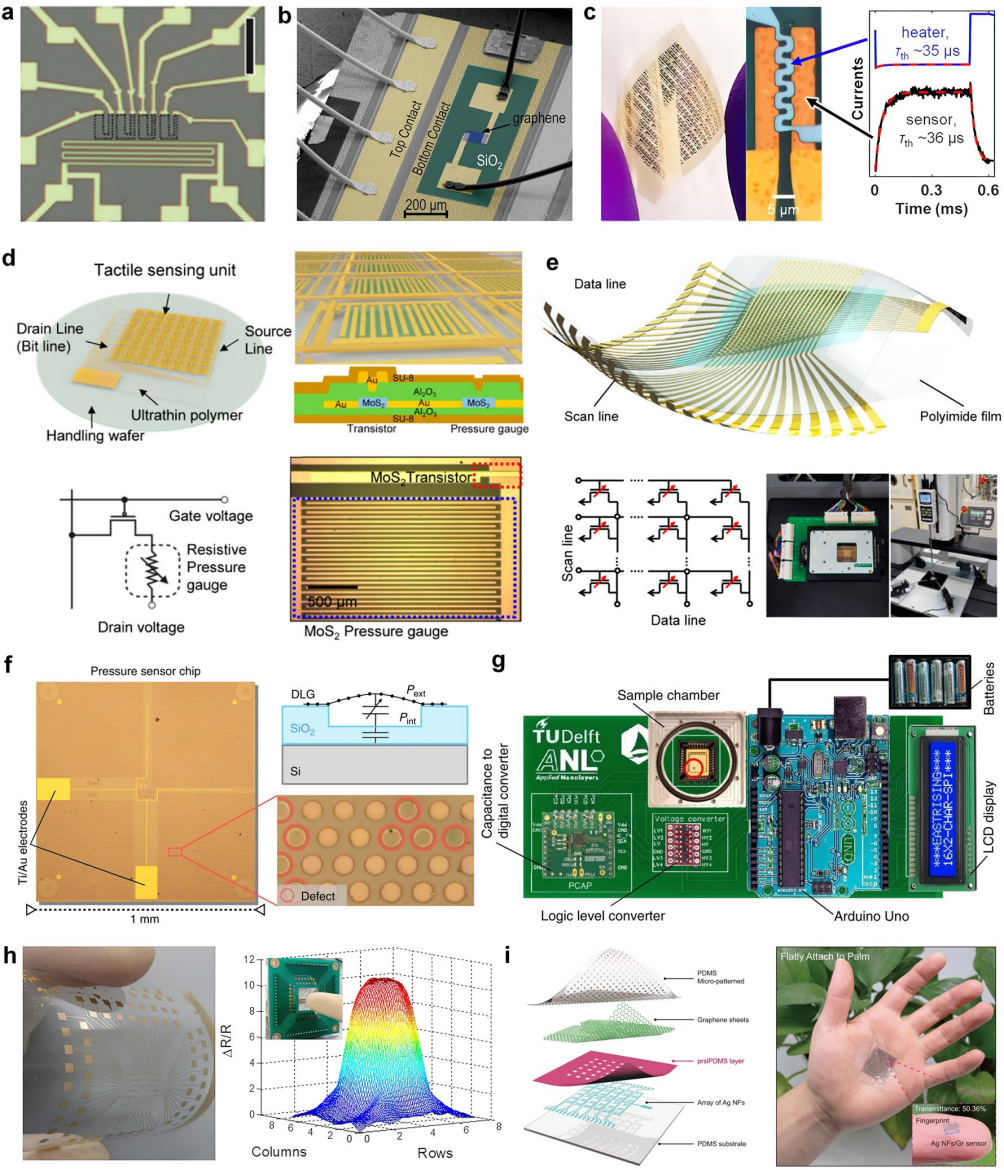
Temperature, pressure, and strain sensors based on 2D materials. **a** Optical microscope image of single-material graphene thermocouples showing the measurement configuration. Reproduced with permission [225]. Copyright (2020), Wiley–VCH. **b** Scanning electron microscopy image of the on-chip integrated graphene thermometer. Reproduced with permission [227]. Copyright (2023), American Chemical Society. **c** Optical images of the flexible substrate with MoS_2_ temperature sensor arrays, and sensor response upon pulsed actuation with the heater. Reproduced with permission [228]. Copyright (2022), American Chemical Society. **d** Schematic illustration of the fully integrated active-matrix MoS_2_ tactile sensor array, circuit diagram, and optical micrograph of thin-film transistor enabled single pressure gauge unit. Reproduced with permission [229]. Copyright (2019), American Chemical Society. **e** Active-matrix pressure sensors based on air-dielectric mos_2_ transistors for wide detection ranges from footsteps to cellular motions. Reproduced with permission [230]. Copyright (2020), American Chemical Society. **f** Optical image of the sensor chip based on double-layer graphene/PMMA membranes and Ti/Au electrodes. **g** Readout circuitry PCB board and the red circle indicates the pressure sensor chip. Reproduced with permission [231]. Copyright (2020), Nature Publishing Group. **h** Optical image of strain sensors with an 8 × 8 device array and measurement of the electric response on applying finger touching. Reproduced with permission [235]. Copyright (2015), American Chemical Society. **i** Schematic illustration of the structure of pressure sensor arrays based on the photo-reticulated strain localization films, and optical photographs of sensor arrays attached on palm. Reproduced with permission [236]. Copyright (2023), Nature Publishing Group

Temperature is a ubiquitous environmental parameter, extensively employed in both military and civilian domains, such as epidemic control and equipment monitoring. Traditional discrete temperature sensors based on thermistors, thermocouples, and platinum resistors are unable to meet the growing demand for thermal management due to their inability to integrate with large-scale systems-on-a-chip, which is driving the development of temperature sensor chips. The excellent electrical properties and high thermal conductivity of 2D materials make them vital candidates for temperature sensor chips [224]. The fabrication of back cavity and suspension graphene temperature sensors can be achieved through the utilization of MEMS processing technologies, such as lithography and wet etching, enabling their potential for large-scale production. The U-shaped graphene structure with narrow and wide legs [225] was designed to achieve enhanced sensitivity, as shown in Fig. 15a. This design choice is justified by the influence of leg width on the Seebeck coefficient.

Temperature-sensitive electronic devices usually must detect temperature changes in real time to adapt their performance accordingly. For example, the temperature sensor is embedded directly into the heat source of the power IC to detect the temperature and precisely control the temperature of the power tube, thus improving the reliability of the smart power IC chip [226]. The on-chip integrated graphene temperature sensor provided a reliable assessment of active region lattice temperatures during laser operation [227], as illustrated in Fig. 15b. The temperature coefficient of resistance (TCR) is an important indicator of the sensitivity of a resistive temperature sensor. To enhance the temperature sensitivity, the use of other 2D materials with higher TCRs is also promising. One of the main sensing mechanisms for temperature sensing is touch sensing, which transmits key information about the contact object, such as monitoring changes in human skin temperature. It is necessary to burgeon flexible temperature sensor chips to fit the contact object better and to achieve functions such as accurate temperature distribution imaging. Duas et al. fabricated flexible monolayer MoS_2_ temperature sensors and arrays with high TCR (∼1 − 2%/K) [228]. The response time of this sensor is < 36 μs, realizing real-time thermal sensing on flexible substrates (Fig. 15c). With alumina encapsulation, the cycle operation is stable and can be measured for a long time. Although sensor chips based on 2D materials have many advantages, the explanation of the sensing mechanism is not clear enough, including electron–phonon coupling, thermal expansion, electron-charged particle interaction, and the Seebeck effect.

#### Pressure Sensors

Depending on the method of signal conversion, pressure sensors can be categorized as piezoresistive, capacitive, piezoelectric, or triboelectric. Despite the great progress made in conventional pressure sensors, they are still limited to the need for specific sensing ranges, low sensitivity, and these devices are not enough for wide applications in wearable devices, smart robots, and biological systems. In order to accurately sense the pressure distribution and thus achieve multi-touch, trajectory judgment, haptic sensing, and other functions, the new generation of intelligent pressure sensors is committed to the direction of flexible sensor chips with smaller single devices, larger array size and better flexibility. The atomically thin 2D materials miniaturize pressure or strain sensors in significant measure without damaging the object’s surface shape or force signal distribution, rendering them highly promising candidates for next-generation pressure sensors. The pressure sensor chip, as depicted in Fig. 15d, integrated large-area and flexible MoS_2_ tactile sensors with an active-matrix backplane circuitry [229]. The integrated thin-film transistors enable or disable individual pressure sensors depending on their switching state, thus avoiding interference between adjacent units and effectively increasing the crosstalk isolation value to 24.8 dB, showing significant commercialization potential. By combing active-matrix arrays of pressure-sensitive MoS_2_ FETs with air dielectrics and mechanoluminescent layers [230], as exhibited in Fig. 15e, the fully integrated chip tactfully achieved high sensitivity in the detectable range of pressure from 70 Pa to 5 MPa. With this air-dielectric structure, pressure-sensitive FETs will have a low hysteresis and a high transconductance to improve their reliability and response time, providing the new approach toward next-generation biomachine interface.

Despite extensive research into pressure sensors using 2D materials, the lifetime of the devices is still extremely short due to the lack of protection of the 2D materials. Hence, highly hermetic, low-cost packaging technology is necessary to achieve increased range and stability of 2D material pressure sensors. Šiškins et al. reported sensitive capacitive pressure sensors using arrays of nearly 10,000 double-layer graphene (DLG) membranes [231], as shown in Fig. 15f. Sensors are packaged in a small vacuum chamber onboard, integrated with chip-scale electronic components, and powered by batteries (Fig. 15g). After optimizing the sensor elements, the chip layout and the readout electronics, resulting in a sensor responsivity of 47.8 aF Pa^−1^ mm^−2^. Furthermore, the combination of silicon nitride [232], boron nitride and the Au/Sn eutectic bonding technology [233] can effectively protect the 2D material and improve its stability under high temperature or humid environments.

#### Strain Sensors

The work of most strain sensors relies on the piezoresistive effect, which are changes in the resistivity of semiconductors upon mechanical strain. Compared with conventional bulk materials, 2D materials undergo greater strain engineering due to their inherent mechanical strength [234]. However, a single 2D material stress sensor cannot perform the measurement task in certain application scenarios, such as pressure distribution measurements on flat or curved surfaces. A strain sensor chip composed of many stress sensor units and processing circuits arranged reasonably can achieve relevant functions and has the advantages of high resolution and high integration. The piezoresistive property of nanographene films can be modulated by the density and lateral dimensions of the quasi-continuous nanographene film [235]. This integrated array of graphene e-skin construct has the ability to measure the surface strain distribution of thin films in real time, as shown in Fig. 15h. Zhang et al. demonstrated the ultralow crosstalk sensor array using the photo-reticulated strain localization films [236]. The unique sandwich structure not only ensures that the stacked structure will not separate but also provides physical isolation for the pressure sensor even in the bent state (Fig. 15i).

#### Optical Sensors

2D materials exhibit optoelectronic properties, physical properties, and controlled quantum properties of 2D systems beyond those of conventional semiconductors due to the atomic-level limitations of the longitudinal scale. In addition, the growth, transfer and patterning processes for large-area, high-quality 2D materials have become increasingly mature [237], making them ideal for fabricating miniature high-performance optical sensor chips. The major categories of operation mechanisms in 2D photodetectors are photovoltaic effect, photoconductive effect, photogating effect, photothermoelectric effect, and bolometric effect. Although different detection mechanisms have been reported for 2D photodetectors, their performance can still be judged by the main figures-of-merit, such as photoresponsivity, external quantum efficiency, specific detectivity, response time, optical bandwidth, and so on.

**Fig. 16:**
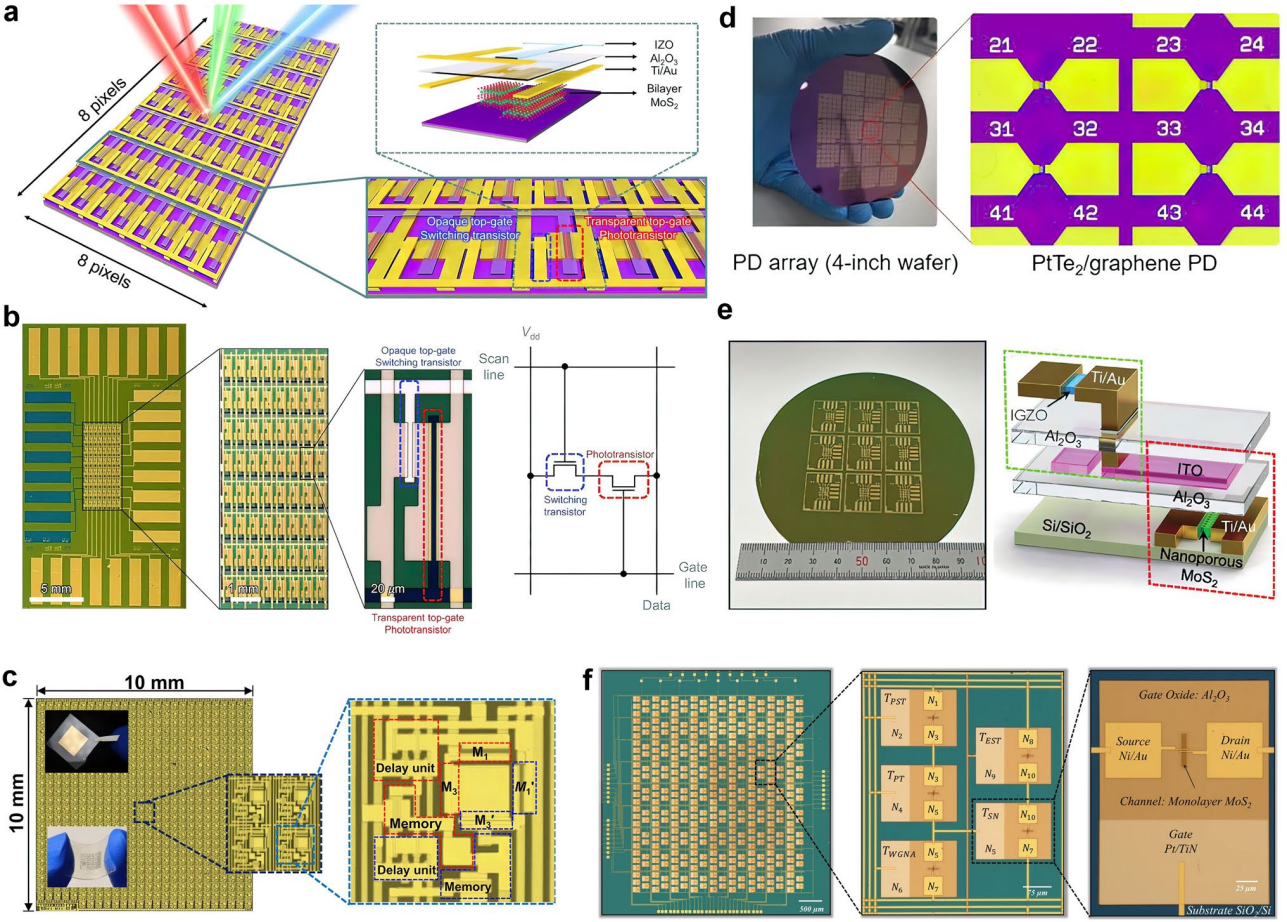
2D material-based optical sensors. **a** Schematic illustration of an 8 × 8 image sensor array based on bilayer MoS_2_ composed of opaque Ti/Au electrodes switching transistor and transparent IZO electrodes phototransistor. **b** Photograph of the 8 × 8 image sensor array based on bilayer MoS_2_ and a pixel circuit diagram of proposed image sensor array. Reproduced with permission [238]. Copyright (2020), Nature Publishing Group. **c** Optical microscopic image and zoom-in image of a fabricated artificial machine vision enhancement MoS_2_ chip on a sapphire substrate. Reproduced with permission [239]. Copyright (2022), American Association for the Advancement of Science. **d** Large-scale PtTe_2_/graphene photodetectors on a 4-inch wafer. Reproduced with permission [240]. Copyright (2022), American Chemical Society. **e** Wafer-Scale nanoporous MoS_2_ active-matrix image sensor array on 4-inch Si/SiO_2_ wafer and cross-sectional view of one pixel. Reproduced with permission [241]. Copyright (2023), Wiley–VCH. **f** Optical images of the fully integrated 8 × 8 crossbar array of crypto engines, a representative crypto engine with five MoS_2_ memtransistors, and an individual MoS_2_ memtransistor. Reproduced with permission [208]. Copyright (2022), Nature Publishing Group

At present, significant research progress has been made in photodetectors based on 2D materials. However, the current system composed of discrete devices has problems with high noise, high losses, and narrow bandwidth. Therefore, in order to adapt to the rapid development of high-capacity and high-speed optical systems, optoelectronic devices should develop toward low dimensionality, miniaturization, and integration. The incorporation of MoS_2_ switching transistors and MoS_2_ phototransistors in an active pixel image sensor array, achieved through a two-step large-area growth process, presented a promising approach [238], as shown in Fig. 16a. The transparent top-gate electrodes avoid the shading of the photosensitive area by the traditional top gate and improves the responsiveness of the photodetector (119.16 A W^−1^). The opaque top-gate switching transistors enable individual control of 64 pixels (Fig. 16b), facilitating the use of 2D material photodetector chips in next-generation image detection applications. The latest generation of intelligent photodetector chips possess the capability to perceive and process visual information within a single unit, thereby eliminating the need for redundant data transmission [239]. The monolithic vision enhancement chip, illustrated in Fig. 16c, with a resolution of 619 pixels, encompasses multiple functionalities including optical sensing, storage, and processing. Notably, the MoS_2_ analog processing circuit enables dynamic adjustment of photocurrent to align with the characteristics exhibited by human retinal neurons.

Due to the low absorption of light by single-layer 2D materials, the photoresponsivity of photodetectors based on single-layer 2D materials is often not high enough. Constructing 2D material heterojunctions is a major method to improve the overall performance of photodetectors. Figure 16d illustrates a wafer-scale photodetector based on PtTe_2_/graphene heterostructure by a directly tellurizing platinum film in a furnace [240]. This photodetector exhibited excellent photodetection performances including high D* (~ 2.58 × 10^10^ jones) and a fast response time of ∼8.4 μs. Figure 16e exhibits a wafer-scale nanoporous active pixel image sensor matrix with MoS_2_ phototransistors and indium–gallium–zinc oxide (IGZO) switching transistors [241]. Nanoporous bilayer MoS_2_ was exposed to edges that resulted in the development of subgap states, which were associated with photogating to achieve an extremely high photoresponsivity of 5.2 × 10^4^ A W^−1^. In the era of IoT, besides enhancing sensor performance, ensuring the security of sensor information is also crucial. Hence, it is necessary to develop a photoelectric sensor chip with on-chip encryption capability. The integration of crypto engines with MoS_2_-based image sensors (Fig. 16f) enabled perception, storage, and protection of information while minimizing hardware investment and average power consumption [208].

The majority of current optical sensor chips mostly detect information such as the frequency and amplitude of light. The existing technology has great limitations when the detection target is close to the radiation signal of the background. The detection of polarization can enhance the identification of the target by increasing the amount of information to seven dimensions, thereby improving its precision and accuracy. The conventional polarization detection system is intricate and exhibits a low response rate, which does not align with the current trend of high performance and integration. The emergence and continuous advancement of anisotropic 2D materials is anticipated to address the issue of polarization-sensitive detection [242]. Moreover, the polarization of isotropic 2D materials can be detected through the formation of heterojunctions or rolled-up tubular structures [243]. Further, to address the drawbacks of low yield by conventional wet-etching methods in self-assembly techniques, a meticulously controlled dry-release approach was devised on a large-scale wafer platform [244].

### Chemical Sensors

The function of chemical sensors is to convert chemical quantities, such as the composition and concentration of chemical substances, into electrical quantities. The enormous specific surface area of 2D materials enhances the intermolecular interactions, thus enabling highly sensitive detection of ultralow concentration analytes. Moreover, 2D materials have a strong surface chemistry that could be improved with chemical functionalization or defect engineering to increase their selectivity for target detection objects. Over the past decade, an increasing number of experimental strategies have integrated multifunctional 2D materials with unique analyte recognition properties into miniaturized chips for the simultaneous detection of target analytes.

#### Gas Sensors

**Fig. 17:**
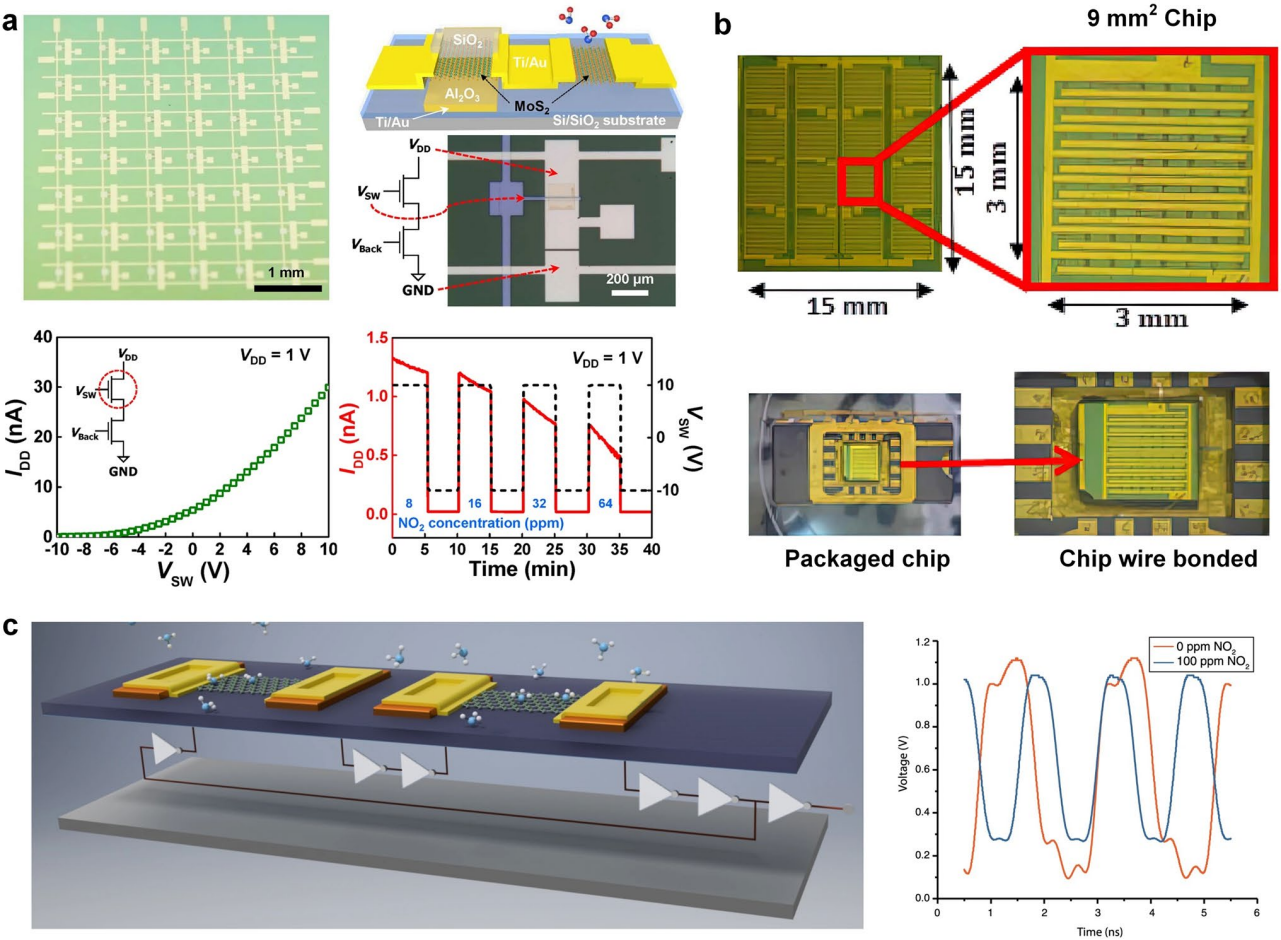
2D material-based gas sensor. **a** Morphology and performance of MoS_2_ TFTs-based gas sensor active-matrix. Reproduced with permission [246]. Copyright (2020), Nature Publishing Group. **b** Optical images of die, chips and a sensor chip wire bonded into a 16-pin DIL ceramic package. Reproduced with permission [247]. Copyright (2021), IOP Publishing Ltd. **c** Illustration of CMOS readout circuit and graphene chemiresistive sensor junctions and measured output. Reproduced with permission [248]. Copyright (2017), Nature Publishing Group

Rapid economic development brings a large amount of greenhouse gases and toxic gas emissions, which seriously threaten the natural environment and human health. The emergence of gas sensors has largely solved the problem of monitoring low concentrations of toxic and hazardous gases. Materials science and nanotechnology have contributed substantially to the advancement of gas sensor technology, striving to meet future sensor requirements. Room-temperature gas-sensitive technology based on 2D materials has received widespread attention in building high-density, low-power, and high-sensitivity gas-sensitive devices [245]. The MoS_2_ gas-sensitive thin-film transistor (TFT) manufactured over a large area exhibited excellent consistency and achieved independent control [246], as shown in Fig. 17a. Using the principle of reduced conductivity of NO_2_ adsorbed on the MoS_2_ surface, the gas sensor achieves ultrahigh sensitivity detection over a wide detection range of 1 to 256 ppm. Without the constraints of van der Waals interactions between graphene and the substrate surface, suspended graphene technology can significantly improve the gas detection sensitivity [247], as exhibited in Fig. 17b. The integration of CMOS and 2D materials is a feasible method for achieving low-cost and low-power gas sensor chips [248], as illustrated in Fig. 17c. The graphene gas sensor uses a metal through-hole connected to a CMOS readout circuit to enable the detection of NO_2_ and NH_3_, where the gas molecular interactions on the surface of the graphene sensor are read as delay-sensitive output frequencies.

#### pH Sensors

**Fig. 18:**
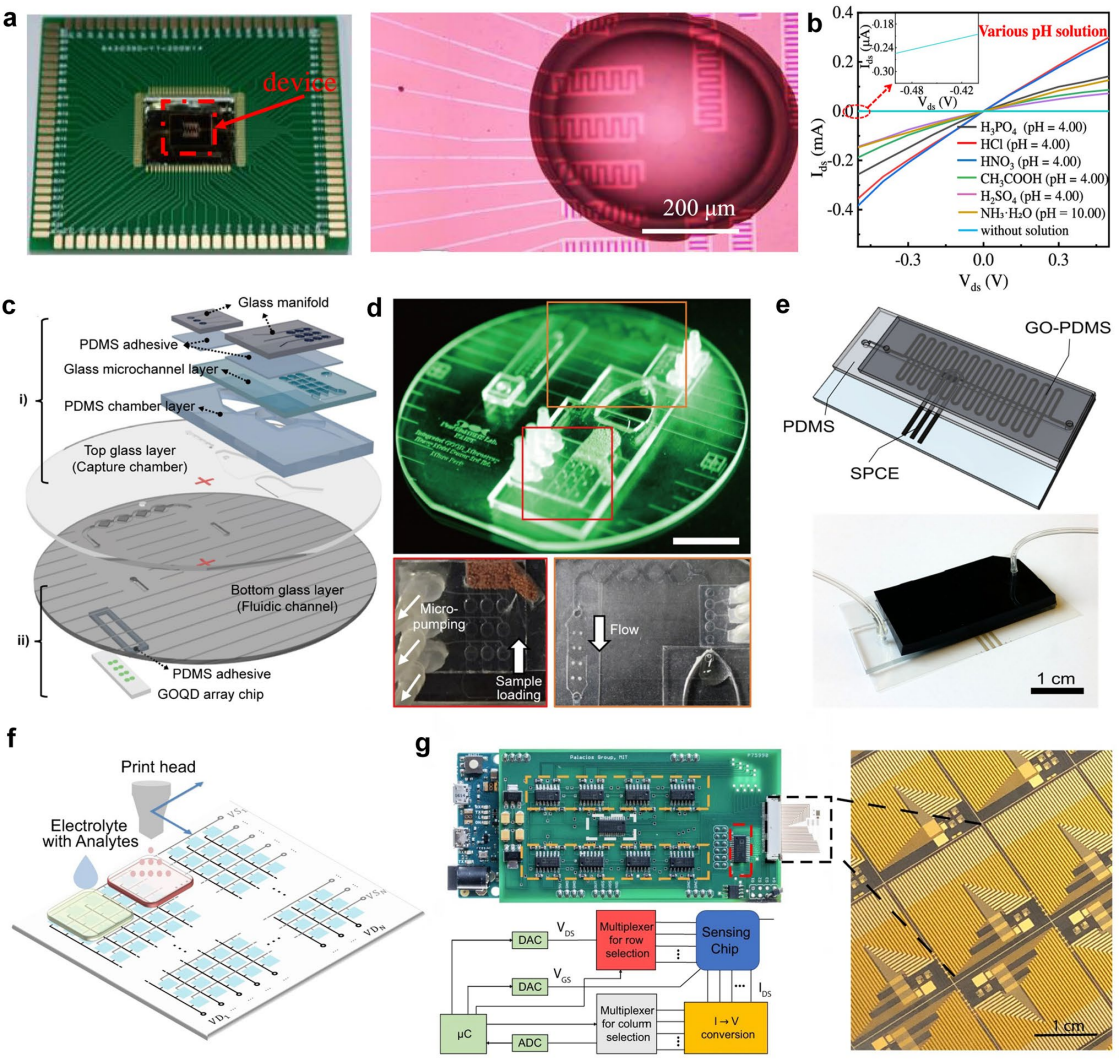
Ion sensors based on 2D materials. **a** Optical image of the integrated pH sensors based on MoS_2_ and the array devices with the added solution.** b** Selectivity of the integrated pH sensors based on MoS_2_ for detecting different pH solutions. Reproduced with permission [249]. Copyright (2021), American Chemical Society. **c** Structural illustration of a multilayered array microdevice based on the solid-phase extraction unit and graphene oxide quantum dot. **d** Optical image of the assembled microdevice. Reproduced with permission [252]. Copyright (2019), Elsevier. **e** Scheme and image of LOC device for heavy-metal preconcentration. Reproduced with permission [253]. Copyright (2017), American Chemical Society. **f** Schematic of the integrated sensor based on graphene transistor arrays. **g** Optical photograph of the measurement system and the sensor array. Reproduced with permission [254]. Copyright (2022), Nature Publishing Group

The pH sensor detects the concentration of hydrogen ions in the measured substance and converts it into a corresponding usable output signal, usually consisting of a chemical part and a signal transmission part. The classic modern commercial pH sensors measure the potential difference and monitor the hydrogen ion concentration in the measured solution utilizing a reference electrode and a target electrode. This method is highly mature in detecting hydrogen ion concentration, but its large volume and high demand for test samples greatly limit its application in certain scenarios. 2D materials are emerging candidates for pH sensor chips due to their high sensitivity properties and atomic layer thickness. Feng et al. present an all-solid pH sensor based on the monolayer MoS_2_ FET array integrated on a PCB [249], as shown in Fig. 18a. The acid solution is dropped onto the exposed monolayer MoS_2_ surface in the channel during the test and the source leakage current is observed by providing the gate voltage through an external PCB circuit. The device has an ultra-wide detection range and selectivity for different acid solutions, including the H_3_PO_4_, HCl, HNO_3_, CH_3_COOH, H_2_SO_4_, and NH_3_·H_2_O solutions (Fig. 18b). To further improve portability and integration, combining 2D materials with microfluidic structures in MEMS processes [250] or CMOS readout circuits [251] is also a valuable approach.

#### Metal Ion Sensors

Heavy metals refer to densities greater than 4.5 g cm^−3^ metals with a specific gravity greater than 5, including plumbum (Pb), mercury (Hg), cadmium (Cd), chromium (Cr), and metal like arsenic (As) with biological toxicity. These heavy metals are tough to biodegrade, but instead they can be enriched through the food chain and will inevitably pose a threat to humans and ecosystems. Heavy metals can be inactivated in the body by strong interactions with proteins and enzymes and can also accumulate in some organs of the body causing chronic toxicity. Therefore, establishing efficient heavy metal ion detection technology is of great significance for ecological environment protection, industrial wastewater treatment, and food health detection. Atomically thin 2D layered material can detect various metal ions through electrical interactions and adsorption, ushering in a new era of metal ion sensor chips. Integrated microfluidic channel is a common implementation method of liquid sensor chip [252], as illustrated in Fig. 18c. The device selectively separates target metal ions (As^3+^, Cd^2+^, and Pb^2+^) through microchannel structures, through-holes and cation exchange resins, and then automatically transfers them to array sensors for quantitative analysis (Fig. 18d). The entire process can be automated by driving pneumatic micro-pumps sequentially. Chałupniak et al. constructed a heavy metal ion sensor chip based on a novel graphene oxide–polydimethylsiloxane composite [253], as shown in Fig. 18e. The hydrophilicity of graphite oxide and functional groups including negatively charged oxygen atoms promote the combination with metal ions, realizing lead ion detection below the limit of detection (0.5 ppb). In addition, the adsorption/desorption cycle does not lose adsorption capacity, increasing device availability and reliability. The integration of sensing units, high-speed readout electronic devices and machine learning model [254], as illustrated in Fig. 18f, facilitated the advancement of real-time high-accuracy ion sensing. The sensor platform achieves high sensitivity, reversible and real-time response to K^+^, Na^+^, and Ca^2+^ in complex solutions and uses multidimensional information to train models to enhance the functionality and accuracy of the sensing system.

### Biological Sensors

Biological sensors detect and identify chemical components in living organisms by using the properties of various biological or biological substances. The high density of active sites on the surface of 2D materials promotes interaction with target objects and facilitates immobilization with other recognition elements, such as antibodies and single-stranded DNA [255]. More notably, the defects caused by the growth of 2D materials are likely to provide more binding sites, which is expected to overcome the growth challenge and achieve faster commercialization of 2D material sensor chips. According to the types of molecular recognition elements, biological sensors are classified into nucleic acid sensors, immunological sensors, microbial sensors, and so on.

#### Nucleic Acid Sensors

**Fig. 19:**
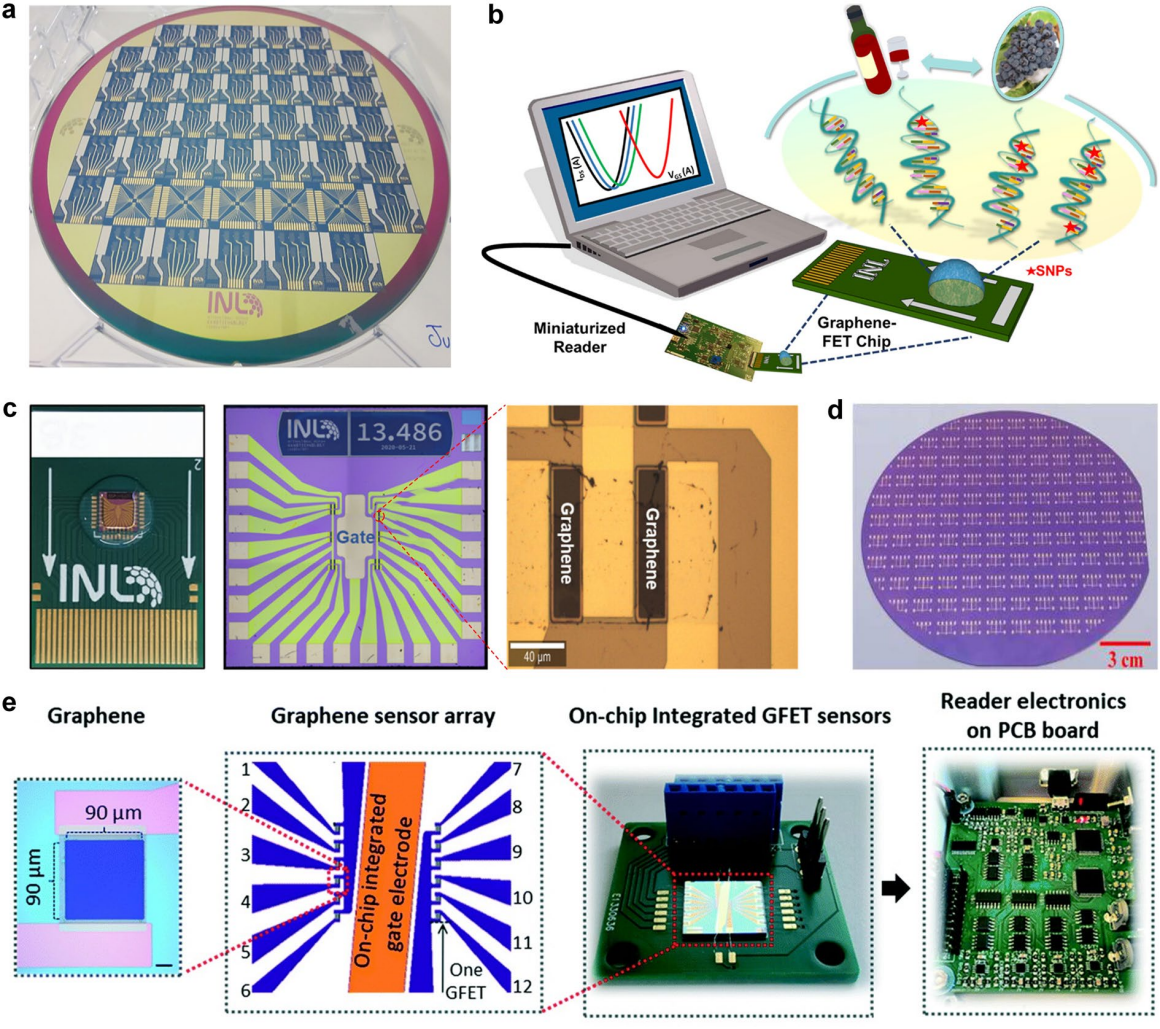
2D material-based nucleic acid sensors. **a** Photograph of the 200-mm full wafer with the graphene-based electrochemical chips for the detection of DNA hybridization. Reproduced with permission [22]. Copyright (2018), Elsevier. **b** Schematic illustration of DNA detection using a portable GFET chip. **c** Optical image of a ready-to-use GFET chip, layout, and the graphene channel on the chip. Reproduced with permission [258]. Copyright (2023), American Chemical Society. **d** Optical image of graphene field-effect transistor DNA biosensors fabricated on a 4-inch wafer. Reproduced with permission [259]. Copyright (2022), American Chemical Society. **e** Image of the graphene sensor array, the integrated on-chip sensor, and the PCB board with reader electronics for COVID-19 detection. Reproduced with permission [260]. Copyright (2022), Royal Society of Chemistry

Nucleic acids serve as genetic information carriers and the fundamental material for gene expression, possessing a plethora of unique and intriguing properties such as biocompatibility, programmability, functionalization, and molecular recognition. The correlation between nucleic acid structure and function constitutes a crucial foundation for clinical diagnosis while also holding great significance in the fields of species genetics research, medical diagnosis, and gene variation studies [256]. Nucleic acid sensors utilize nucleic acids (DNA, RNA) as recognition elements to initiate a cascade of biochemical reactions through highly specific binding with target substances, achieving high sensitivity detection and cell imaging via conformational changes or signal amplification strategies. Nucleic acids, as recognition elements for sensor arrays, offer numerous advantages such as virtually limitless sequence options composed of four nucleotide bases and different lengths, high purity and cost-effective chemical synthesis, and extensive interactions with other functional materials [257]. The ultrathin nature of the 2D material creates a large surface area in the vertical direction and quantum confinement, providing active sites for DNA or RNA immobilization and efficient detection [258]. Electrochemical biosensor chips with gold microelectrode arrays (working electrode), platinum reference and counter electrodes could be employed for the detection of DNA hybridization [22]. Wafer-scale microelectrode arrays with higher current density, faster quality transmission, and lower detection limits were manufactured through photolithography technology and then covered with DNA functionalized graphene, as shown in Fig. 19a. This sensor can detect the concentration of completely complementary DNA within the range of 5 pM to 5 nM. The single-layer graphene FET chip could also connect with a miniaturized reader to form a nucleic acid sensor for grape variety recognition [258], as exhibited in Fig. 19b. The chip adopts a unique electrode structure, with every 20 GFETs sharing a common gate electrode and every 10 GFETs having a common source electrode, realizing a dual-gate control structure (Fig. 19c). The sensor shows a wide dynamic range of 1 aM to 0.1 nM and an attomolar detection limit of ~ 0.19 aM, thus presenting a promising avenue for the development of decentralized analytical tools. In Fig. 19d, GFET modified with peptide nucleic acid shows a remarkable sequence-specific binding affinity to the target DNA and achieves an excellent detection sensitivity up to 10 fM [259]. In addition to their application in DNA detection, nucleic acid aptamer sensors are able to detect RNA viruses, specifically COVID-19. Biofunctionalization is achieved by depositing a connecting molecule (1-pyrenebutanoic acid succinimidyl ester, PBASE) on a GFET device, followed by depositing an adapter with SARS-CoV-2 spike protein [260], as depicted in Fig. 19e. The sensor adopts a self-made portable readout electronic unit, achieving high selectivity, high sensitivity, fast and stable detection.

#### Immunological Sensors

**Fig. 20:**
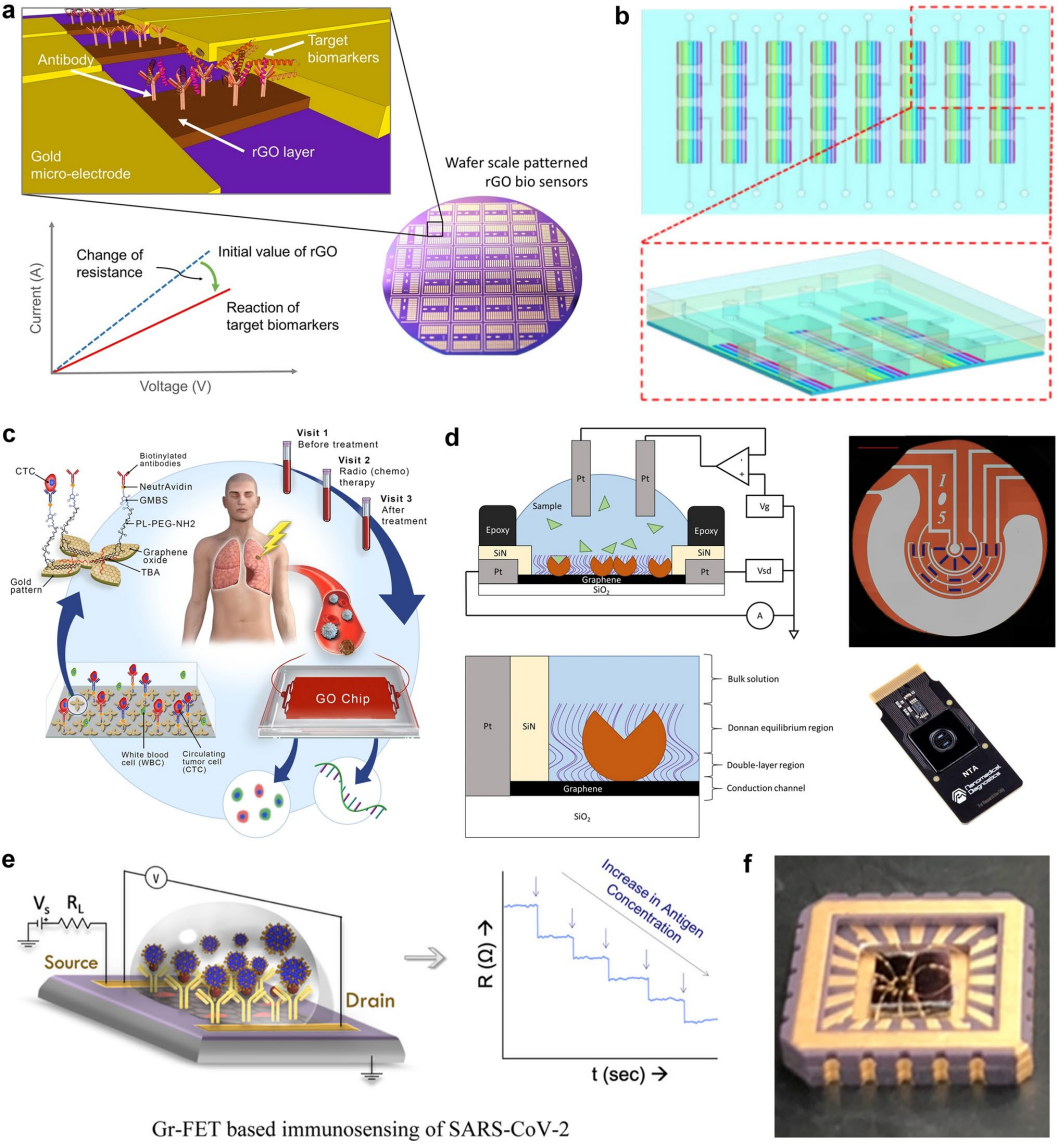
Immunological sensors based on 2D materials. **a** Schematic of wafer-scale fabricated rGO biosensors for detecting low concentration biomarkers. Reproduced with permission [261]. Copyright (2016), Nature Publishing Group. **b** Schematic structure of integrated microfluidic biosensing platform based on graphene oxide quantum dots. Reproduced with permission [262]. Copyright (2021), Nature Publishing Group.** c** Applications of the GO chip within the microfluidic chamber for measuring PD-L1 expression in circulating tumor cells. Reproduced with permission [263]. Copyright (2019), Nature Publishing Group. **d** Diagram of the foundry fabricated graphene sensor architecture, an entire sensor surface and picture of the complete biosensor. Reproduced with permission [264]. Copyright (2019), Nature Publishing Group. **e** Schematic of graphene-based field-effect transistor for ultrasensitive immunosensing. **f** Optical image of graphene-based field-effect transistor after bonding on a ceramic chip carrier. Reproduced with permission [265]. Copyright (2022), American Chemical Society

Diseases such as cancer pose a challenge for early detection and have a low cure rate in advanced stages, resulting in high mortality rates. Therefore, the development of highly specific and cost-effective detection methods holds significant research value for the timely diagnosis of complex cases. Immunological sensors employ highly specific non-covalent interactions between antibodies and antigens, including hydrogen bonds, electrostatic forces, van der Waals forces, and hydrophobic forces to capture and identify target molecules. The detected substance concentration is then converted into electrical signals. Graphene-like materials have garnered significant attention as transduction elements and support substrates in diverse biosensing technologies. Figure 20a displays a highly sensitive antibody-modified biomarker sensor chip fabricated by a wafer-scale rGO patterning method [261]. The consistency of rGO devices was substantially improved by reducing the uniformly deposited GO layers using the meniscus-dragging deposition technique and reaction ion etching. The binding of antibodies to the target biomarker induces a change in electrical resistance that is sufficiently ultrasensitive for the sensor to be utilized in Alzheimer’s disease diagnosis. Similarly, immunosensors could also leverage the high-throughput and cost-effectiveness of microfluidic chips. The integration of GO quantum dots onto a microfluidic chip enables the simultaneous detection of multiple biomarkers from at least 60 clinical samples within 40 min [262], as depicted in Fig. 20b. Besides, microfluidic chips were also used to collect whole blood from non-metastatic non-small cell lung cancer patients before, during, and after radiation or chemoradiation to monitor the effect of radiation chemotherapy on the PD-L1 expression [263], as exhibited in Fig. 20c.

These novel bio-detection techniques cannot be restricted to academic research while neglecting practical applications. Goldsmith et al. validated and produced a digital biosensor based on graphene-enabled FET chip in commercial fabrication facilities [264], as depicted in Fig. 20d. This work enables the mass production of cost-effective, low-power, portable digital biosensors that are expected to have a significant impact on healthcare, cutting-edge life science research. Moreover, the COVID-19 pandemic has also driven the rapid development of immunosensors. Shahdeo et al. designed a graphene-based FET biosensor chip to detect SARS-CoV-2 Spike S1 antigen (Fig. 20e) [265]. Immunoassay was achieved by chemically immobilizing the anti-peak S1 antibody (S1-AB) onto the surface of carboxy-functionalized graphene channels using carbodiimide. The device is bonded with gold wire on a ceramic chip carrier in order to develop a portable, miniaturized sensing platform, as shown in Fig. 20f.

#### Cell-Based Sensors

Cell-based biosensors utilize live biological cells, whether fixed or unfixed, as sensitive components of the sensor to detect and gather information on the target analyte’s characteristics. Living cells possess multiple receptors and active substances that enable them to not only respond to specific stimuli under given conditions but also quantify a wide range of analytes with less effort and cost through biosensing [266]. For cellular sensors, graphene is an intriguing material due to its high carrier mobility and remarkable sensitivity toward changes in liquid environments. Wang et al. reported a cell-based biosensor consisting of graphene and optogenetically engineered cell to pharmacodynamic evaluation of anticancer drugs [267]. The photoinduced incremental mode of transistor output current exhibits significant alterations in response to drug treatment, providing a qualitative indication of the impact of anticancer agents on cellular function. Despite some progress has been achieved in the exploration of 2D material-based cellular sensors, effectively integrating such living sensors with ICs while preserving their original biological functions remains a formidable challenge. Simultaneously, there exist problems that must be addressed to ensure the practical applicability of cell-based biosensors. Cells require specific conditions for storage, transportation, and experimentation (such as incubation conditions and nutrient availability) to ensure their stability and functionality at all time [268]. Culturing biological cells directly on a chip through biocompatible technology might be an effective solution [269], and recording intracellular voltages through precise current and voltage control by IC technology, from a new understanding and exploration of how cell sensing works.

**Fig. 21:**
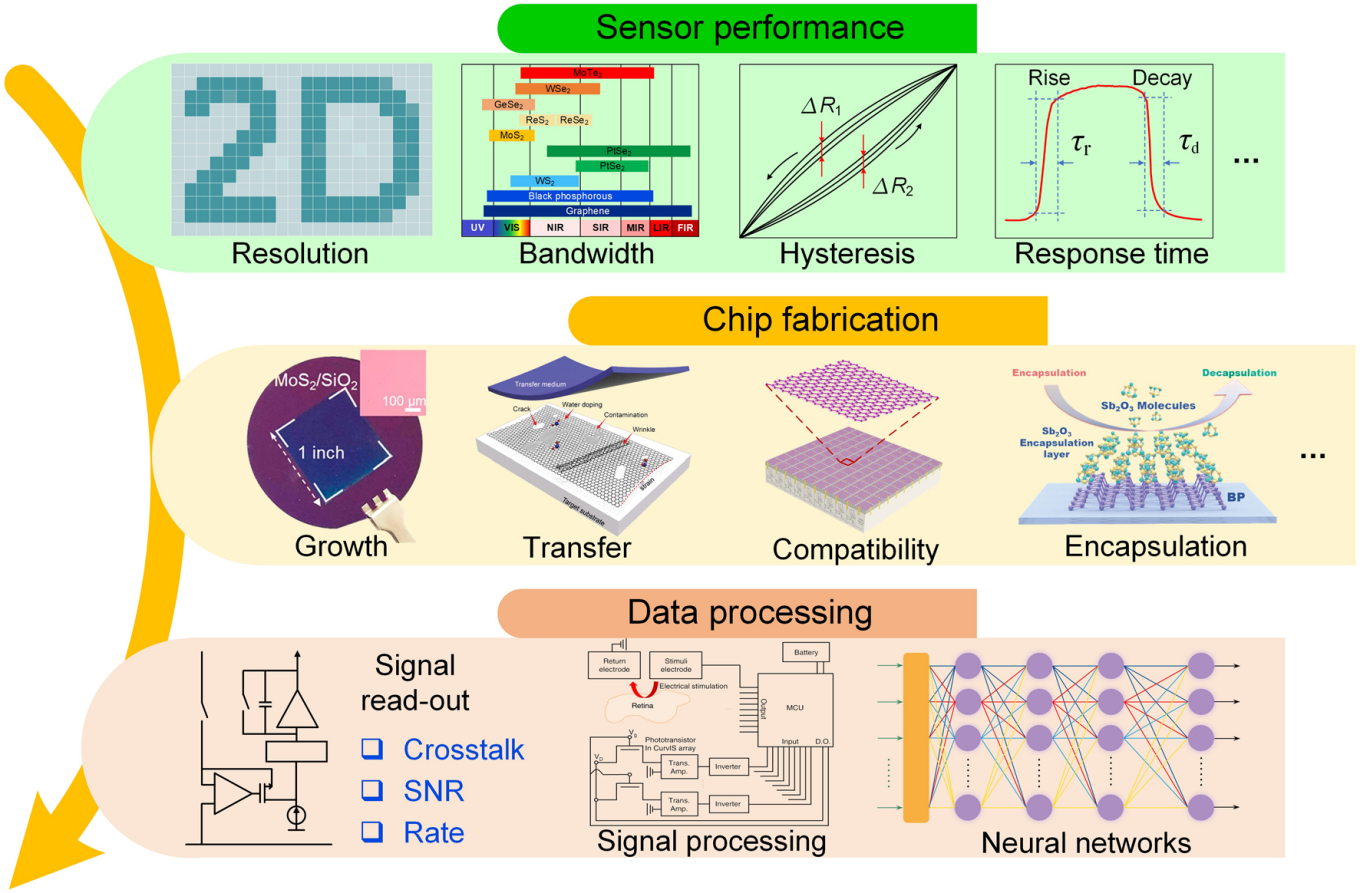
Overview of key objectives in technology of 2D materials sensors chip. Reproduced with permission [43]. Copyright (2020), American Chemical Society. Reproduced with permission [271, 273]. Copyright (2021), (2022), Wiley–VCH. Reproduced with permission [274]. Copyright (2017), Nature Publishing Group

If 2D material sensor chips are to progress toward practical application, considerations must be given to quality, performance and cost factors. In Fig. 21, the existing and possible future solutions are comprehensively discussed with respect to three aspects: sensor performance, chip fabrication, and data processing. In general, the overall quality of a sensor chip is largely determined by the performance of its individual sensors. Depending on the testing conditions, the sensor performance could be divided into basic, static and dynamic performance. Basic performance refers to the basic ability of sensors to sense changes in the environment and objects, including accuracy, stability, reliability, and bandwidth. Stability and reliability entail the ability of a device to function consistently and dependably in a dynamic environment, necessitating robust environmental adaptability from the sensor. However, 2D materials such as BP, silicene, and germanene are susceptible to oxidation and do not exist stably in air, so they are more difficult to be used in sensors. The utilization of effective passivation strategies [270], vacuum protection, and ion modification is anticipated to resolve the critical issue of severe destabilization of 2D materials in ambient air or harsh environments.

Static performance refers to the ability of a sensor to be accurately measured under static conditions, encompassing factors such as sensitivity, linearity, hysteresis, resolution, etc. Sensitivity is one of the most critical performance indicators for sensors. High sensitivity enables accurate detection of small changes in the measured physical quantity, thereby reducing system errors and biases. Dynamic performance reflects the performance of the sensor under rapidly changing conditions, mainly including response time and frequency response, etc. The evaluation of different sensor types should be based on their application scenarios and technical characteristics, with appropriate performance metrics selected to ensure optimal results. The sensor design process often involves trade-offs between sensitivity, responsiveness, bandwidth, cost, and other factors. Increasing accuracy and sensitivity may come at the expense of responsiveness or increase cost while reducing sensor size and power consumption can sacrifice signal-to-noise ratio and sensitivity. Refining the overall performance of devices through techniques such as micro-mechanical structure, strain engineering, defect engineering, and heterogeneous integration is a prevalent approach; however, each method has its own set of advantages and disadvantages. Therefore, when developing sensors, the relationship and trade-offs between the performance metrics need to be considered in relation to the actual application requirements in order to achieve the best performance in the final design. Therefore, in the development of sensors, it is crucial to consider the interplay and trade-offs among performance metrics vis-à-vis actual application requirements for optimal performance in the final design.

Fabrication is a critical technology for the advancement of 2D material sensor chips, encompassing wafer-scale growth and transfer of 2D materials, flexible preparation, packaging, and other essential processes. At present, the deposition and growth techniques of 2D materials can be applied to wafer scale, but defects and pollution do not yet meet the needs of large-scale production. The transfer technology circumvents the deleterious effects of high-temperature environments on chip synthesis for producing high-quality 2D materials. However, issues such as cracks, contamination, and wrinkles that arise during the transfer process can compromise sensor performance, thereby rendering preservation of large-area transfers a formidable challenge [271]. Moreover, sensor chips encounter challenges such as contamination, contact issues, and limited flexibility of 2D materials due to conventional lithography or deposition techniques [272]. The development of more advanced process technologies is imperative for addressing the challenges in manufacturing. The surface tension of supercritical fluid drying technology is zero, which will not cause pattern collapse, and can significantly improve the cleaning and drying ability of existing MEMS systems. The one-step lithography technology of micron scale double-layer composite structure has the advantages of simple preparation, low cost, and high machining accuracy, which can be applied to the fields of MEMS, optoelectronic chips, microfluidic chips, biochips and so on.

The output signals of sensors are often characterized by small amplitudes and complex waveforms, necessitating the utilization of signal processing technologies to enhance the signal-to-noise ratio, mitigate noise interference, optimize signal features, and ultimately enable accurate measurement of physical quantities. Due to the limitations of the 2D material growth process, ensuring uniformity across the sensor array becomes a challenge as it cannot guarantee identical performance for each individual sensor. The noise generated by the readout circuit itself and the influence of external temperature can present significant challenges in accurately capturing the sensing signal, thereby underscoring the criticality of the sensor chip’s signal readout process. With the advancement of 2D material sensor chips with record high resolution per unit area, this inevitably results in the continuous reduction of individual device size and spacing, leading to increased crosstalk between devices during the signal readout session. Enhanced filtering and coding techniques may effectively address the issue of crosstalk, albeit at the expense of heightened system complexity. Active-matrix design or the utilization of FET structure sensors, which control the switching of individual devices to solve crosstalk issues [230], can be complemented by adding isolation layers that shield electromagnetic signals. Ensuring the accuracy and readout speed of the signal while considering cost, power consumption, and area is a pivotal concern in designing signal readout circuits.

The incorporation of artificial intelligence and machine learning into system design represents a significant trend in signal processing on sensor chips. Technologies such as denoising and neural networks are being integrated closer to the sensor in order to enhance the signal processing and decision-making capabilities of sensors. As sensor chips continue to increase in scale, the discrete architecture will face a significant challenge in terms of data storage and transmission. The sensing-storage-computing architecture is a cutting-edge integration of in-deposit computing and sensing, representing one of the leading big data sensing chip architectures for the future. In general, signal processing circuits are evolving toward higher levels of integration, precision, energy efficiency and intelligence. The integration of peripheral auxiliary circuits, such as bias circuits and analog-to-digital converter circuits, into the readout circuit array can enhance chip integration while improving the sensor’s anti-interference capability. The adoption of low power consumption can effectively mitigate the energy usage of the sensor chip, aligning it more closely with the inevitable trend toward miniaturization. Excessive power consumption not only results in significant resource wastage but also elevates the temperature of the sensor chip, thereby compromising the accuracy of signal acquisition by the sensor chip. The intelligent sensing system not only satisfies the user’s requirements for sensor accuracy but also expands the range of applications for sensor chips and application scenarios.

Due to the limited progress in the development of 2D material analog and digital ICs, independent development of 2D material sensor chips without silicon and other materials is not yet feasible. Therefore, the development of 2D material sensor chips should not be a complete replacement for traditional bulk materials, but should fully utilize the unique properties of 2D materials such as interlayer excitons, topological properties, rich quantum degrees of freedom, twist angles, etc. Moreover, the feature of integrated simply should be fully utilized to explore novel physical effects by modulating the electronic structure of the 2D materials. One kind of integration is the direct stacking of different 2D materials or stacking and rotation of the same 2D material constitutes a van der Waals heterojunction, which can achieve the modulation of its energy band structure and physical properties by using the interfacial coupling between the layers. Another kind of integration is with silicon-based chips or other sensitive materials to improve the comprehensive performance of the device. Continuously research and explore toward high sensitivity, fast response speed, multi-dimensionality, wide range, high integration, and combining with neural network to break through the bottleneck of traditional sensing architecture, thus complementing and developing together with the classical silicon-based chip.

## 2D AI Chips

**Fig. 22:**
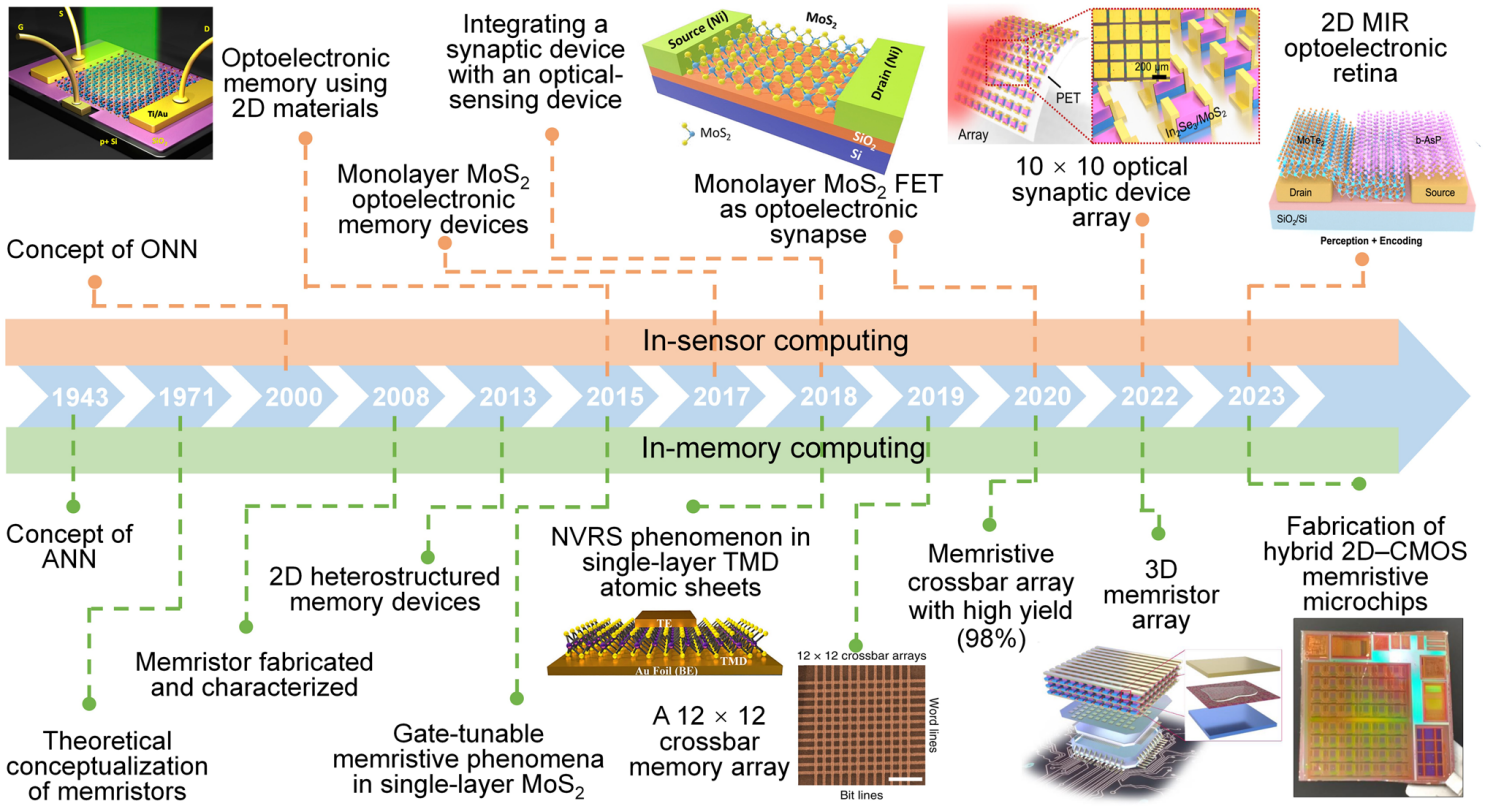
A roadmap for the evolution of memristor technology and the development of 2D materials for in-memory and in-sensor computing. The past milestones are illustrated [280–296]. Reproduced with permission [285, 288, 294]. Copyright (2015), (2018), (2022), American Chemical Society. Reproduced with permission [290, 292, 293, 295, 296]. Copyright (2019), (2020), (2022), (2023), (2023), Nature Publishing Group

In the era of big data, human society is facing a contradiction between the growing demand for AI and insufficient computing power. Modern computing systems are powered by the von Neumann architecture. The current von Neumann architecture with physically separated computing and memory units has two serious problems, memory wall and power wall [275, 276]. Processors spend most of their time and energy moving data. To solve these issues, the development of next-generation computing technologies becomes more urgent than ever. Memristors with reconfigurable history-dependent resistive switching (RS) behavior show great promise for mimicking biological synapses, which are important components of the human brain. The human brain is made up of approximately 10^12^ neurons and 10^15^ synapses [277]. By organizing these neurons and synapses, a complex information processing neural network is formed. Notably, in the human brain, information storage and calculation are in the same place, and the energy budget is within 20W [278]. Inspired by the human brain, a new computing architecture named neuromorphic computing or brain-inspired computing has been proposed [278]. Perception is the window between AI and the real world. As terminals of the Internet of Things, sensors play an indispensable role in acquiring external data, through which intelligent tasks such as inference, learning, and decision-making can be fulfilled. Although sensors have made great progress in terms of sensitivity and device size, the way to respond to external changes is mainly passive mode, that is, collecting a large amount of redundant data and transmitting it to remote computing platforms, such as cloud servers for further processing. This mode produces excessive time delay and energy consumption, ultimately reducing the time and energy efficiency of the perception system. To solve these problems, the fusion of sensor, memory, and processor is becoming a new trend in the era of Artificial Intelligence of Things (AIoT) [279]. In conclusion, as the architecture of choice for future artificial intelligence systems, the ideas of in-memory and in-sensor-computing paradigms based on non-von Neumann architectures possess broad application prospects such as neuromorphic computing and sensor-memory-processor fusion systems [279]. In this field of research, various materials are widely explored. Among them, 2D materials have attracted special attention due to their unique structures and diverse physical properties. It is necessary to emphasize that the atomic-scale thickness of 2D materials provides great advantages for realizing high-density integration and fast response processing of artificial synaptic devices. In this section, we will first present the recent process of 2D ICs for in-memory computing and in-sensor computing. The past milestones of in-memory computing and in-sensor computing and the important breakthroughs of 2D materials in this field are given in Fig. 22. Subsequently, we also go deep into the challenges and future development directions of 2D ICs for in-memory and in-sensor computing.

### 2D ICs for In-Memory Computing

With the vigorous development of AI, the traditional von Neumann computing architecture is encountering a bottleneck due to the separation of memory and computing units. In-memory computing technology, where calculations are carried out in situ within each memory unit, has been extensively studied to achieve hardware breakthroughs. Among various candidate materials, 2D materials have many unique physical properties such as atomic-scale thickness and excellent electrical properties, thus exhibiting great potential to realize artificial synapses with fast switching, low power consumption, and high integration density [297, 298]. Here, recent advances in 2D material-based synaptic devices and arrays for in-memory computing are reviewed.

#### 2D Material-Based Electrical Synaptic Devices

**Fig. 23:**
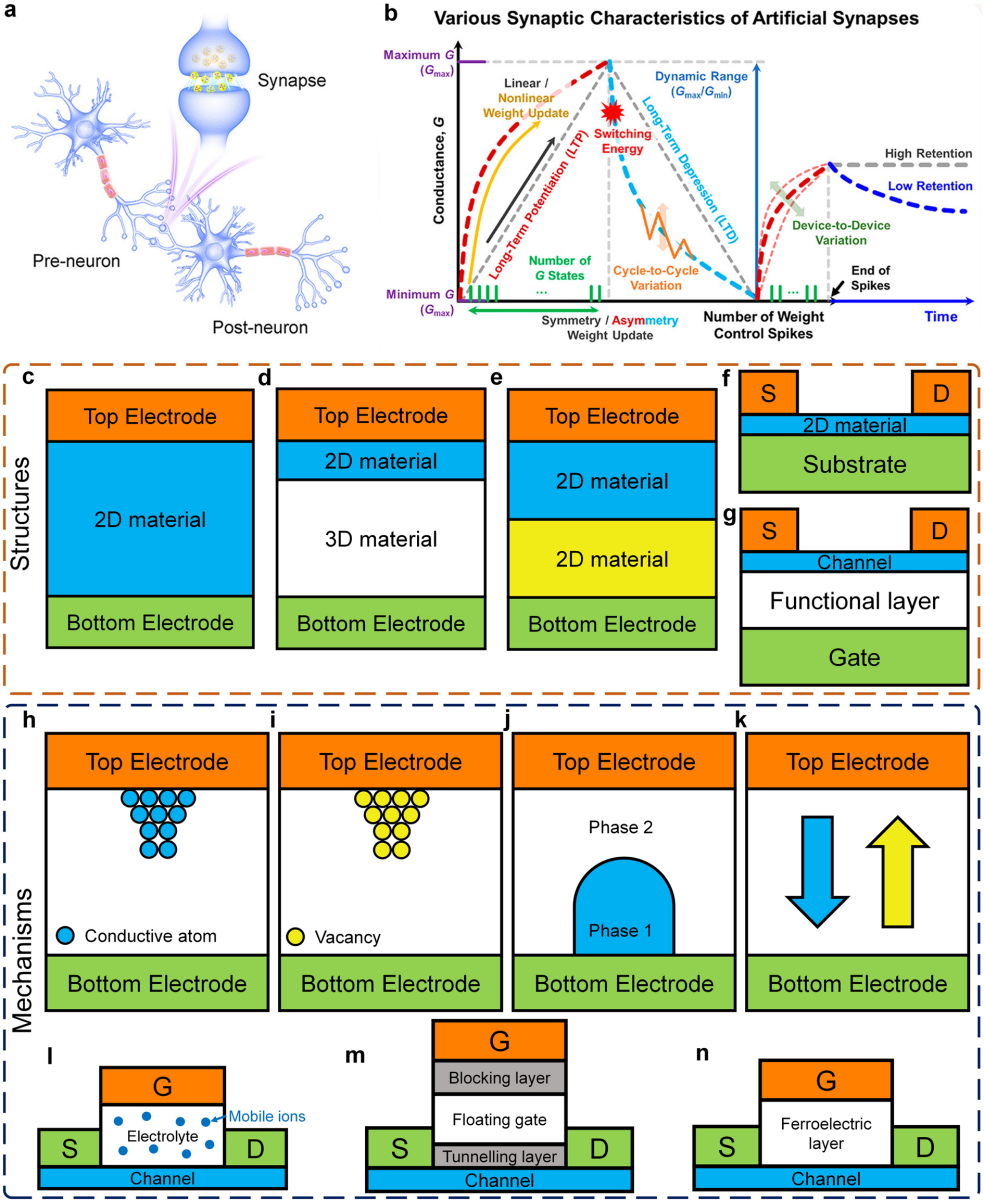
**a** Schematic diagram of biological synapses. Reproduced with permission [299]. Copyright (2021), American Chemical Society. **b** Various synaptic characteristics of artificial synapses. Reproduced with permission [300]. Copyright (2020), American Chemical Society. **c–g** Different structures of artificial synaptic devices. **h–n** Several working mechanisms of artificial synaptic devices

Efficient artificial synapses are an important cornerstone of neuromorphic computing, as synapses are crucial for interconnecting neurons and realizing brain functions, as shown in Fig. 23a [299]. To improve learning accuracy and energy efficiency of a hardware neural network (HW-NN), several synaptic characteristics are necessary. As shown in Fig. 23b, these desirable performance metrics include linear and symmetrical conductance update, large conductance maximum/minimum ratio (colloquially called “dynamic range”), sufficient number of conductance states, high endurance and retention, small cycle-to-cycle and device-to-device performance variations, and low energy consumption during updating of weight [300, 301]. It should be noted that many metrics are highly dependent on the application. For example, good endurance is required for training, while retention is critical for inference [302].

Various 2D materials have been used to prepare artificial synaptic devices, which is the first step in the construction of artificial neural networks. These materials include graphene [290, 303], MoS_2_ [304, 305], h-BN [290, 291, 306], BP [307, 308], and 2D ferroelectric materials [309–312]. 2D materials have the distinctive feature of atomic thickness. For example, graphene and h-BN have one-atom-thick layered structures, and TMDs have three-atom-thick layered structures [313]. Compared with bulk materials, 2D materials are expected to build low-power synaptic devices due to their atomically thin nature [313, 314]. Specifically, the thinnest RS devices were fabricated by reducing the thickness of the switching medium to a monolayer using 2D materials [288, 315].

As shown in Fig. 23c–n, the device structure and switching mechanism of synaptic devices based on 2D materials are summarized. Depending on the number of terminals, 2D material-based synaptic devices can be classified into two-terminal devices and three-terminal devices. According to the device geometry, the two-terminal synaptic devices can be divided into two groups, namely vertical-structure devices and lateral-structure devices. From the perspective of the type of material sandwiched between the top electrode and the bottom electrode, there are three cases of synaptic devices with vertical structure, including a single 2D material, 2D–2D, and 2D–3D heterojunctions. These 2D material-based synaptic devices have different mechanisms. In this part, we present recent progress in 2D material-based synaptic devices, including two-terminal and three-terminal synaptic devices, and consider various underlying mechanisms of synaptic devices.

Leon Chua stated: "All 2-terminal non-volatile memory devices based on resistance switching are memristors, regardless of the device material and physical operating mechanisms” [316]. The advantage of the compact two-terminal structure is its ultimate scaling capability to construct synaptic crossbar arrays with the highest possible density [317–321]. For 2D materials-based two-terminal synaptic devices, the switching mechanisms include metal filament formation [322], vacancy migration [323], phase transition [324], and ferroelectric polarization [311].

As shown in Fig. 23h, in devices based on the metal filament formation mechanism, electrochemical reaction of metal electrode dominates the RS. The anode electrode requires an active metal (such as Ag or Cu), while the cathode electrode is an inert metal (such as Pt) [325]. Memristors based on 2D materials in which the 2D material is sandwiched between top and bottom electrodes can provide opportunities to explore the limits of memristive performance at atomic-scale interelectrode distances. In particular, they help push switching voltages to lower limits, which is often an important pursuit in synaptic devices research. Xu et al. reported a vertical synapse that sandwiches two MoS_2_ monolayers between an active Cu top electrode and an inert Au bottom electrode. The RS is due to the diffusion of Cu ions through the MoS_2_ double layers to form atomic-scale filaments. Combining the atomic-scale thickness with the metal filament formation, this device exhibits the spike-timing dependent plasticity (STDP) with low switching voltages of 0.1–0.2 V, which is promising for low-power neuromorphic computing [322]. In addition to metal filament formation, another RS mechanism is based on vacancy migration, as shown in Fig. 23i. Unlike devices based on the metal filament formation mechanism, electrodes contribute less to the RS in devices based on the vacancy migration mechanism. The two electrodes are usually made of inert metals. The vacancies in the RS layer play an important role in the switching process [325]. Yan et al. demonstrated a synaptic device with the Pd/WS_2_/Pt structure. The switching mechanism of the device can be explained by the generation of sulfur and tungsten vacancies and the increase in the number of vacancies under an applied bias. The increase in the number of vacancies leads to the change of the gaps between vacancies, which promotes the electron hopping among vacancies, thus causing the modulation of the conductance. The device exhibits fast ON (OFF) switching times of 13 ns (14 ns), a low programming current of 1 µA in the ON state, and SET (RESET) energy to the femtojoule level [323]. In the field of 2D phase engineering, TMDs have attracted much attention due to their polymorphism. TMDs exist in various crystalline phases, exhibiting semiconducting, semimetallic, and metallic properties [324]. Zhang et al. developed a vertical 2H-MoTe_2_- and Mo_1−x_W_x_Te_2_-based device, in which an electric-field-induced structural transition from a 2H semiconducting to a distorted transient structure (2H_d_) and orthorhombic T_d_ conducting phase was achieved. The device exhibits repeatable RS within 10 ns between high and low resistance states. Furthermore, on/off current ratios of 10^6^ with programming currents lower than 1 μA are achieved by using an Al_2_O_3_/MoTe_2_ stack [324]. Recently, some synaptic devices based on 2D ferroelectric materials have been reported. Compared with 3D perovskite oxides, the advantage of 2D ferroelectric materials is that they can retain ferroelectric properties to the nanometer range. 2D ferroelectrics can be divided into in-plane ferroelectrics (SnSe, SnS), out-of-plane ferroelectrics (CuInP_2_S_6_), and intercorrelated ferroelectrics (In_2_Se_3_) [298]. Among these 2D ferroelectrics, α-In_2_Se_3_ shows promise in terms of high Curie temperature, high polarization, low coercive electric field, and dipole-locked in-plane and out-of-plane polarization [311]. Zhang et al. reported a synaptic device based on α-In_2_Se_3_ that utilizes the modulation of the Schottky barrier by ferroelectric polarization to achieve multiple analog conductance states.

Three-terminal synaptic devices generally utilize gate-modulated channel conductance changes in transistor structures to achieve synaptic plasticity [317]. The general structure of a three-terminal transistor-based synaptic device is illustrated, as shown in Fig. 23g. Compared with two-terminal synaptic devices, three-terminal transistor-based synaptic devices are less compact. However, due to the multi-terminal feature, three-terminal synaptic devices provide another degree of freedom not only in structural design but also in read and write operation methods for modulation of synaptic properties [317]. 2D materials-based three-terminal synaptic devices can be categorized into the electrolyte-gated transistor [326], floating-gate transistor [327], and ferroelectric-gated transistor [312], according to different operating mechanisms.

Electrolyte-gated synaptic transistors utilize the directional migration of mobile ions (such as H^+^ and metal cations) in the electrolyte dielectric layer under the action of an applied electric field to adjust the channel conductivity of the device in an electrostatic or electrochemical manner, achieving synaptic weight update [328, 329]. The main advantage of 2D materials as channel materials is the speed at which ions can redistribute in the channel following write pulses. It is much faster than 3D inorganic channel materials. Melianas et al. reported an ionic synaptic device based on proton intercalation into 2D titanium carbide (Ti_3_C_2_T_x_) MXene [326]. PVA–H_2_SO_4_ was used as the electrolyte for this device. Devices with channels consisting of six bilayers of Ti_3_C_2_T_x_/TAPA have a read–write delay of only 1 µs. Furthermore, the area normalized switching energy was estimated to be only 80 fJ µm^−2^, and this device exhibited good endurance of greater than 10^8^ write–read events. By varying the pulses applied to the gate, floating-gate transistors can effectively modulate the electrons trapped by the floating gate to achieve modulation of the channel conductance and thus the modulation of the synaptic weight. However, floating-gate transistors face some difficulties that need to be resolved. In order to solve the problem of high energy consumption caused by a large operating voltage, an extended graphene floating gate (FG) was proposed to improve gating efficiency, leading to an almost ideal subthreshold swing (77 mV dec^−1^) and reduced drain bias and switching pulses required for stable memory operation. As a result, an energy consumption of ≈5 fJ per pulse for pulse widths of 1 µs was realized [327]. Ferroelectric-gated transistors can change the polarization state of the ferroelectric material by applying a gate voltage, which can affect the channel conductance. The multi-domain polarization switching capability of ferroelectric materials enables ferroelectric-gated transistors to achieve gradual modulation of channel conductance to record synaptic weight. 2D WS_2_ possesses a thickness-dependent bandgap ranging from 1.3 (bulk) to 2.05 (monolayer) eV, enabling transistors with large current on/off ratios. Chen et al. developed an artificial synapse using ferroelectric HZO/2D WS_2_-based ferroelectric-gated transistors. The ratio between the two current states associated with different voltage sweep directions of this synaptic transistor exceeded 10^5^. Furthermore, it demonstrated a good ability to mimic various synaptic behaviors, including long-term potentiation, long-term depression, spike amplitude-dependent plasticity, and spike rate-dependent plasticity [330].

#### 2D Material-Based Electrical Synaptic Arrays

**Fig. 24:**
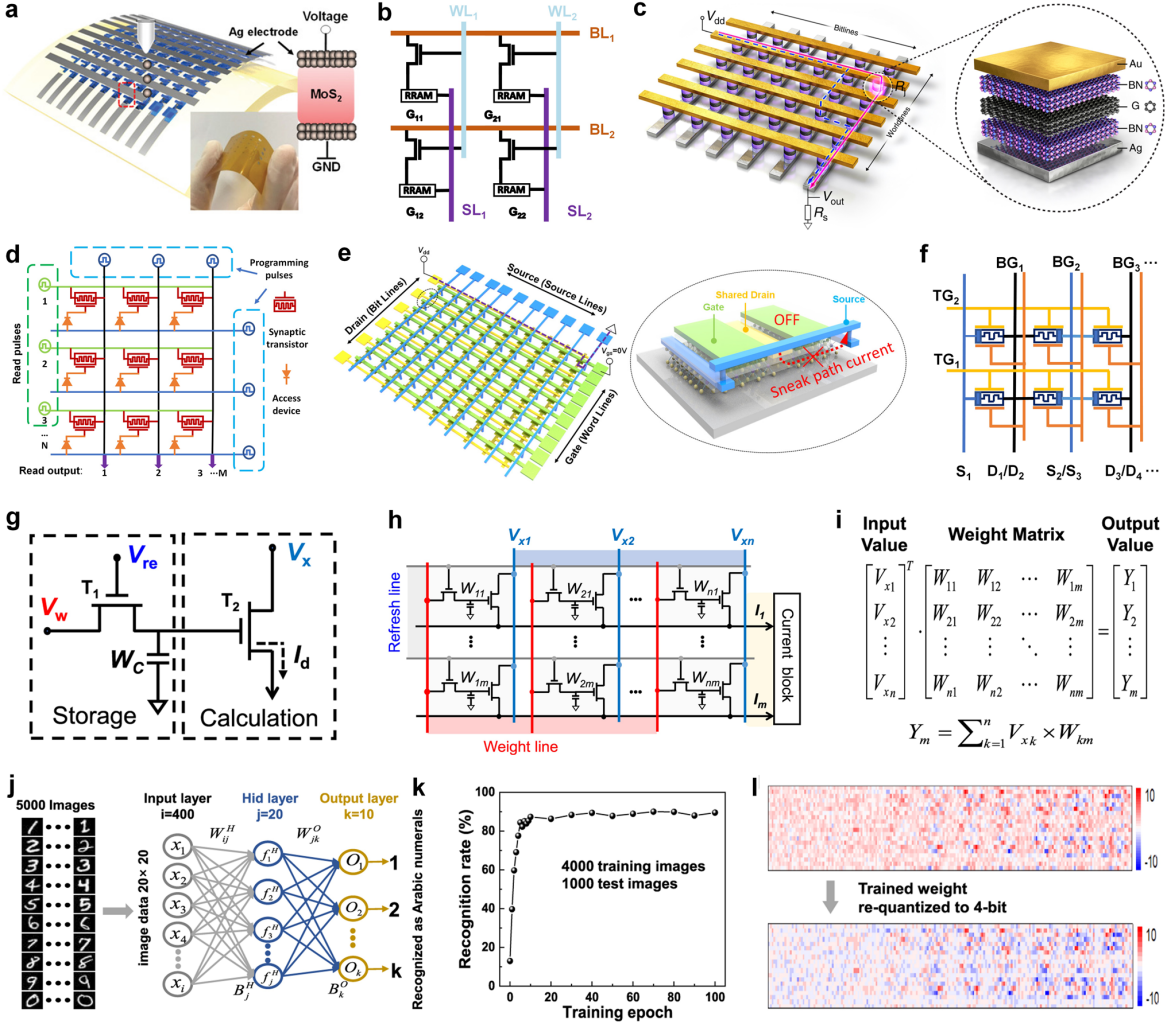
**a** Schematic plot of the fully printed Ag/MoS_2_/Ag memristor in a 4 × 4 crossbar structure on flexible polyimide substrate. Reproduced with permission [331]. Copyright (2019), Wiley–VCH. **b** An array architecture for matrix–vector multiplication with 1T1R cells to reduce sneak current. **c** Schematic picture of the van der Waals heterostructure in the crossbar memory array architecture, differing from the traditional one-selector one-resistor and complementary RS. Reproduced with permission [290]. Copyright (2019), Nature Publishing Group. **d** Circuits of a synaptic transistor-based crossbar array for matrix operations. **e** 3D schematic illustration of a 10 × 10 MoS_2_ memtransistor crossbar array. Reproduced with permission [305]. Copyright (2021), American Chemical Society. **f** Diagram of the dual-gated memtransistor crossbar array. **g** Circuit schematic of a 2T–1C cell containing storage and calculation modules. **h** Circuit diagram of the proposed 2T–1C cell array. **i** A typical diagram of a matrix convolution operation. **j** Neuromorphic network with three layers, each containing 400 input neurons, 20 hidden neurons, and 10 output neurons. **k** Recognition rate as a function of training epoch (0–100), using 4,000 images for training and 1,000 images for testing. **l** Color map showing trained weights are re-quantized to 4-bits. The size of the colormap is 20 × 200. Reproduced with permission [333]. Copyright (2021), Nature Publishing Group

In addition to advances in devices, recent advances in arrays will provide opportunities for further development in in-memory computing. Due to the inherent flexibility of 2D materials, it is promising to utilize 2D material-based synaptic devices and arrays to construct a flexible neuromorphic system. However, current memristor fabrication techniques (such as CVD) cannot be applied to flexible substrates due to the high temperature of CVD. Therefore, one solution to this problem is to prepare devices and arrays based on 2D materials using printing technology capable of low-temperature manufacturing. Feng et al. reported a fully printed 4 × 4 crossbar MoS_2_ memristor array on a flexible polyimide (PI) substrate, as shown in Fig. 24a. MoS_2_ is printed as the RS medium at the intersection point between the orthogonal rows and columns of the silver (Ag) electrode, where they are used as "bit" lines and "word" lines, respectively [331]. Although much work based on the crossbar array architecture has been demonstrated, a fundamental problem remains: with higher integration densities, sneak currents are playing an increasing role in array operations [290]. One of the solutions to the above problem is a one-transistor one-resistor (1T1R) array. Wang et al. demonstrated 3D monolithically integrated two-level stacked 1T1R memory cells, in which h-BN was used to construct RRAM and MoS_2_ transistors were utilized as stackable selector elements to reduce the sneak current path. As shown in Fig. 24b, the array architecture for matrix–vector multiplication utilizes 1T1R cells to reduce sneak current [306]. Although this 1T1R array can reduce the sneak current, it involves complicated processes. Therefore, there is a need for a novel device architecture with a self-selective memory function, along with ultralow sneak currents, high selectivity and speed, and reliability. As shown in Fig. 24c, Sun et al. demonstrated a novel cell, a self-selective van der Waals heterostructure, by constructing a vertical structure of h-BN/graphene/h-BN between Ag and gold (Au) electrodes in a crossbar array structure. 2D materials (h-BN and graphene) play a unique role in this device. On the one hand, the strong in-plane atomic bonding of high-quality h-BN layers provides a platform for ultralow off-state current and endurance against high on-state current (0.3 mA). On the other hand, the strong in-plane atomic bonding of graphene effectively prevents the diffusion of Ag filaments, which can generate a voltage across the other h-BN layer without Ag. With the assistance of graphene, this vdW heterostructure can exhibit both volatile and nonvolatile dynamics in one cell. Accordingly, this device exhibits a self-selectivity of 10^10^ with an on/off resistance ratio greater than 10^3^ and an operating time constant of tens of nanoseconds. It can be seen from this paper that the design scheme of an array architecture is closely related to the working mechanism of the devices in it. This can provide some ideas for the innovation of array architecture design [290].

Various synaptic devices have been proposed to build hardware artificial neural networks for in-memory computing. In addition to the above-mentioned arrays integrating two-terminal synaptic devices, the progress of arrays integrating three-terminal synaptic devices will be introduced below. Electrolyte-gated transistors (EGTs) are a type of synaptic transistors. Yao et al. proposed an M × N crossbar array for matrix operations, in which the GDY/MoS_2_-based EGT acts as the memory element and its channel conductance states are used as the weight update, as shown in Fig. 24d. Simulations for large digits show impressive recognition accuracy (96%). Key to this excellent performance is the high linearity and low noise of the device for current-controlled operation, which makes analog tuning very efficient [332]. Compared to 1T1R, the three-terminal unit is preferable because it is able to combine RS and selection functions into a single device without any footprint penalty. However, for devices such as floating-gate transistors and ferroelectric-gated transistors, although they are three-terminal devices, gate control still cannot provide additional selection functions to avoid crosstalk problems in adjacent cells. Feng et al. demonstrated a 10 × 10 self-selective crossbar array composed of three-terminal memtransistor unit cells with a dense cell size of 3 − 4.5 F^2^ on a continuous monolayer poly-MoS_2_ thin film. The left part of Fig. 24e schematically depicts the self-selective MoS_2_ memtransistor crossbar array architecture and its operating mechanism. A zoom-in schematic of two adjacent cells is shown in the right part of Fig. 24e, which share the same drain to reduce the number of BL to (M/2 + 1) in an M row × N column crossbar array. The multiply-and-accumulate operation was demonstrated experimentally through the memtransistor crossbar array. The MNIST handwritten recognition task was simulated, and the pattern recognition accuracy reached 96.87% [305]. Different from two-terminal memristors and three-terminal single-gated memtransistors, the array of integrated four-terminal dual-gated memtransistors has its unique advantages. Dual gating allows addressability of individual nodes in the crossbar array without the sneak current and crosstalk issues that plague traditional memristor crossbar architectures. When the two gate lines are in separate processing layers, the dual-gate design has the same footprint and scaling limitations as single-gated memtransistors. Lee et al. fabricated dual-gated MoS_2_ memtransistors on polycrystalline monolayer MoS_2_ grown by CVD. The devices used a global bottom gate and a local top gate. A dual-gated memtransistor crossbar array was constructed from these devices, as shown in Fig. 24f. In a conventional crossbar array, the energy consumption is proportional to the size of the crossbar array due to sneak current issues. This dual-gated memtransistor crossbar array is expected to minimize this additional energy consumption by minimizing sneak currents, thus providing a key advantage over conventional architectures. Another important advantage of dual-gated memtransistors is the decoupling of reading and writing operations [304].

The above describes different synaptic arrays based on non-volatile memory devices for in-memory computing. Although most non-volatile memories have the advantage of enabling multi-bit storage, they usually exhibit a stochastic nature. This leads to a loss of learning accuracy in neural network applications. The limited endurance properties of these devices are not suitable for the frequent weight update process required for in-memory computing. On the other hand, volatile memory devices with higher programming speed and excellent endurance can also perform in-memory computing. However, in volatile memories, stored information dissipates quickly and requires periodic refresh operations. Therefore, new units need to be explored and designed for in-memory computing. Wang et al. proposed a MAC circuit architecture in a 2T–1C configuration, which includes two MoS_2_ FETs and one metal–insulator–metal capacitor. As shown in Fig. 24g, the circuit schematic diagram of this 2T–1C cell includes two parts. The 1T–1C structure on the left constitutes a dynamic memory, where the MoS_2_ FET is labeled T_1_, and the MoS_2_ FET T_2_ on the right is used to complete the multiplication calculation. Due to the ultralow leakage current of the MoS_2_ FETs, a voltage with 8-level (3 bits) quantization can be stored on a capacitor with a retention time of more than 10 s, sufficient for additional complex operations. An array circuit based ona MoS_2_ 2T–1C unit cell is proposed to realize MAC operation in the circuit. The circuit diagram (Fig. 24h) corresponds to a MAC operation $Y_{m} = \mathop \sum \limits_{k = 1}^{n} V_{xk} \times W_{km}$ (Fig. 24i). A fully connected neural network (FNN) model with a 3-layer network was built for handwritten digit recognition, as shown in Fig. 24j. The relationship between recognition rate and training epoch can be seen from Fig. 24k. A color map of the trained weights after quantization to 16 levels is shown in Fig. 24l. These results show that two 2T–1C units are sufficient for a neuron to store a 4-bit quantized weight. This MoS_2_ 2T–1C circuit provides a promising research platform for in-memory computing and in situ training of neural networks [333].

Neuromorphic computing enables efficient processing of data-intensive tasks but requires numerous artificial synapses and neurons for certain functions, which leads to bulky systems and energy challenges. Achieving functionality with fewer synapses and neurons will help simplify the neuromorphic system and further facilitate the increase of integration density and computability. Compared with CMOS synapses, 2D material-based synapse devices greatly reduce hardware overhead. For neuron devices, the high firing rate and process maturity make CMOS circuits excellent candidates for artificial neurons. Given this, hybrid integration that combines the advantages of 2D synapses and CMOS neurons is attractive. Xue et al. demonstrated a hybrid neuromorphic hardware with 2D MoS_2_ synaptic arrays and CMOS neural circuitry integrated onboard [334]. The synapse- and neuron-saving hybrid hardware exhibited a competitive 98.8% accuracy and 11.4 μW single recognition energy consumption.

### 2D ICs for In-Sensor Computing

The above describes synaptic devices and arrays for energy-efficient and high-performance in-memory computing. However, systems based on this computing paradigm are still affected when interfacing with edge sensors. Because the perception and processing units are separated, this creates a lot of energy, time, and wiring overhead [335]. Therefore, many efforts have been made to build a system that integrates perception, storage, and computing functions, and this technology is called in-sensor computing or in-memory perception [335, 336]. In this review, we refer to this computing paradigm by in-sensor computing. Compared with the traditional sensing system, the sensor-memory-computing fusion system based on the in-sensor computing paradigm has the advantages of low power consumption and low data delay [337].

Vision is the most important sense of the human body because more than 80% of the acquired information is obtained through the visual system [338]. Since machine vision techniques generate large amounts of data from sensors, in-sensor computing systems that reduce unnecessary data transmission are expected to enable energy-efficient and fast visual cognitive processing [339]. Compared with the traditional bulk material system, 2D materials have inherent advantages in realizing optoelectronic synapses. First, 2D materials with atomic-level thickness enable more obvious tuning of their optoelectronic transport properties. Second, due to the unique quantum confinement effect of 2D materials, their exciton separation efficiency is very high under light conditions, which leads to a high photoelectric conversion efficiency. Third, the layer-dependent band structure properties of 2D materials enable 2D materials to have a wide range of spectral responses. Fourth, since there are no dangling bonds on the surface of 2D materials, their heterogeneous integration is relatively simple, avoiding the lattice mismatch problem of traditional material heterogeneous integration [340–342]. Here, recent reports of 2D material-based optoelectronic synaptic devices and arrays for in-sensor computing are reviewed.

#### 2D Material-based Optoelectronic Synaptic Devices

**Fig. 25:**
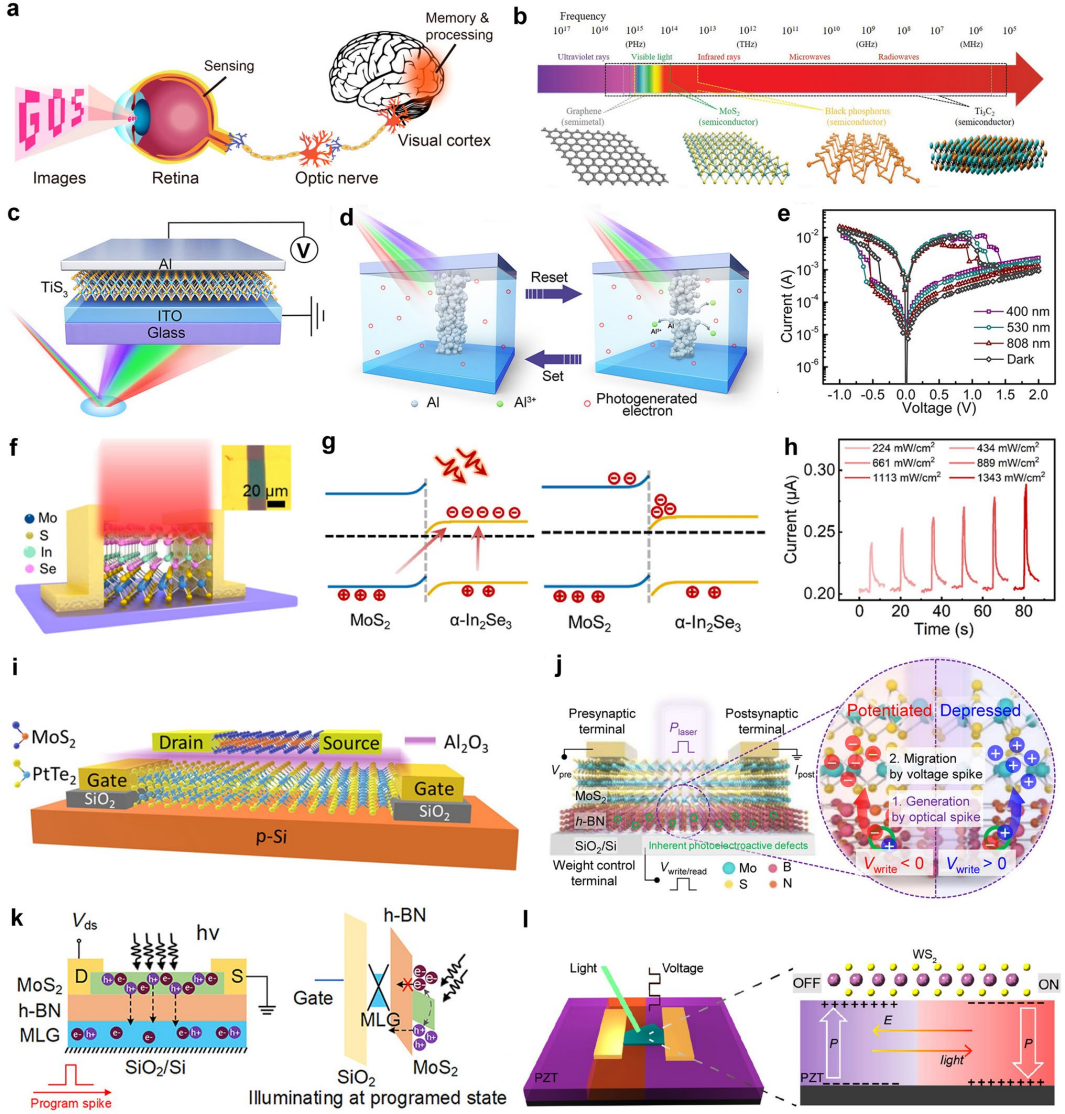
**a** Schematic of the human visual system for image sensing, memory, and processing. Reproduced with permission [349]. Copyright (2021), American Chemical Society. **b** Possible spectral ranges and atomic structures of graphene, MoS_2_, BP, and Ti_3_C_2_. Reproduced with permission [344]. Copyright (2020), Wiley–VCH. **c** Schematic diagram and **d** working mechanism of a 2D TiS_3_-based two-terminal optoelectronic synaptic device with a vertical form. **e** *I* − *V* curves collected under dark conditions and exposed to light of three different wavelengths of the TiS_3_-based optoelectronic synaptic device. Reproduced with permission [299]. Copyright (2021), American Chemical Society. **f** Schematic diagram and **g** band alignment of a In_2_Se_3_/MoS_2_ heterostructure-based two-terminal optoelectronic synaptic device with a planar form. **h** Modulation of light intensity on the performance of In_2_Se_3_/MoS_2_ devices. Reproduced with permission [294]. Copyright (2022), American Chemical Society. **i** Schematic diagram of a three-terminal optoelectronic synaptic device based on interfacial carrier capture, with PtTe_2_/Si as the gate and MoS_2_ as the channel. Reproduced with permission [343]. Copyright (2022), American Chemical Society. **j** Schematic diagram of a three-terminal optoelectronic synaptic device based on the MoS_2_/h-BN heterostructure, in which inherent defects in the material play a key role. Reproduced with permission [345]. Copyright (2021), Nature Publishing Group. **k** Schematic diagram of a floating-gate optoelectronic synaptic device based on a multilayer graphene/h-BN/MoS_2_ vertical vdW heterostructure. Reproduced with permission [346]. Copyright (2022), Wiley–VCH. **l** Schematic diagram of a ferroelectric memtransistor made of a WS_2_ channel and a ferroelectric PZT thin film for optoelectronic synaptic devices. Reproduced with permission [348]. Copyright (2020), American Chemical Society

Unlike electronic synaptic devices, which focus on achieving synaptic plasticity, optoelectronic synaptic devices combine photosensitivity and synaptic plasticity into a single device [342]. As shown in Fig. 25a, like sensory neurons in the human eye, optoelectronic synapses enable optical data sensing, storage, and processing in the same device [343]. A material with a direct bandgap smaller than the photon energy of the target detection light is an essential requirement to enhance the photoresponsivity and sensitivity of optoelectronic synapse devices [340]. The possible spectral ranges and atomic structures of graphene, MoS_2_, BP and MXene monolayer with –OH and –F surface termination and no termination (Ti_3_C_2_) are shown in Fig. 25b [344]. Although the photosensitivity of 2D materials can be utilized to construct light-sensing devices that only have the function of sensing optical data, realizing optoelectronic synapse operations still requires exploiting other specific properties of materials or developing specific technologies [343]. Different optoelectronic synaptic devices can be classified according to different criteria. Based on the device structure, the optoelectronic synaptic devices based on 2D materials can be divided into two-terminal devices and three-terminal devices. In this part, the progress of two-terminal optoelectronic synaptic devices and three-terminal optoelectronic synaptic devices based on 2D materials will be introduced successively, and the working mechanism of the devices will be considered.

In 2D material-based two-terminal optoelectronic synaptic devices, optoelectronic synaptic devices can be constructed in a vertical form or a planar form (or called a lateral form). In two-terminal devices with a vertical form, the 2D material is sandwiched between top and bottom electrodes [322]. Among different types of 2D materials, transition-metal trichalcogenides (TMTCs) have a narrow bandgap (0.2–2 eV) and respond to light from the visible to the infrared region. In addition, the large content of sulfur in TMTCs produces sulfur vacancy defects, which may be related to the RS characteristics of devices. Liu et al. demonstrated a TMTC-based optoelectronic synapse, as shown in Fig. 25c. As shown in Fig. 25d, the working mechanism of this optoelectronic synaptic device based on titanium trisulfide (TiS_3_) is the controllable formation and rupturing of the conductive aluminum filaments. The current–voltage (*I* − *V*) curves collected under dark conditions and exposed to light of three different wavelengths are shown in Fig. 25e [299]. In two-terminal devices with a planar form, the 2D material and electrodes are placed horizontally [322]. Flexible optical synaptic devices with near-infrared (NIR) sensitivity and biomimetic plasticity have great application prospects in fields such as artificial vision systems, autopilot, and robots. Compared with traditional bulk materials, 2D materials show great application prospects in the field of flexible NIR optical synapses due to their unique optical, electronic and good mechanical flexibility. Hu et al. constructed a synaptic device based on the In_2_Se_3_/MoS_2_ heterostructure, as shown in Fig. 25f. By studying the band alignment of the In_2_Se_3_/MoS_2_ heterojunction under NIR illumination (Fig. 25g), it is found that the In_2_Se_3_/MoS_2_ interface helps to separate the light-induced electron–hole pairs under NIR illumination and enhance the synaptic plasticity of this In_2_Se_3_/MoS_2_ synaptic device. Furthermore, the modulation of light intensity on the performance of In_2_Se_3_/MoS_2_ synaptic devices was investigated (Fig. 25h). The stronger the light intensity, the more photogenerated carriers are generated in the heterojunction, resulting in a larger postsynaptic current (PSC) [294].

The metal dichalcogenide, PtTe_2_, has been considered as a viable 2D material for mid-infrared (MIR) optoelectronic devices due to its transition from semiconductor (monolayer) to type-II Dirac semimetal (bulk). However, the dark current of PtTe_2_-based optoelectronic devices is high due to its low bandgap, which leads to high energy consumption of devices. Therefore, careful design of heterostructures is required during the construction of optoelectronic synapse devices as a way to exploit the infrared (IR) sensitivity of PtTe_2_ while maintaining a low dark current. Islam et al. integrated IR-sensitive PtTe_2_/Si as the gate electrode with UV–visible sensitive monolayer MoS_2_ as the channel in a transistor configuration, as depicted in Fig. 25i. The device controls the trapping and releasing of holes at the MoS_2_/Al_2_O_3_ interface through the gate voltage. More holes are trapped at the MoS_2_/Al_2_O_3_ interface at higher negative gate voltages [343]. Since the use of optical stimuli is limited to an excitatory spike pulse for most optoelectronic synapses, this limits their application to hardware neural networks. Researching a new working mechanism is an effective way to solve this problem. Oh et al. realized a unique bidirectional operation in an optoelectronic synaptic device based on the MoS_2_/h-BN heterojunction structure, as shown in Fig. 25j. This unique bidirectional operation can be explained by the ionization and neutralization of inherent defects in h-BN by co-stimulation consisting of optical and electrical spike [345]. Among various electronic devices, floating-gate memtransistors based on 2D vdW structures exhibit excellent memory behaviors, such as fast writing/erasing speed, high extinction ratio, and good endurance and retention performance. However, the integration of optoelectronic synaptic functions into floating-gate memtransistors based on 2D vdW structures remains to be achieved. To achieve this goal, Sun et al. fabricated a floating-gate optoelectronic synaptic device based on a multilayer graphene/h-BN/MoS_2_ vertical vdW heterostructure, as illustrated in Fig. 25k. Various synaptic functions, including short-term/long-term plasticity and paired-pulse facilitation, were successfully emulated by exploiting the synergistic electrical and optical modulation of photogenerated carrier tunneling through the vdW heterostructure. Furthermore, ultralow energy consumption of about 2.52 fJ per light spike was achieved due to the suppressed dark noise current (< 500 fA) after programming operation [346]. Hybrid structures comprising 2D materials and ferroelectric materials have generated considerable interest due to their attractive properties in nonvolatile memories and their potential to provide low energy consumption schemes [347]. After reports of electronic synaptic devices based on this mechanism, exploiting this mechanism to mimic complex light-controlled synaptic functions needs to be explored. Luo et al. reported a ferroelectric memtransistor made of a 2D WS_2_ channel and a ferroelectric PbZr_0.2_Ti_0.8_O_3_ (PZT) thin film for optoelectronic synaptic devices, as shown in Fig. 25l. The WS_2_ channel exhibits voltage and light-controlled memristive switching, which are related to optically and electrically modulated ferroelectric domains in the ferroelectric PZT layer. As a result, the device enables simulation of optically driven synaptic functions, including short- and long-term plasticity [348].

#### 2D Material-Based Optoelectronic Synaptic Arrays

Next, we present recent advances in optoelectronic synapse arrays for in-sensor computing, taking into account the complexity of the tasks implemented by the arrays.

**Fig. 26:**
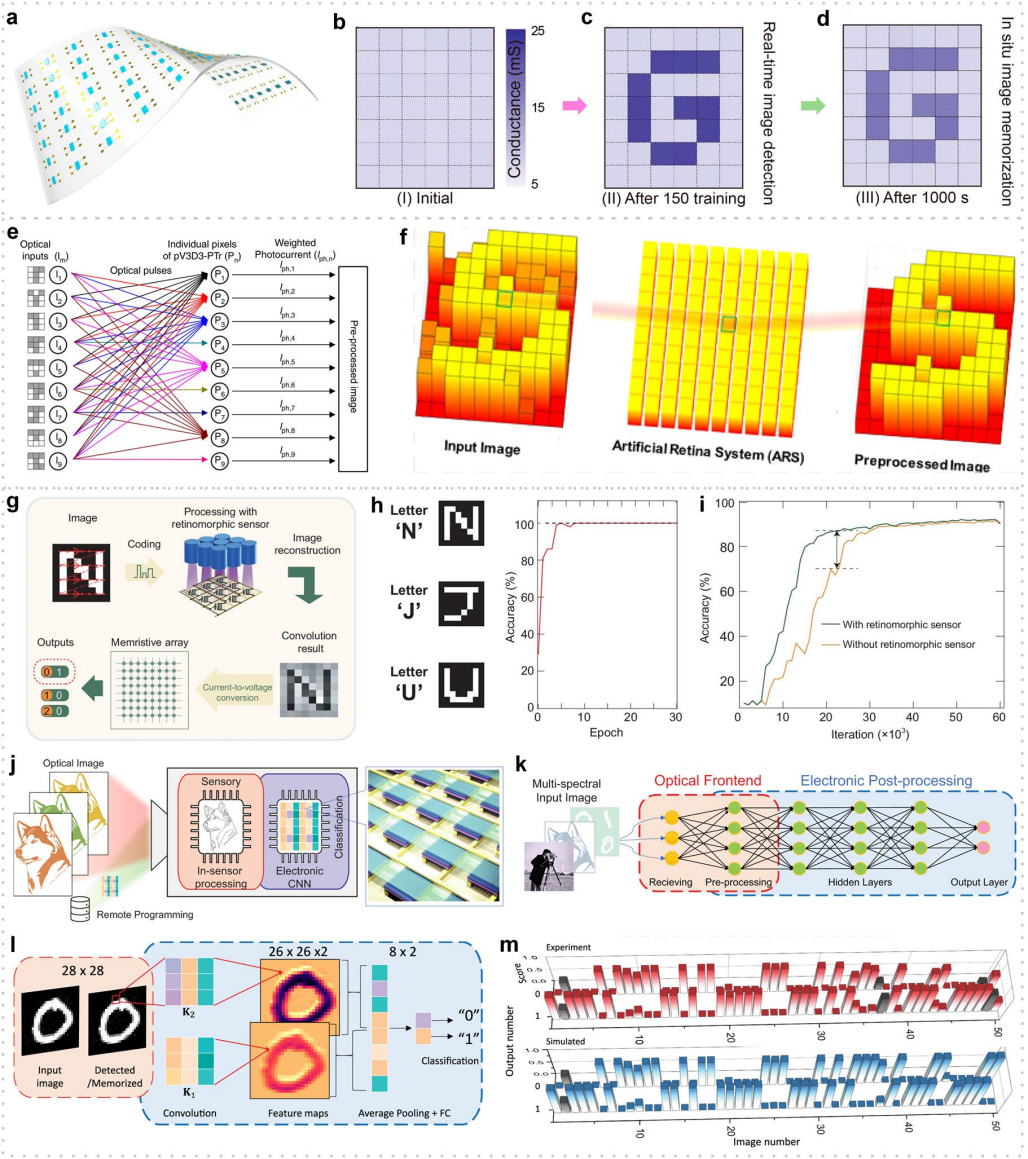
**a** Schematic of the optical synapse array fabricated on a flexible PET substrate. **b − d** Conductance map of a 7 × 6 optical synapse array. Reproduced with permission [349]. Copyright (2021), American Chemical Society. **e** Schematic diagram showing the image acquisition and neuromorphic data preprocessing by using a 3 × 3 pV3D3-PTr array. Reproduced with permission [351]. Copyright (2020), Nature Publishing Group. **f** Artificial retina with an array employing 100 (10 × 10) cells to realize the pretreatment. Reproduced with permission [338]. Copyright (2022), American Chemical Society. **g** Flow chart schematically illustrates the image sensing and processing by the retinomorphic sensor and image recognition by the memristive crossbar. **h** Image recognition by the neuromorphic vision system. **i** Comparison of recognition accuracy with and without the retinomorphic sensor. Reproduced with permission [352]. Copyright (2020), Oxford University Press. **j** bP-PPT array is capable of multispectral infrared imaging and is programmable for in-sensor computing. **k** bP-PPT array receives images in multiple wavelength bands. **l** A CNN model for classifying handwriting numbers “0” and “1” from the MNIST dataset. **m** Experimental results (top, red) for image recognition using the bP-PPT array are compared with the simulation results (bottom, blue). Each bar is the score indicating the possibility of the CNN recognizing an image in the MNIST image library. The incorrectly recognized cases are in gray. Reproduced with permission [353]. Copyright (2022), Nature Publishing Group

Building arrays capable of real-time sensing and in situ memorization of visual images is fundamental to building systems capable of in-sensor computing [350]. Hou et al. proposed a two-terminal optical synapse based on pyrenyl graphdiyne (Pyr-GDY)/graphene/PbS quantum dot (Pyr-GDY/Gr/PbS-QD) vertical heterostructure and wafer-scale (6 cm × 6 cm) Pyr-GDY was directly synthesized on a graphene surface. Excitatory and inhibitory synaptic behaviors were achieved in an optical pathway due to the strong photogating effect induced by Pyr-GDY and PbS QDs endowing the device with a bidirectional photoresponse. As shown in Fig. 26a, an optical synapse array was constructed on a flexible polyethylene terephthalate (PET) substrate to demonstrate the device's potential for flexible artificial neural networks (ANNs) in wearable electronics. In this work, a visible information sensing-memory-processing system with a 7 × 6 pixels based on the optical synapse array was constructed to perform real-time sensing, in situ memorization and distinction tasks. As shown in Fig. 26b, initially, all Pyr-GDY/Gr/PbS-QDs-based optical synapses are in the low conductance state G_0_. Since optical synapses exhibit long-term plasticity under repeated training with light signals, this system demonstrates real-time detection and in situ memory of visual images. As shown in Fig. 26c, the system perceived the letter image “G” after 150 successive optical stimuli to the corresponding synapses. The apparent conductance difference between irradiated and non-irradiated synapses could be maintained for more than 10^3^ s (Fig. 26d), suggesting in situ memorization [349]. Hu et al. also demonstrated the image learning and memory functions of an artificial vision system based on In_2_Se_3_/MoS_2_ optical synaptic device arrays and studied the imaging evolution process with the increase of illumination time and the time after illumination, respectively [294].

The biological visual system consists of the retina, optic nerve, and brain. The rods and cones neurons in the retina act as captures for visual perception, receiving light signals and converting them into physiological electrical signals. These signals can then be transmitted to the brain via the optic nerve. After the preprocessing of the retina, visual signals can be further processed by the brain to realize the functions of recognition, learning, and memory [338]. The visual information is perceived and preprocessed by the retina, so the biological retina plays an important role in image recognition [338]. Since the image pretreatment of contrast enhancement has an important impact on recognition efficiency, it is of great significance to construct an artificial retina with functions including perception and preprocessing [338]. Choi et al. achieved efficient image acquisition and neuromorphic data preprocessing by using a 3 × 3 MoS_2_-pV3D3 phototransistor (pV3D3-PTr) array, as shown in Fig. 26e [351]. Meng et al. constructed an artificial retina using an array of 100 (10 × 10) cells for preprocessing, as shown in Fig. 26f. In addition to the artificial retina, an artificial neuromorphic system was constructed for image recognition to verify the filtering ability of the proposed artificial retina. By comparing the recognition rate with and without the artificial retina, the study found that image recognition with the pretreatment of the artificial retina improves the image recognition rate and recognition efficiency. These results suggest that the construction of an artificial retina capable of preprocessing by integrating in-sensor computing optoelectronic synaptic devices is of great significance for image recognition [338].

For the realization of an artificial visual system, it is not enough to only include the perception and preprocessing functions of the visual information of the artificial retina. Furthermore, the ANN implemented on the memristive crossbar has pattern recognition capabilities similar to the visual cognition process of the human visual system [352]. So, in order to develop an artificial vision system, it is a good idea to combine the memristive crossbar used to implement the neural network with the array integrating in-sensor computing optoelectronic devices used to build the artificial retina. Wang et al. proposed a neuromorphic vision system composed of a retinomorphic sensor and a memristive crossbar. A 3 × 3 retinomorphic sensor based on a WSe_2_/h-BN/Al_2_O_3_ vdW heterostructure was fabricated to mimic the retinal function of simultaneously sensing and preprocessing an image. As shown in Fig. 26g, a neuromorphic vision system was constructed by networking the retinomorphic sensor with a large-scale Pt/Ta/HfO_2_/Ta 1T1R memristive crossbar. The constructed neuromorphic vision system is very effective in pattern recognition. As shown in Fig. 26h, the neuromorphic vision system achieved 100% recognition accuracy. Preprocessing images in the retinomorphic sensor of the neuromorphic vision system can speed up image recognition in neural networks on the memristive crossbar, which shows an advantage when processing large numbers of images. The comparison of recognition accuracy with and without the retinomorphic sensor is shown in Fig. 26i. It is worth noting that the further optimization of the manufacturing process and expansion of the retinomorphic sensor array and the memristor crossbar array are expected to significantly improve the recognition accuracy and convergence speed [352].

The artificial vision system introduced above is based on the combination of two parts, which respectively realize the functions of perceiving and preprocessing visual images and the functions of postprocessing visual images. In contrast, using the same array for both sense and pre-process images and post-process them will further reduce latency and energy consumption [353]. Lee et al. presented a multifunctional image sensor based on an array of black phosphorous programmable phototransistors (bP-PPT). The core functionality of the bP-PPT array is illustrated in Fig. 26j, where the array can detect the multispectral image in IR range as well as perform in-sensor computing and subsequent electronic in-memory computing for classification tasks. Therefore, this bP-PPT array can be utilized to realize a mixed-mode optoelectronic neural network system. As shown in Fig. 26k, in terms of the sequence of image processing, the same bP-PPT array can be used both as the optical frontend to receive and preprocess optical images, and as an electronic processor with in-memory computing to postprocess images. The 3 × 3 bP PPT array was used to demonstrate a Convolutional Neural Network (CNN) that recognizes images of handwriting numbers “0” and “1” from the MNIST data set. The CNN consists of an input layer that captures a 28 × 28-pixel image, a convolution layer, an average pooling layer followed by a fully connected (FC) layer (Fig. 26l). 100 randomly selected images of handwritten digits (48 of “0”s and 52 of “1”s) from the MNIST dataset were tested to verify the accuracy of the bP-PPT optoelectronic CNN. The bar graph in Fig. 26m compares the experimental and simulated output scores for the two labels “0” and “1” for 50 test cases, and they show excellent agreement. The study found that the CNN based on the bP-PPT array achieved an accuracy of 92%, which was comparable to the simulated result (95%). This work holds great promise for edge computing where low power consumption and low latency are required. Furthermore, the programmable photoresponsivity in the near-infrared demonstrated in this work can be extended to a broader range of infrared by heterogeneous integration of 2D materials or by varying the thickness of bP. This will allow multispectral image processing on edge devices [353].

In this section, recent research advances in electronic and optoelectronic synaptic devices and arrays based on 2D materials are reviewed. For the in-memory computing and in-sensor computing parts, we discuss devices classified according to the number of their terminal electrodes and various mechanisms. Then, we extend the discussion to arrays of integrated devices and their applications. In-memory computing and in-sensor computing based on 2D materials are emerging research fields, which are still in their infancy. Although great progress has been made in electronic and optoelectronic synapses based on 2D materials, there are still several challenges before finally turning to practical applications.

The first challenge is the wafer-scale synthesis of high-quality and uniform 2D materials. The second challenge is that the device integration process is compatible with the CMOS process. Since these two challenges are similar to those faced by 2D materials in applications such as digital and analog, they will not be discussed in detail here. The third challenge is innovation at the device level, including device structure design and working mechanism, to meet the requirements of synaptic devices for low power consumption and high reliability. For example, it is uncertain for synaptic devices based on 2D materials, although it may achieve excellent characteristics. Cycle-to-cycle and device-to-device variability will affect the weight update as well as the accuracy of image recognition. Further synaptic device innovations are one avenue to improve this variability. In addition, on the basis of realizing common synaptic functions, how to use artificial synaptic devices to simulate more complex interactions between adjacent multiple synapses requires further innovation. For synaptic devices for in-sensor computing, it is necessary to explore the appropriate device structure and working mechanism to process the perceived information. The fourth challenge appears at the circuit and array level. On the one hand, the prototype demonstration in a single device should be expanded to circuit-level and array-level operation. On the other hand, solutions need to be proposed to suppress the leakage and sneak path current in the array. For in-memory computing and in-sensor computing arrays, the peripheral circuits also need to be carefully designed to effectively control the operations in arrays. The final challenge is the reconciliation of hardware and algorithms. Ideally, algorithms could be fully implanted into neural networks built from neuromorphic devices, which feature low power consumption and high performance. However, there is currently a lack of coordination between hardware and algorithms. Therefore, ongoing research is needed to exploit the many advantages of 2D materials in in-memory and in-sensor computing. Nevertheless, all recent research results show that 2D materials have great application potential in the field of AI chips.

## 2D Quantum Devices and Circuit

**Fig. 27:**
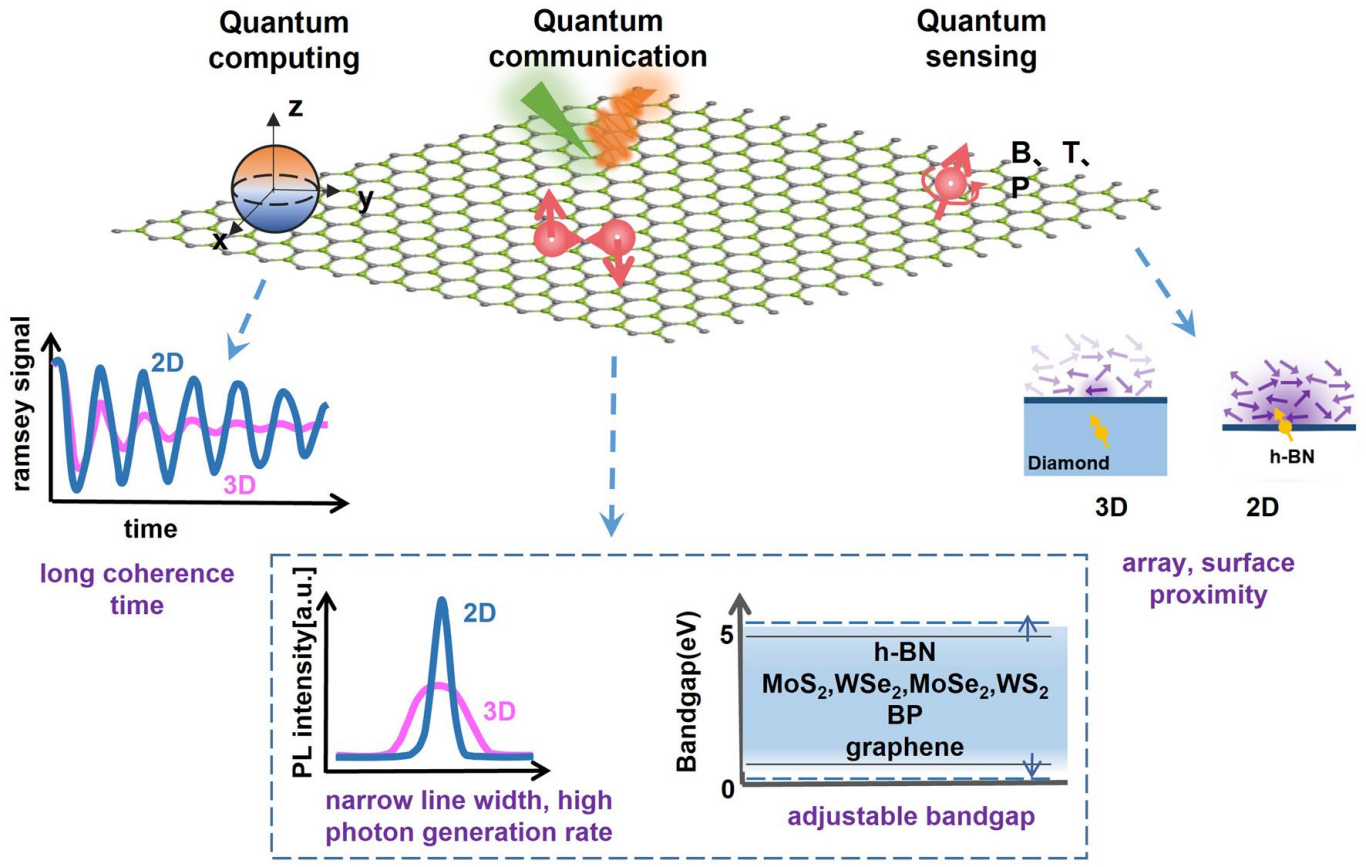
The application and advantages of 2D materials in quantum information technology, mainly focusing on quantum computing (long coherence time), quantum communication (narrow line width, high photon generation rate, adjustable bandgap) and quantum sensing (array, surface proximity)

Quantum technology is based on effects such as spin state superposition, entanglement, and coherence. It has unique advantages over classical technology in the fields of computing, communication, sensing, and storage and has become one of the main directions of development in various fields in recent years. 2D materials at the atomic scale have good flexibility, are easy to integrate into various surfaces, and have rich quantum phenomena, providing a new platform for quantum information technology, with great potential in fields such as quantum computing, quantum communication, and quantum sensing, as shown in Fig. 27. In 2D materials, electrons only move in two directions, with less interface electron scattering, which improves coherent time and has great advantages in manufacturing low-power devices. Quantum computing is based on quantum bits and realizes quantum algorithms through different quantum gate operations, which can be used for quantum simulation, quantum programming, quantum machine learning, etc. Quantum communication is mainly based on quantum emission, quantum entanglement, quantum encryption, and concealment transmission to construct a quantum communication network, which has been proven to be an absolutely secure communication method. 2D materials cover a wide range of bandgaps from zero to wide bandgaps and are easy to control. In terms of single-photon emission, they cover a large number of communication bands and have higher photon brightness. Quantum sensing is based on microscopic particle systems and quantum states, achieving high stability and sensitivity for precise measurement of physical fields. The spin defects in 2D materials used for quantum sensing are more easily arrayed for manufacturing, and the atomic-scale thickness can be used for close contact in detecting intracellular physical fields. In addition, 2D materials can undergo unique hetero-stacking and achieve multifunctional integration.

**Fig. 28:**
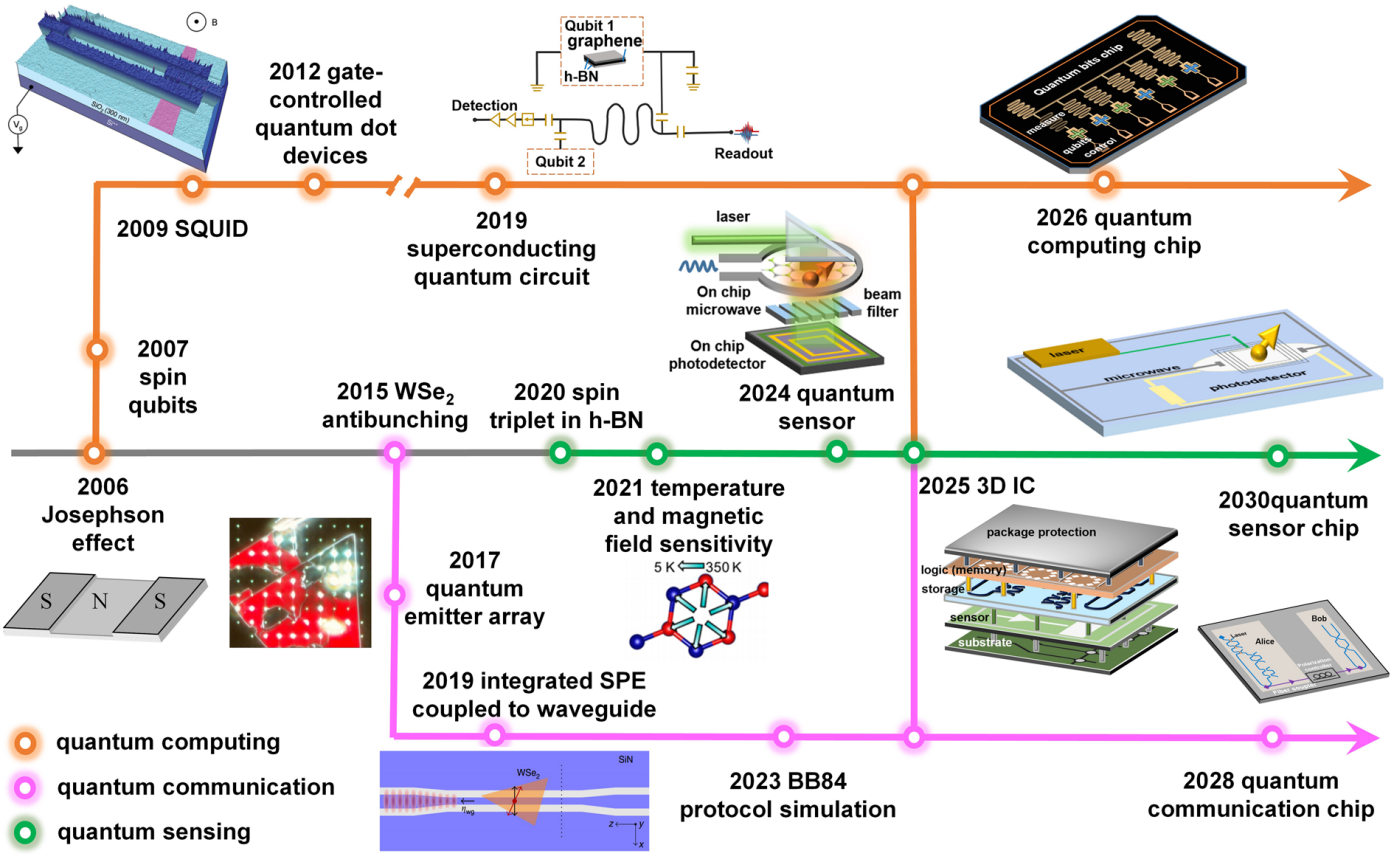
Diagram of key nodes in the development of 2D materials in quantum computing [354–358], quantum communication [359–362], and quantum sensing [363, 364]. The overall development is from phenomenon discovery to device manufacturing, and then to circuit formation. With the development of 3D integration, chips will eventually be formed. Reproduced with permission [356]. Copyright (2009), American Chemical Society. Reproduced with permission [360, 361, 364]. Copyright (2017), (2019), (2021), Nature Publishing Group

**Fig. 29:**
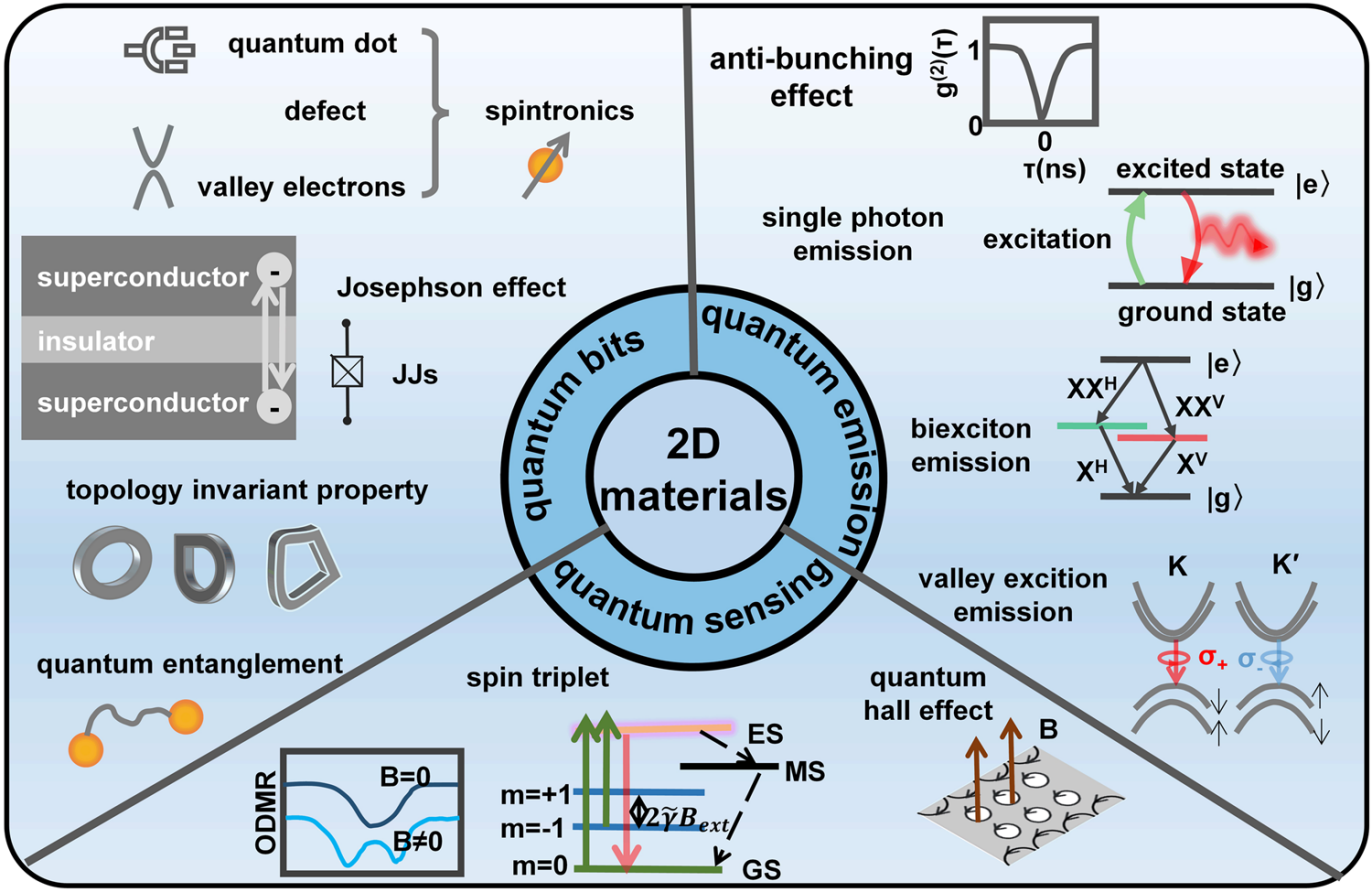
Quantum phenomena related to quantum bits, quantum emission, and quantum sensing in 2D materials

2D materials possess advantages such as high electron mobility, thermal conductivity, controllability, and quantum confinement effects. These properties enable the realization of multi-structured, high-speed, and low-power quantum devices and circuits. This section primarily focuses on the developments of 2D materials in the fields of quantum computing, quantum communication, and quantum sensing. Figures 28, 29 illustrate the key milestones in these three areas, starting from the discovery of phenomena and gradually progressing toward the formation of various devices and ultimately circuit integration. With the advancement of three-dimensional packaging technology, it is anticipated that quantum chips predominantly based on 2D materials will be formed in the future. Among them, the development of superconducting quantum computing chips based on three-dimensional materials has progressed the fastest, which will facilitate the emergence of 2D materials in chip form primarily in the field of quantum computing. However, due to the complexity of solid-state atomic spin manipulation platforms, chip-based quantum sensing is yet to materialize, which may result in a slower pace of development for quantum sensing chips.

### Quantum Effect

The ultra-thin thickness of 2D materials causes quantum limiting effect, and there are abundant quantum degrees of freedom, such as spin, energy valley, pseudospin, etc. The electrons in graphene have a linear dispersion relationship near point K, behave as massless Dirac fermion [365], and have an extremely long spin relaxation time. 2D materials have strong Rashba effect and can easily modulate electron spin by applying an electric field [366]. Energy valleys form when there are degenerate energy extrema in the conduction and valence bands. The electrons in the valley have specific momentum and spin orientation, which are called "valley degrees of freedom". The valley electronics realizes some special valley physics mechanism by regulating the energy valley state of energy valley materials [367]. Different valley polarization states can be used to encode and store information. The valley electron operation and detection were achieved in WS_2_ [368] and WSe_2_ [369], providing a new opportunity for fundamental qubit construction by valley electrons.

It is of great significance to study the quantum effects of a new 2D-layered material system to create a new prospect for the practical application of a large number of advanced functional quantum devices. 2D materials can be freely stacked to form vdW heterojunction, which can compensate for the limitation of single 2D materials according to the proximity effect, which is not available in bulk materials. The different stacking sequences and different layer spacing can change the properties of 2D layered materials, showing unique potential. By changing the relative angle between the 2D material layers, the electron band structure can be regulated and new quantum effects can be generated. The packaging technology of 2D heterojunction also provides protection for qubits and single-photon sources from environmental influences [370].

Topological quantum states have potential applications in spintronics and fault tolerant topological quantum computing. Since topological quantum states in 2D materials were predicted, topological superconductivity [371, 372], topological insulation, topological quantum phase transitions, quantum Hall effect (QHE) [373], quantum anomalous Hall effect (QAHE), quantum spin Hall effect (QSHE), and other effects have been confirmed in 2D materials. Topological superconductors (TSCs) can support Majorana states on the boundary and encode and manipulate quantum information utilizing topological protection [374], which provides an effective method for fault-tolerant quantum computing. In QHE, the magnetic field and resistance are no longer linearized, but quantized, which provides a new method for determining the fine structure constant. Under the time-reversal symmetry (TRS), the 2D topological insulators have large spin–orbit interaction, and the spin-momentum is locked, resulting in QSHE [375]. The TRS will be broken when an external strong magnetic field or material spontaneous magnetization, the former will produce QHE [376], and the latter will produce QAHE [377]. The graphene's unique linear Dirac dispersion relationship [365] makes it an ideal carrier for studying various topological quantum states. Since graphene itself is non-magnetic and the intrinsic spin–orbit effect is extremely weak, it is necessary to introduce the extrinsic Rashba spin–orbit coupling [378] and the local exchange field that destroys the TRS in order to realize the QAHE. The Coulomb interaction in graphene can lead to the instability of quantum Hall ferromagnetism. The SrTiO_3_ substrate with high-dielectric constant can achieve electrostatic shielding [379], regulate the transformation of graphene Landau level ground state into topological phase, and realize the quantum spin Hall effect in low magnetic field. The topological properties of monolayer tungsten ditelluride (WTe_2_) are easy to regulate [380], and novel quantum states can be induced by doping [381], strain, and other methods. The quantum spin Hall state of WTe_2_ can exist stably at 100 K [382], and the single layer shows the signature transmission conductivity of about e^2^ h^−1^ on each side. QSHE, in which electrons move along the edge of a material without dissipation, has broad application prospects in low-power quantum computing.

#### Quantum Bits

Quantum bits (qubits) as the basic unit of information in quantum computing have been extensively explored in 2D materials. In 2D materials, various types of qubits have been realized, including quantum dot qubits, defect spin qubits, superconducting qubits, and topological qubits. The unique energy band structure of 2D materials makes the material have multiple energy valleys, and the spin state of valley electrons can also be used to realize qubits. However, this field is still in the early stages of research and exploration. The main focus of qubits is on coherence time, decoherence time, and implementation time, among other metrics. The discovery of quantum entanglement in 2D materials will facilitate the interaction between qubits and can be utilized for information transfer and quantum gate operations.

The controllability of spin states and the ease of adjusting the band structure in quantum dots make them a candidate for qubits. By utilizing the spin states of captured charges and the Coulomb blockade phenomenon [383], quantum dot qubits can be created. Graphene has low intrinsic spin–orbit coupling and negligible hyperfine interactions [355], enabling the realization of long coherence time for quantum dot spin qubits. However, graphene also leads to slower qubit operations. TMDs have strong intrinsic spin–orbit coupling and large exciton binding energies [383], enabling rapid spin rotations, which is beneficial for fast qubit operations.

The planar structure of 2D materials is advantageous for achieving scalable and long-lived defect qubits. By controlling the energy levels of spin-active defects in the material, quantum gate operations can be realized [370]. The h-BN [384] and TMDs [370] have been shown to achieve defect spin qubits at room temperature. Defect qubits are susceptible to environmental interference, and 2D vdW heterostructures can provide protection for defect qubits. First-principles calculations have been used to design 2D material quantum defects, which will accelerate the development of spin qubits.

Superconducting qubits can be realized based on the Coulomb pairing interaction and quantum fluctuations in superconducting materials, which have the advantages of high controllability, ease of preparation, and scalability. Josephson junctions (JJs) are an important device of superconducting qubits [385], which can act as nonlinear elements, and can realize unequal energy differences between energy levels of the anharmonic oscillator. Qubits can be operated by appropriately controlling and adjusting the transitions between different energy levels. 2D materials have superconductivity and insulation, and are easy to stack into vertical vdW heterostructures [385], which can create atomically clean interfaces and facilitate the preparation of JJs, providing a new platform for superconducting qubits. However, superconducting qubits have disadvantages such as short coherence time and susceptibility to environmental interference, and further technological development is still needed.

Topological qubits are a scheme based on topologically protected qubits, including topological superconducting qubits, QHE, quantum spin oscillation, etc. When the topological structure and superconducting properties of the material interact with each other, non-Abelian anyons can appear, which can be used for qubit operations and coding. By controlling the exchange between non-Abelian anyons, qubit coupling and manipulation can be achieved. Majorana bound states, as a type of non-Abelian anyon, have been discovered in various types of 2D materials (topological insulators, topological semimetals, ferromagnetic materials, etc.) [386, 387], which provide possibilities for realizing topological qubits in 2D materials.

#### Quantum Emission

In atomically thin 2D materials, especially in monolayers, excellent luminescent properties are exhibited due to the quantum confinement of quantum wells. In 2015, the antibunching effect was observed for the first time in single-layer WSe_2_ defects [359], proving the phenomenon of quantum emission. The lack of dangling bonds in the lattice of 2D materials provides significant advantages for single-photon sources. Various quantum emission phenomena, such as single-photon emission [359], biexciton emission [388], and valley exciton emission [389], have been demonstrated in 2D materials. The large broadband of TMDs is conducive to high-speed quantum emission, and the recombination mechanism of defect emission centers has also been explained [390]. But only at low temperature, the thermal motion and phonon density in the material are relatively reduced, which reduces the interaction of electrons, holes, phonons, lattices, etc., making excitons easier to form and have longer lifetimes and Narrower line widths. This requires TMDC quantum emission to be activated at low temperature, which limits its application range. However, h-BN [391], which has strong exciton binding ability, exhibits good single-photon emission ability even at room temperature. A biexciton [388] is composed of two electrons and two holes, and it can form a strong light emission in 2D materials. Electrons and holes in 2D materials combine in the lattice valley region to form valley excitons [389], which can emit photons when excited by energy. Nonlinear optical effects can be used to generate single photons with specific frequencies and polarizations, enabling single-photon emission and manipulation. Nonclassically correlated photon pairs are observed in multilayer NbOCl_2_, which can be used to prepare quantum entangled states [392]. Quantum dots have the advantages of adjustable luminescence intensity and stable emission frequency and have been proven to be ideal materials for preparing efficient and high-fidelity single-photon sources. When an electron and a hole coincide in a quantum dot, they can recombine to produce a photon [390]. Stable single-photon emission can also be achieved by controlling the strong electron–hole coupling field in quantum dots. Researchers have found that the optical physical properties of quantum emission centers, such as transition energy, lifetime, and polarization selection, change when a magnetic field, electric field, or strain is applied [393]. This is helpful for the development of scalable quantum technology.

#### Quantum Sensing

Quantum sensing is a technique that utilizes the properties of quantum states to achieve high-sensitivity measurements. Materials used for quantum sensing require discrete and tunable energy levels. Optically addressable spin in wide-bandgap semiconductors is sensitive to magnetic fields, temperature, and pressure, and provides a good platform for quantum sensing. Recently, it has been found that h-BN with a triplet spin state can achieve high-precision detection of physical fields [363]. The initialization and readout of the spin state can be achieved through laser irradiation and microwave manipulation, and the change in the physical field is reflected in the optically detected magnetic resonance (ODMR) signal [364]. The QHE is a phenomenon in which electrons move along the boundary under the action of a magnetic field. According to this phenomenon, the sensing of the magnetic field can also be realized. The high sensitivity, precision, low noise, and non-destructiveness of quantum sensing will be beneficial for its wide application, but the technology is still in its early stages and requires continuous exploration.

### Quantum Devices

Quantum devices utilize the characteristics of quantum states such as entanglement, superposition, transmission, and environmental sensitivity to achieve various functions, with the advantages of high precision, fast efficiency, and high security. With the continuous development of fields such as quantum computing, quantum communication, and quantum sensing, quantum devices have gradually become widely researched. Common quantum devices include qubits, quantum gates, quantum key distributors, quantum relays, and quantum sensors. 2D materials have also become a research hotspot because of their special quantum phenomena and extremely small size characteristics. Currently, basic quantum devices such as JJs for superconducting qubits, gate-controlled quantum dots for semiconductor qubits, single-photon emitters, solid-state quantum sensors, etc., have been implemented on 2D materials. This subsection mainly introduces these four quantum devices. In addition, some quantum devices based on quantum effects, such as spin transistors, magnetic tunnel junctions, Fabry–Pérot quantum Hall interferometers [394], and quantum entanglement single-photon field-effect transistors [395], have also been fabricated on 2D materials. With the continuous exploration of 2D material systems, multifunctional quantum devices will be further improved.

#### Josephson Junctions

The Josephson junction (JJ) is an electronic device consisting of a superconductor–normal–superconductor (SNS) structure, where the normal layer provides a tunneling barrier that forms a weak link between the superconductors. The superconducting charge can flow through the normal layer without any resistance, exhibiting interesting superconducting properties. When an oxide layer is used as the normal layer, the oxygen atoms in it can diffuse easily due to the highly non-uniform distribution of the supercurrent, leading to a shortened device lifetime. When a ferromagnetic layer is used as the normal layer, the spin of the superconducting electrons is polarized, resulting in non-uniform phase shifts and a π-phase difference between the superconducting and ferromagnetic electrons, forming a π JJ. In an environment with a certain level of microwave radiation, a specific AC voltage is generated at the two ends of the JJ, which can be used to produce microwave detectors and superconducting quantum computing devices. The superconducting quantum interference device (SQUID) consists of a JJ and a superconducting loop, and it is a key device of ultrasensitive electromagnetic measurement systems. In superconducting circuits, JJs are used to realize two-level systems to form qubits.

**Fig. 30:**
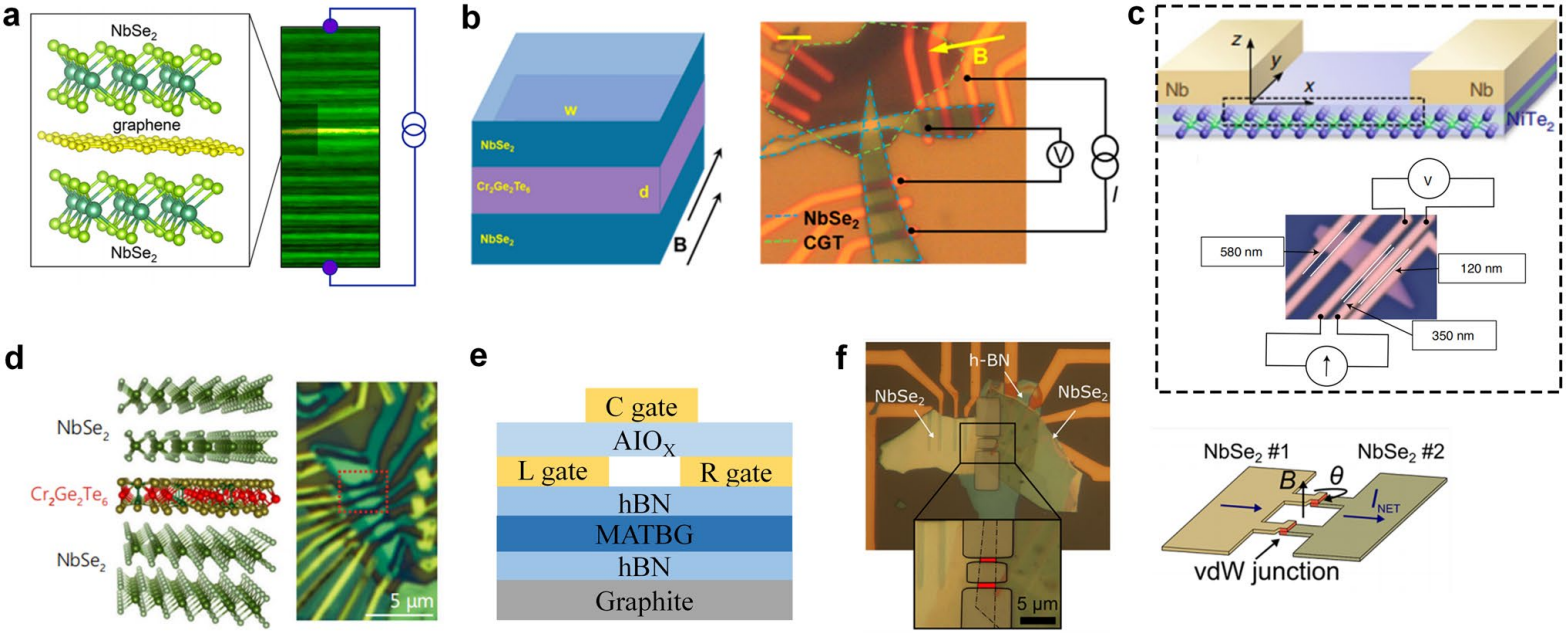
Development of JJs in 2D materials. **a** Vertically stacked NbSE_2_-graphene-NbSE_2_ JJ: Atomic structure schematic of heterostructure and scanning transmission electron microscope (STEM) image of vertical cross-section. Reproduced with permission [397]. Copyright (2017), American Chemical Society. **b** π JJs composed of the plane NbSe_2_/Cr_2_Ge_2_Te_6_/NbSe_2_. The thickness of the barrier layer is *d* and the width is w. Optical micrographs and measurement circuit diagrams are shown on the right. Reproduced with permission [398]. Copyright (2022), American Chemical Society. **c** Schematic diagram of the NiTe_2_ Josephson diode device and optical microscope image of several JJ devices formed on a single NiTe_2_ peel sheet. Reproduced with permission [399]. Copyright (2022), Nature Publishing Group. **d** Josephson coupling of NbSe_2_/Cr_2_Ge_2_Te_6_/NbSe_2_ vdW junctions. Left: Atomic diagram of a JJ. Right: Optical micrograph. Reproduced with permission [396]. Copyright (2021), Nature Publishing Group. **e** A schematic diagram of the interface of gated magic Angle graphene to achieve tunable JJs knots. The MATBG is tuned to a superconducting state utilizing a graphite base gate electrode and the left (L) and right (R) top gate electrodes, and the center (C) gate is tuned locally to a non-superconducting state. The insulated h-BN separates the gate electrodes from the MATBG, and the alumina (AlO_x_) layer isolates the top upper and lower doors [400]. **f** SQUID based on twisted NbSe_2_-NbSe_2_ JJs. Left: Optical image of an etched SQUID structure. The overlap between the two NbSe_2_ flakes is highlighted in red in the illustration. Right: Schematic diagram of a SQUID device shown. Reproduced with permission [402]. Copyright (2021), American Chemical Society

The emergence of 2D materials has allowed for the study and application of JJs at a new scale [354, 396], and mechanisms such as the photovoltaic effect, electric field effect, and spin–orbit coupling effect are favorable for the development of new types of JJs. The strong proximity-coupled JJ with extremely clean interfaces was realized in the NbSe_2_-graphene-NbSe_2_ heterostructure [397], where monolayer graphene provides highly transparent short ballistic paths while maintaining strong superconducting phase coherence (Fig. 30a). π JJs are typically fabricated using traditional materials evaporation and sputtering techniques, but the discovery of superconductivity and magnetism in 2D materials provides a new platform for π JJs. VdW stacking enables the realization of π JJs with atomically uniform thickness and sharp interfaces, as shown in Fig. 30b [398]. Nonreciprocal behavior often exists in semiconductor junctions composed of 2D materials, which is conducive to the fabrication of direction-dependent diodes. When an in-plane magnetic field is applied, the Cooper pairs of 2D superconductors can acquire a finite momentum and induce a diode effect, forming a Josephson diode [399]. The discovery of new Josephson phenomena has opened the door to various application studies and promoted the development of future superconducting electronics and quantum devices.

Stacking two layers of 2D materials with a relative twist angle or slight lattice mismatch can result in a larger (quasi) periodic lattice, expanding the potential applications of 2D materials. Magic angle twisted bilayer graphene (MATBG) has been extensively studied as a multifunctional platform that combines metal, superconducting, magnetic, and insulating phases in a single crystal. By locally controlling the electric field in MATBG, it becomes possible to achieve versatile manipulation of quantum devices on a single-material platform. Tunable JJs have been realized on MATBG using multilayer gating techniques, providing independent control of weak links, barriers, and tunneling electrodes in MATBG (Fig. 30e) [400], and observing tunable DC and AC effects. MATBG also forms a superconducting quantum interference device (SQUID) that can locally and globally tune critical currents and inductances [356], with a motion inductance value up to 2 μH [401], far higher than that of 3D devices. Superconducting quantum interference (SQI) technology can be used to reconstruct the supercurrent distribution in twisted BLG (t-BLG) JJs, confirming the topological edge states of t-BLG [89]. JJs and superconducting quantum interference devices have also been implemented in NbSe_2_ thin films with mismatched stacking of two crystal axes (Fig. 30f) [402]. With the continuous development of technology and theory, JJs made from new materials will play an important role in many application areas.

2D vdW JJs also provide new opportunities for implementing various circuit quantum dynamics devices. In quantum circuits, due to the nonlinearity of JJs, Josephson parametric amplifiers (JPAs) can be used to achieve low-noise amplification of weak signals. Graphene JJs with electrically tunable properties can be fabricated into JPAs [403], which have noise performance close to the standard quantum limit. JPAs based on 2D materials are ideal for applications such as quantum amplitude sensing, single qubit readout, single-photon radiation thermometry, and dispersive magnetic sensing. 2D vdW JJs will facilitate the development of basic devices for integrating superconducting quantum circuits in the future.

#### Gate-Controlled Quantum Dots

Quantum dots, as zero-dimensional materials, have ultra-small sizes (< 10 nm), which confine electrons in three-dimensional space and exhibit quantum mechanical effects. Quantum dots possess luminescence, high stability, and tunable band characteristics, showing potential advantages in manufacturing new photonic and nanoelectronic devices with high speed and low power consumption. However, quantum dots suffer from the problem of random growth in size and spatial distribution, which seriously affects their performance and applications. By adding additional tuning knobs such as electric fields, magnetic fields, or mechanical strains, more uniform and controllable quantum dots can be obtained. Quantum dots also face the problem of inhomogeneous line broadening, which leads to distinguishable emission of continuous photons, limiting their performance in applications such as quantum dot single-photon emitters, quantum dot lasers, and quantum dot solar cells. Therefore, it is necessary to control quantum dots through appropriate methods and technologies. The energy level and tunneling barrier of quantum dots can be manipulated by applying an external gate voltage. By adjusting the gate voltage, the Coulomb repulsion can control electrons entering quantum dots one by one. Using appropriate device structures and gating strategies, quantum dots containing a small number of electrons can be realized. The powerful electrical tuning ability of gated quantum dots makes them widely applicable. In the field of quantum computing, gated quantum dots can serve as qubits to store and manipulate quantum information. In the field of quantum communication, gated quantum dots can serve as a light source to achieve single-photon emission and generation of entangled states. In quantum sensing, gated quantum dots can detect physical quantities such as electric fields, magnetic fields, and temperature with high precision.

**Fig. 31:**
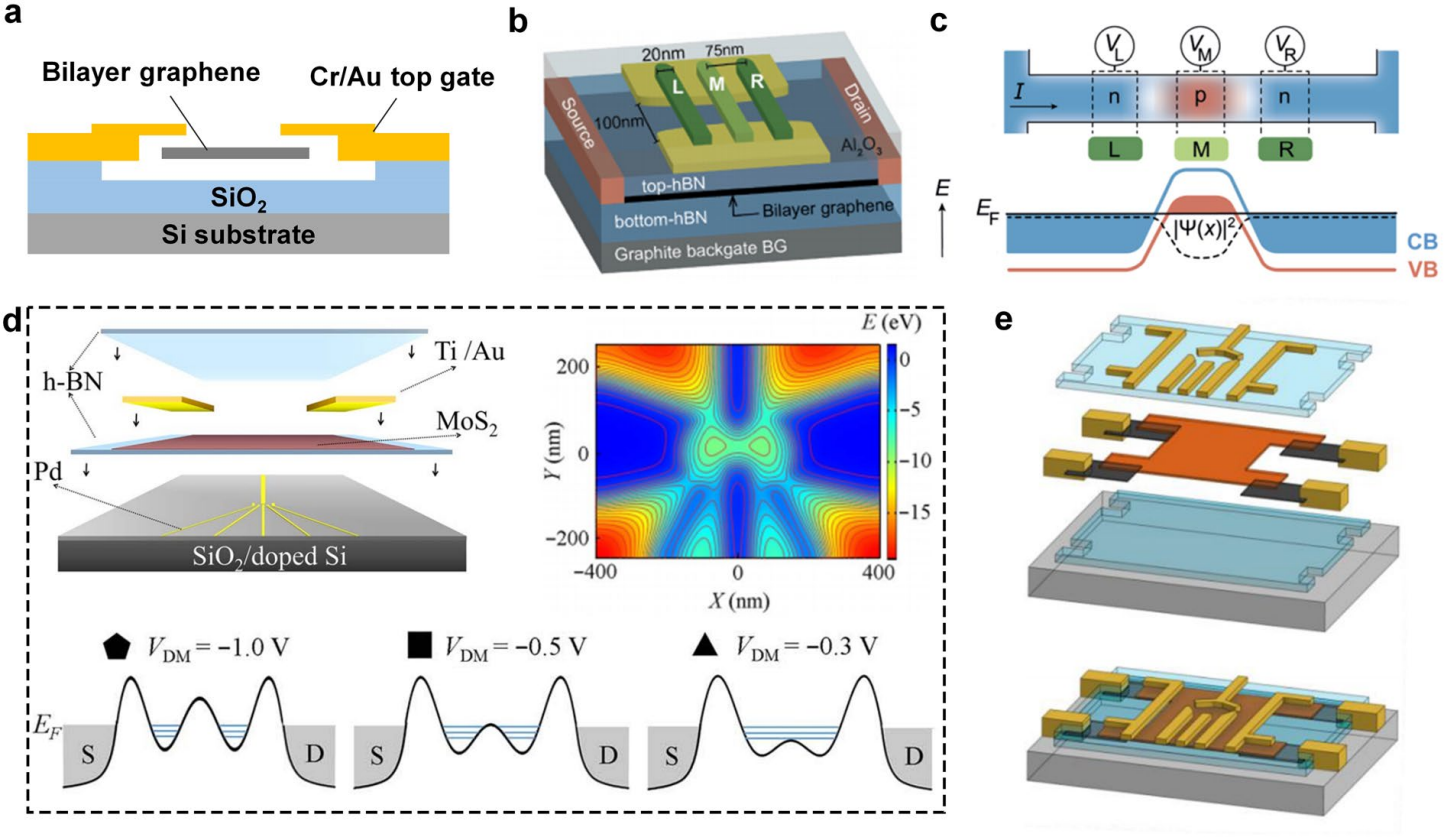
Development of grid-controlled quantum dots in 2D materials. **a** Schematic diagram of gate defining a double-layer graphene quantum dot. Graphene is suspended in electrodes. The electric field and carrier density distributions are controlled by the rear and top grid voltages *V*_b_ and *V*_t_, while transport measurements can be made by applying a biased *V*_sd_ to the electrode [357]. **b** Schematic diagram of gated BLG quantum dots with adjustable polarity. Where, yellow is the split grid and green is the finger grid *L*, *M* and *R*. Make edge contacts (orange) at both ends of the channel [404]. **c** Figure shows the adjustable polarity and band diagram. Reproduced with permission [404]. Copyright (2021), American Chemical Society. **d** h-BN encapsulated grid-controlled MoS_2_ double quantum dots. Above: Schematic diagram of the sample structure on the left, the potential distribution of COMSOL simulated MoS_2_ layer on the right, and the closed contour representing the possible location of the quantum dot. Below: Band structure of MoS_2_ under different gated voltages. This realizes the evolution of a two-quantum-dot system from weak coupling to strong coupling to form a large single quantum-dot. Reproduced with permission [407]. Copyright (2017), American Association for the Advancement of Science. **e** h-BN/InSe/h-BN heterostructure has graphene contacts and multiple top grids. Each layer structure display: 2D InSe (red), graphene (dark gray), h-BN (blue), Cr/Au contacts, top grid (yellow), Si/SiO_2_ substrate (light gray). Reproduced with permission [408]. Copyright (2018), American Chemical Society

Gate-controlled quantum dot structures have been applied to 2D materials and their heterostructures, enabling electric confinement, control, and manipulation of single charge carriers within materials. In terms of gate-modulated nanoscale structures, 2D semiconductors have significant advantages. Graphene, as the first discovered 2D material, directly gates quantum dots on it, which will be affected by edge disorder and charge inhomogeneity caused by ionized impurities in the gate electrolyte. Controlling the external electric field on the suspended BLG can realize the increase of the tunnel barrier for opening the band gap and forming quantum dots with good performance (Fig. 31a) [357]. In gate-controlled few-carrier BLG devices, bipolar operating quantum dots with tunable coupling strength, electronic dot size, and valley g factor are realized. By designing a three-gate tuning [404], the control of the polarity of the carriers in the quantum dots is realized (Fig. 31b, c). This paves the way for the development of graphene in valley qubits. Single-layer TMDCs have a large exciton binding energy, which can prevent charged excitons from dissociating under the influence of an electric field. Due to the technical difficulties of the gate edge contact with TMD, graphene sheets are usually used for bridging and contacting the gate with the metal. It is challenging to create clean and uniformly gapped quantum dots directly in single-layer 2D materials, so two layers of h-BN are usually used to sandwich the 2D material for protection. Through electrostatic gate control in few-layer MoS_2_ encapsulated in h-BN [405], the emission of charged excitons with quasi-one-dimensional spatial span was observed, and single quantum dot preparation was realized. The small spatial variation of the one-dimensional emission line of MoS_2_ can also be used as a sensitive detector for the length and energy scales of residual inhomogeneities in the 2D electron gas. In addition to single quantum dots, gate-controlled double quantum dots have also been formed in 2D materials [406]. The charge stability diagram of MoS_2_, which has multiple gate controls, shows a hexagonal honeycomb pattern, confirming the formation of double quantum dots (Fig. 31d) [407]. By further adjusting the gate voltage, the synthesis from double quantum dots to larger single quantum dots can also be achieved. The monotonous tuning of the point-to-point coupling proves the high tunability of gate-controlled quantum dots based on TMDs. 2D material InSe has relatively small in-plane electron mass and high electron mobility, which allows its electrons to be quantized in one dimension by electrostatic gating. Local top gates are placed at the overlap of InSe/graphene to significantly reduce the contact resistance of InSe (Fig. 31e) [408]. Quantum conductance and Coulomb diamond are observed in this structure, demonstrating the ability to form quantum dots. In gate-controlled BLG devices for few charge carriers, bipolar operation of quantum dots has been achieved, and the coupling strength, size of electronic dots, and valley g-factor are all adjustable. Through the design of three-gate tuning, control of the carrier polarity in quantum dots is realized. This paves the way for the development of graphene in valley quantum bit aspect. The research on 2D gate-controlled quantum dots has promoted the development of spin-valley physics, quantum computing under 2D limits, and various condensed matter physics phenomena, providing new ideas for the development of more applications.

#### Single-Photon Source

Quantum communication technology is rapidly advancing, with researchers conducting extensive studies in areas such as information transmission, storage, and security. Quantum key distribution (QKD) is considered a fundamental aspect of secure quantum communication and requires ideal single-photon sources (SPEs) with antibunching effects, combined with quantum key protocols to achieve key distribution. In long-distance communication, quantum relays are inserted between distant quantum nodes to generate shared keys and compensate for losses due to long channels. Millions of high-performance SPEs are used to produce the required cluster states in quantum relays. Due to the uncloning and unforgeability of single photons, SPEs have become the basic building block of scalable quantum information technology. They are driving the development of future on-chip photon-based quantum processing schemes and are beneficial for the development of a quantum internet.

**Fig. 32:**
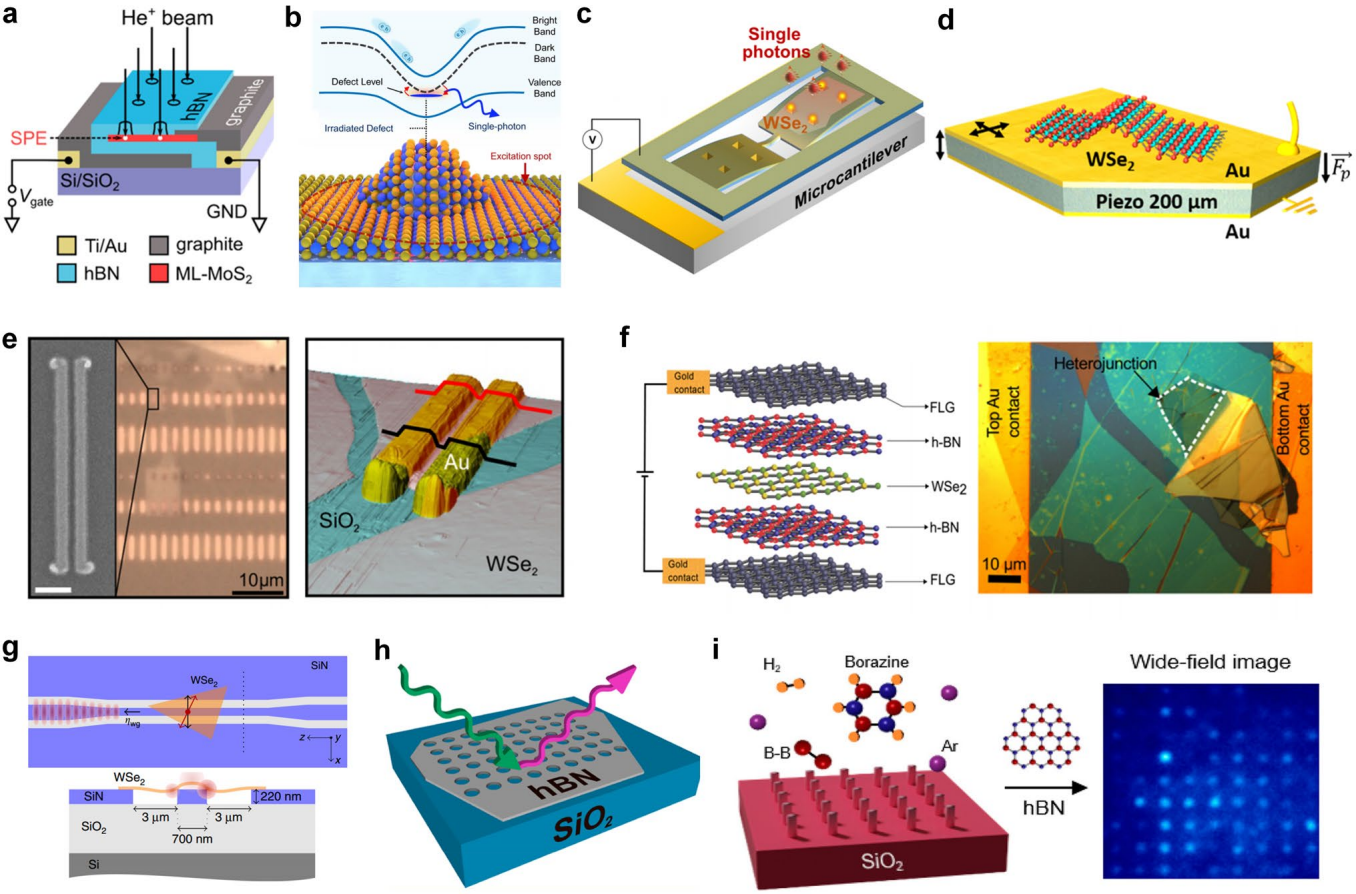
Development of single-photon sources in 2D materials. **a** A single-photon emitter matrix is selectively generated in a MoS_2_ vdW heterogeneous device. Reproduced with permission [411]. Copyright (2021), American Chemical Society. **b** A single-photon emitter with WSe_2_ based on nanoscale strain and electron beam irradiation defects shows a strain map of WSe_2_ on a silica nanocolumn (W atom in blue and Se in yellow). Reproduced with permission [412]. Copyright (2021), Nature Publishing Group. **c** A single layer WSe_2_ single-photon emitter on an electrostatically driven microcantilever with a nanopyramid pattern. Reproduced with permission [413]. Copyright (2019), American Chemical Society. **d** Schematic diagram of a 2D-semiconductor-piezoelectric hybrid driven single-photon emitter with integrated monolayer folds WSe_2_. Reproduced with permission [414]. Copyright (2019), American Chemical Society. **e** Plasma waveguide is coupled with the single-photon emitter to enhance the emission. Reproduced with permission [415]. Copyright (2018), American Chemical Society. **f** Quantum constrained stark effect tuning of a local emitter on a monolayer WSe_2_ edge. Reproduced with permission [417]. Copyright (2017), American Chemical Society. **g** Top view and cross section of the integrated WSe_2_ quantum emitter: WSe_2_ sheet is integrated on a 220 nm thick single-mode SiN waveguide separated from the body SiN by two air grooves. Reproduced with permission [361]. Copyright (2019), Nature Publishing Group. **h** h-BN quantum emission array based on edge effect. Reproduced with permission [418]. Copyright (2019), American Chemical Society. **i** h-BN single-photon emission arrays on nanocolumns. Reproduced with permission [419]. Copyright (2021), American Chemical Society

The absence of dangling bonds in the lattice of 2D materials offers great advantages for SPEs, resulting in higher photon purity. Single-photon emitters embedded in monolayer atoms can avoid total internal reflection and have high light extraction efficiency. Single-photon emission based on 2D materials has been widely studied, but the development of all-electrically operated single-photon devices has been relatively slow. Electrically tunable single-photon emission was first realized in WSe_2_ quantum dot defects, demonstrating that quantum emitters can be operated electrically and providing a prototype for gate-tunable single-photon emitters [409]. Monolayer graphene with excellent conductivity is often used as a gate to regulate single-photon emission in 2D vdW heterogeneous devices. To prevent the single-photon emission layer from being affected by the external environment, it is often sandwiched between two layers of stable h-BN. Single-photon emitters in 2D materials provide a good platform for LEDs [410]. Localized sites in vertically stacked multilayer 2D materials can be electrostatically driven to inject charges into the active layer containing quantum emitters, enabling ultracompact optoelectronic devices-LEDs. To avoid damaging the already prepared single-photon emission defect sites during heterostructure assembly, defect arrays with single-photon emission functionality can be selectively generated after assembling into gate-tunable heterostructures, as shown in Fig. 32a [411]. This approach has been realized in a heterojunction field-effect device with graphene as the gate, h-BN as the dielectric, and a monolayer of MoS_2_ as the quantum emission layer [411], which promotes the further development of gate-addressable and gate-switchable single-photon emitters.

The long-term goal of quantum emitters is to achieve control over the wavelength and position of single-photon emission at the device level. The exciton characteristics of 2D materials are sensitive to layer number and lattice strain and can be modulated by manufacturing processes and external strain (Fig. 32b) [412] to control material electrical and optical properties. Strain platforms can manipulate the linewidth, polarization, exciton binding energy, and other parameters of single-photon emission to achieve single-photon emission with different performance metrics. Electrostatically driven microcantilevers (Fig. 32c) [413], piezoelectric actuators (Fig. 32d) [414], and plasmonic waveguide surfaces (Fig. 32e) [415] can all provide local strain platforms for 2D single-photon emitters. Since localized excitons are sensitive to the nanoscale proximal dielectric environment, SPEs can also be coupled to plasmas to improve performance, enhancing the potential for nanoscale photonics and quantum devices. The Stark effect can also modulate exciton characteristics such as bandgap and exciton binding energy, and can tune single-photon performance through the application of an external electric field. A linear Stark shift of up to 5.4 nm per GV/m has been achieved by applying an out-of-plane electric field to atomic defects in h-BN in an h-BN/graphene heterostructure [416]. The quantum-confined Stark effect (QCSE) exists in quantum wells, tilting the bandgap under the influence of a built-in electric field and generating a dipole opposite in direction to the electric field, thus manipulating the exciton wave function of single-photon emitters. Specific vdW heterostructures can form quantum wells with quantum dot defects in 2D materials, and single-photon emission can be modulated by applying a vertical electric field (Fig. 32f) [417]. The connection of a single-molecule layer to an integrated dielectric cavity has been reported (Fig. 32g) [361], and its combination with wafer-level CVD growth of 2D materials provides new opportunities for quantum photonics chips.

With the increase of information, the single-photon source also develops toward an integrated array, which is conducive to integration into quantum chip. Local strain or defect injection is conducive to forming a quantum emission array in 2D materials. Defect-induced quantum emission is usually present at the edge of the 2D material. Researchers have observed a bright local photoluminescence array that matches the geometric shape of a patterned hole by focusing an ion beam (FIB) to embed a series of patterned holes into h-BN, as shown in Fig. 32h [418]. Dielectric nanorod arrays are also commonly employed to provide a strain-engineered single-photon emission array, as shown in Fig. 32i [419]. Single-photon emission arrays have been achieved by combining nanorods with WS_2_ [360], WSe_2_ [420], and h-BN [418, 419]. When a single-photon source is coupled with a nanometer-sized antenna or a dielectric cavity, an enhancement in quantum emission efficiency has been observed. A high-efficiency quantum emission array has been realized by using an array of metal with quantum emission enhancement function, coupled with 2D materials [421].

The deterministic generation and control of multiple quantum emitters serve as the foundation for future scalable and integrated quantum photonics systems. Active control systems with wavelength feedback can stabilize the single-photon emission frequency, enabling the integration of single-layer photon emitters with optical cavities, resonators, and detectors, providing possibilities for on-chip integrated quantum photonics circuits. The control of single photons using electric fields, magnetic fields, and strain is an important device of future quantum photonics systems. Combining single photons with linear optics can also realize more complex quantum applications such as quantum logic gates and quantum interference. The entanglement of photon pairs is beneficial for the development of scalable quantum technologies. In quantum communication, key generation still heavily relies on attenuated lasers, which has the issue of photon divergence. The development of on-demand single-photon states in 2D materials offers a new approach for QKD, and simulation of the BB84 protocol has been achieved in single-layer WSe_2_ quantum sources [362], accelerating the application of SPEs in quantum communication.

#### Solid-state Quantum Sensors

Quantum sensors rely on the effects of quantized energy levels, quantum coherence, or quantum entanglement to achieve higher sensitivity and measurement accuracy. Quantum sensing technology can be used to create super-sensitive magnetometers, ultra-precise compasses, quantum lithography, atomic clocks, etc., providing exciting prospects for navigation, imaging technology, and natural disaster prediction.

Solid-state spin defects with discrete and tunable energy levels can be used to measure disturbances in the environment and are a critical device of quantum sensing technology [364]. Currently, the spin system for quantum sensing is mainly focused on nitrogen-vacancy (NV) centers in diamond, where three-dimensional materials provide natural protection for the embedded spin defects. However, preparing surface spin defects in three-dimensional bulk materials is challenging, limiting their precise contact with external object and hindering on-chip integration of quantum sensors with complex equipment. 2D materials can be easily exfoliated into several layers, creating surface spin defects that are comparable in size to the atoms of the object being measured. The narrow spin transition linewidths of spin-active defects in 2D materials greatly enhance the generality and sensitivity of quantum sensing technology. However, a significant amount of electrical noise and magnetic field noise in the environment can also affect quantum defects in monolayer materials, posing a long-term challenge for the development of efficient sensors. The development of monolayer-limited quantum sensors still has a long way to go.

**Fig. 33:**
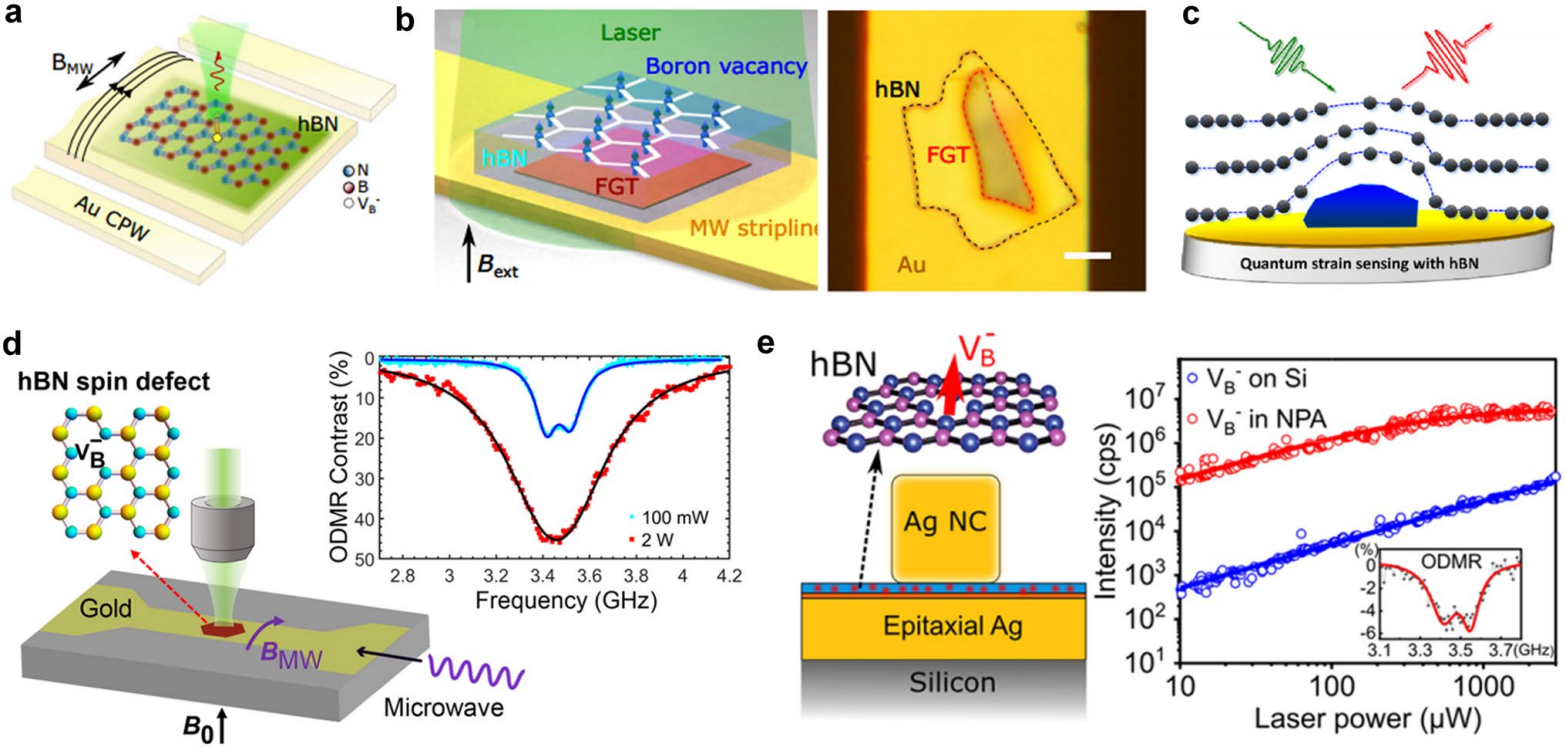
Development of quantum sensors in 2D materials. **a** Schematic diagram of spin activity transition detection device for VB^−^ defects in h-BN. The spin states are manipulated by laser and microwave fields. The fluorescence spectra were collected and the spin states were read from the ODMR signal. Reproduced with permission [422]. Copyright (2022), Nature Publishing Group. **b** Nanoscale quantum imaging of low-dimensional Fe_3_GeTe_2_ (FGT) with ferromagnetism by spin defects in h-BN. Reproduced with permission [423]. Copyright (2022), Nature Publishing Group. **c** Strain quantum sensing of VB^−^ defects in h-BN. Reproduced with permission [425]. Copyright (2022), American Chemical Society. **d** Photoluminescence enhancement of h-BN spin defects by a gold film microwave waveguide. Its ODMR contrast is up to 46%. Reproduced with permission [427]. Copyright (2021), American Chemical Society. **e** High resolution magnetic field sensor based on low-loss nano-patch antenna (NPAs) to enhance VB^−^ defect photon emission. An overall strength increase of up to 250 times was observed. Reproduced with permission [428]. Copyright (2022), American Chemical Society

Recently, atomic defects in h-BN have received increasing attention as alternative materials for studying light-matter interactions, nanophotonics, and nanoscale sensing. In negatively charged boron vacancies of h-BN, an electron spin triplet state has been observed, in which the ground state is split by zero-field, and these spin states can be manipulated by laser and microwave fields (Fig. 33a) [422]. VB^−^ defects are considered promising quantum sensors due to their excellent quantum coherence and single spin addressability, and can be used for detecting temperature [364], pressure, magnetic fields (Fig. 33b) [364, 423, 424], and strain (Fig. 33c) [425]. There are many ways to create VB^−^ defects, including ion implantation, electron irradiation, neutron irradiation, and femtosecond laser writing. Coupling h-BN defects with nanophotonic devices with waveguides can improve the signal-to-noise ratio of the sensor [426]. However, the contrast of ODMR and photoluminescence from h-BN spin defects is relatively low, which limits its sensitivity. Therefore, it is necessary to increase the overall quantum efficiency by accelerating the photon emission rate while maintaining the spin properties. Gold film microwave waveguides are often used to enhance the luminescence intensity of spin defects and ODMR signals in h-BN [427], as gold surfaces can enhance plasmon emission and control spin (Fig. 33d). Through the effect of gold film waveguides, the ODMR contrast of h-BN nanosheets at room temperature reached 46%, and the photoluminescence was enhanced by 17 times. The plasmon enhancement largely depends on the type of plasmonic material. Silver thin films are easier to make into low-loss nano-patch antennas (NPA) [428], which greatly improves the efficiency of plasmonic cavities and produces higher Purcell enhancement (Fig. 33e). Using silver film-based NPA, the overall emission intensity reached up to 250 times, which is conducive to isolating single VB^−^ defects and further extending the application of VB^−^ in the field of quantum information. VB^−^ spin defects can serve as local probes and, through integration with low-dimensional materials, enable nanoscale quantum sensing and imaging of the material’s magnetism [423, 429]. In h-BN with point defects, a multifunctional quantum microscope was realized [429]. Time-resolved imaging near the Curie temperature of vdW ferromagnetism was successfully demonstrated, as well as temperature and magnetic imaging, and the charge current and Joule heating in operation of graphene devices were mapped. The integration of h-BN with multifunctional heterostructures opens up new prospects for in situ quantum sensing.

Quantum sensing enables the detection of various physical fields, and the integration of multifunctional heterostructures will drive the development of a series of devices, pushing quantum sensing toward broader application areas. At the same time, quantum sensing will also benefit from the progress in quantum computing, particularly in the fundamental understanding of qubits. Multiple sensors can cooperate by sharing quantum entangled states, improving the signal-to-noise ratio and measurement accuracy. This cooperative technique can be used in distributed quantum sensors, further expanding their application scope to the field of precision measurements.

### Quantum Circuit

A quantum circuit is used to describe the interaction between quantum gates and qubits in quantum algorithms. Depending on the physical carrier used to implement the qubits, quantum circuits can be divided into quantum electronic circuits and quantum optical circuits. Among them, quantum electronic circuits have been developed earlier. Quantum electronic circuits are based on physical quantities such as current and charge and mainly include superconducting quantum circuits and semiconductor quantum circuits. Superconducting quantum circuits have made significant progress [430] in implementing notable quantum algorithms such as the Grover search algorithm and the Shor algorithm, as well as in simulating complex processes like chemical reactions and high-energy physics. It is worth noting that while superconducting quantum circuits have successfully implemented important quantum algorithms and simulations, the current system scale and stability still pose challenges. Semiconductor quantum circuits utilize modern advanced semiconductor nanofabrication processes to prepare, manipulate, and read qubits purely through electrical control, enabling easy realization of large-scale integration. The development of quantum electronic circuits based on 2D materials mainly focuses on superconducting quantum circuits, which can realize the generation, manipulation, and measurement of superconducting qubits. They are usually composed of superconducting qubits, microwave resonant cavities, and control circuits. Quantum optical circuits can realize the generation, manipulation, and detection of non-classical light. They are usually composed of single-photon sources, photonic crystal waveguides, micro-ring resonators, and other components, and can be used to achieve long-distance quantum communication and distributed quantum computing. Quantum photonic circuits leverage the intrinsic properties of photons to densely integrate diverse optical components on a chip in a compact manner. Quantum integrated photonics holds several advantages, including small size, high integration density, and strong resistance to interference. In three-dimensional materials, quantum optical chips have been developed that integrate multiple devices capable of quantum information processing. The integration of single-photon sources based on 2D materials and optical waveguides will facilitate the further development of quantum optical circuits that combine 2D materials with photonic ICs.

**Fig. 34:**
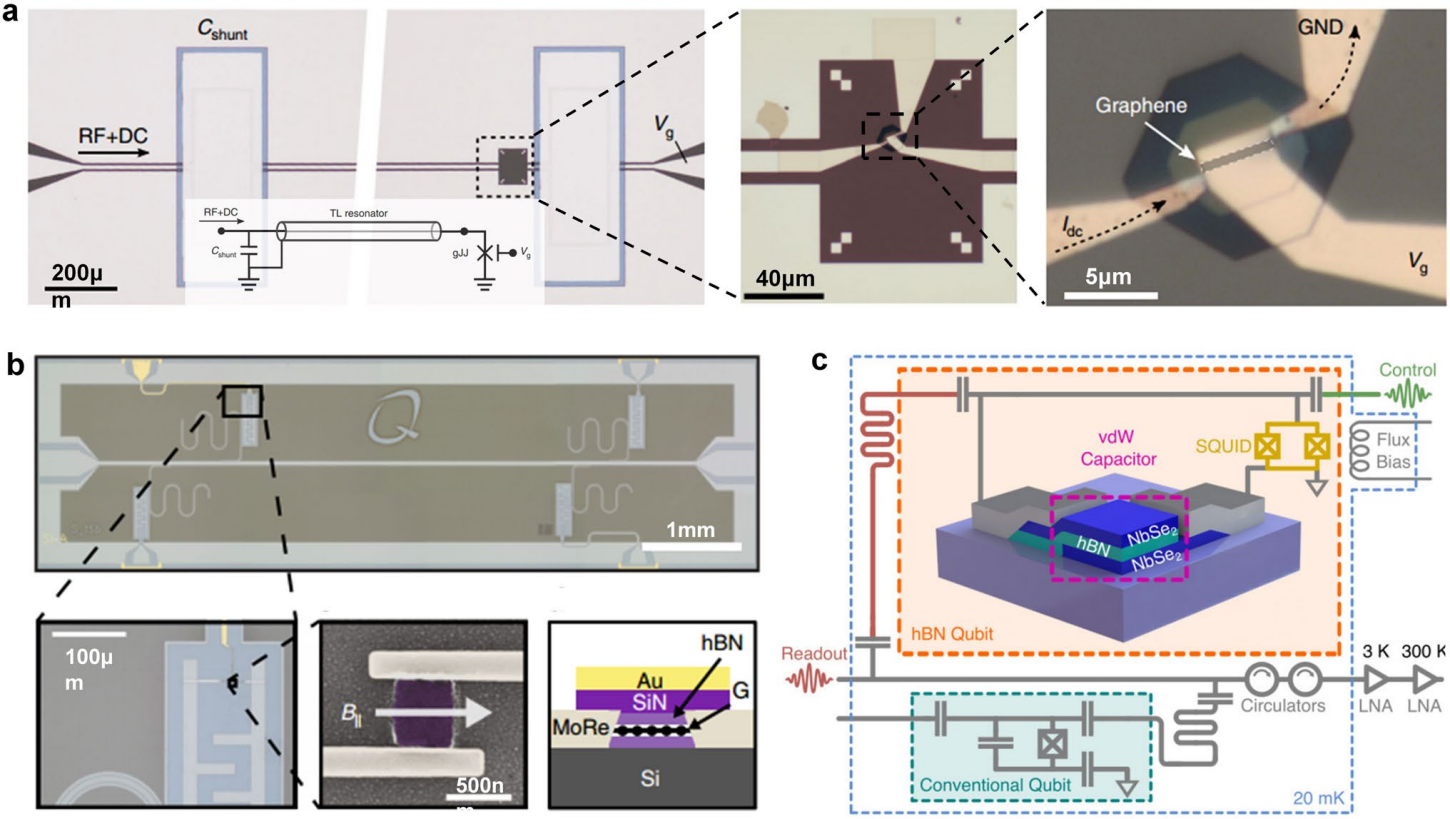
**a** A superconducting quantum circuit based on a conventional aluminum-based JJ and a parallel plate capacitor combination consisting of superconducting niobium selenide and insulated h-BN crystal layers. The transmitter capacitor is coupled to the read resonator (red) and the drive line (green). Reproduced with permission [432]. Copyright (2018), Nature Publishing Group. **b** A hybrid superconducting circuit based on S-G-S JJ. Reproduced with permission [431]. Copyright (2018), Nature Publishing Group. **c** Monolayers of graphene Josephson are incorporated into microwave-frequency superconducting circuits to create graphene-based transporters. Reproduced with permission [433]. Copyright (2021), American Chemical Society

**Fig. 35:**
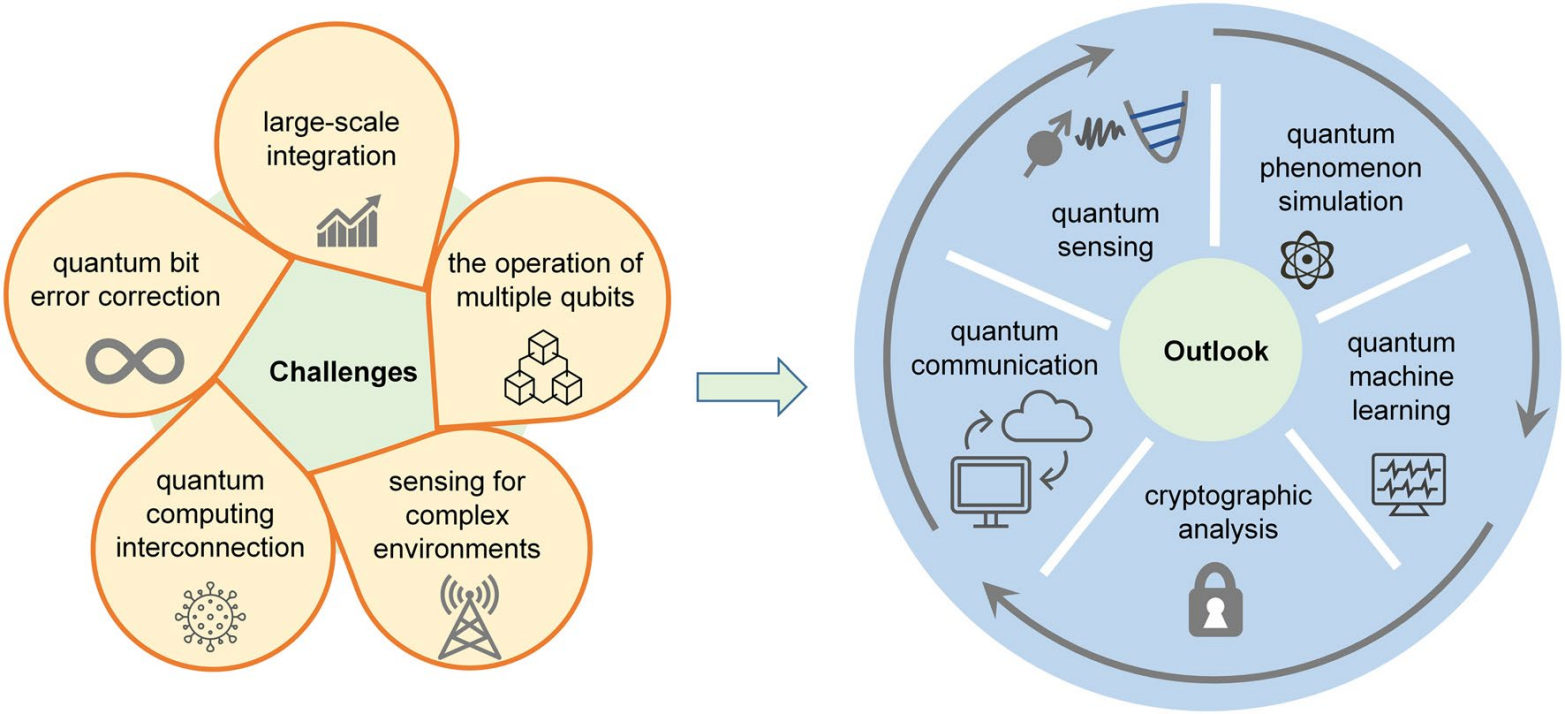
Challenges (large-scale integration, the operation of multiple qubits, quantum bit error correction, quantum computing interconnection, sensing for complex environments) and outlook (quantum phenomenon simulation, quantum machine learning, quantum communication, quantum sensing, cryptographic analysis) of 2D materials in quantum technology

By applying microwave pulse signals to the superconducting circuit, the interactions between qubits can be precisely controlled, enabling quantum gate operations. By coupling multiple superconducting qubits, it becomes possible to achieve complex quantum computations and other functionalities. Meanwhile, superconducting circuits have low resistance and inductance, which effectively reduces energy losses and noise generation, allowing for long-lived qubits. The implementation of a series of quantum algorithms and quantum simulations holds significant importance for further advancements in quantum computing. High-intensity, high-purity magnetic fields are commonly used to manipulate quantum states, and quantum circuits that transport quantum states must be able to operate stably in high magnetic field environments. When graphene JJs are used in quantum circuits, the single atomic thickness of graphene makes the device insensitive to magnetic fields (Fig. 34b) [431], demonstrating the feasibility of using graphene in quantum circuits. Superconducting microwave circuits are crucial for implementing quantum gate operations and signal readout, and graphene JJs capable of achieving ballistic transport have been used in these circuits to observe their microwave properties (Fig. 34a) [432], indicating that graphene JJs can provide a feasible platform for coherent quantum circuits. Superconducting circuits include capacitors and inductors. Conventional capacitors are typically composed of two metal plates sandwiching an insulating layer, while quantum bit capacitors require an additional vacuum insulation layer. This is to protect the high precision and stability of quantum computation, reduce external environmental interference, and avoid loss of dielectric materials. This common quantum bit capacitor requires a large size space, resulting in a large volume of a quantum bit. The emergence of 2D materials provides new ideas for quantum bit capacitors. H-BN can be used as an insulating layer for capacitors, and it can maintain its defect-free state at ultralow temperatures and high-frequency electric fields. Using vdW materials, it is possible to reduce the quantum bit area without sacrificing capacitance and quantum coherence. Abhinandan Antony et al. combined parallel plate capacitors (PPCs) composed of superconducting NbSe_2_ and insulating h-BN crystal layers with traditional aluminum-based JJs, reducing the single quantum bit area to one-thousandth of that of traditional quantum chips [433], as shown in Figs. 34c, 35. The vdW transmon T_1_ relaxation time is 1.06 μs. Another group of researchers used the same structure of PPCs to achieve a transmon quantum bit with a coherence time of up to 25 μs and confirmed that h-BN is a low-loss dielectric [434]. H-BN can be used to construct high-coherence quantum circuits and helps reduce crosstalk between qubits. Hybrid superconducting circuits can obtain their coherent properties by interacting with artificial atoms (qubits) or microwave photons. It is highly necessary to construct more quantum bit states based on this method and investigate the issue of crosstalk between qubits. This will bring about a new wave of research. Hybrid superconducting circuits based on ballistic superconductor-graphene-superconductor (S-G-S) structures have demonstrated quantum coherent control in circuit quantum electrodynamics (cQED) technology and demonstrated transmon qubits [358]. Superconducting qubits based on graphene have high stability and long coherence time characteristics, are easy to implement large-scale quantum computing, and are of great significance for topological quantum computing architecture.

Quantum circuits based on 2D materials have advantages such as high scalability, low power consumption, and low noise, making them widely applicable in the fields of quantum information processing, sensors, and quantum communication. However, quantum circuits need ultrahigh integration of qubits to realize quantum computers. Currently, quantum circuits based on 2D materials are only at the level of a few qubits. And the electrons in 2D materials are susceptible to strong impurity scattering, which makes the quantum bit unstable. Additionally, the weak adsorption capability of 2D materials makes it difficult to match them with metal electrodes. As mentioned above, there are still significant challenges in integrating quantum circuits based on 2D materials, and continuous exploration is needed. Quantum circuits based on 2D materials will become an important device of future quantum technologies.

2D materials exhibit special quantum phenomena, giving them inherent advantages in quantum technology, providing great potential for the development of integrated quantum technology. Manufacturing technologies such as defect control, epitaxial growth, and mechanical exfoliation may cause instability in the properties of 2D materials, which still need to be explored for large-scale high-quality 2D quantum materials [435]. In terms of qubits, they are susceptible to environmental noise, which can lead to bit errors, requiring continuous error correction to ensure the accuracy of information. As the integration of qubits increases, more signal lines are needed for precise control and reading of each quantum bit, which will face wiring problems. Vertical wiring can solve hundreds of qubits, but with the increase in the number of bits, better wiring methods will be needed. The design of stable, highly integrated quantum bit circuits and the realization of coordinated quantum computing both face huge challenges. 2D material surfaces are smooth and difficult to make ohmic contact with electrodes. They usually use graphene or metal electrodes in direct contact with 2D materials, which brings high contact resistance and limits their application. In the realm of quantum communication, the compatibility and matching between 2D materials and optical devices pose significant challenges. Furthermore, due to the sensitivity of 2D materials to the environment and their susceptibility to environmental noise and impurities, their sensing capabilities in complex environments face challenges. Future research and technological advancements are expected to address these issues, further driving the development of 2D materials in quantum privacy protection, quantum simulation, quantum machine learning, quantum cryptography, quantum sensing, quantum optimization, and other fields, with profound impacts on scientific research, engineering technology, and national security.

## Lab-to-Fab Transition: Outlooks

Since the discovery of graphene in 2004, the field of 2D materials has undergone nearly two decades of development, with both the depth and breadth of research in this area continuously expanding. Simultaneously, the process of commercializing 2D materials has been steadily advancing. Currently, commercialization efforts for 2D materials are primarily concentrated in the following areas: wafer-scale production of 2D materials, with companies like 6Carbon Technology in China emerging as one of the world’s largest manufacturers, capable of mass-producing high-quality 2D films, including TMDCs, graphene, h-BN, and source crystals. Additionally, Hefei Microcrystalline Company has been producing graphene-based products. However, the supply of single-layer 2D materials remains primarily oriented toward research institutions and universities, serving fundamental research and integrated circuit process technology development. Regarding graphene-based products, some commercialization has been achieved as graphene is utilized as an auxiliary material, primarily aimed at enhancing performance. For instance, graphene’s exceptional thermal conductivity has led to its application as a heat-dissipating material in electronic products, such as smartphones and tablets introduced by Huawei. Moreover, graphene-based coatings and suspensions developed by Hefei Microcrystalline Company have found applications in corrosion resistance and antimicrobial fields.

When it comes to semiconductor industry, there is substantial attention directed toward 2D materials. Further downscaling in Si-based technology has become increasingly challenging due to both process limitations and fundamental device constraints. Several prominent semiconductor industry players, including IMEC, TSMC, and Intel, have actively explored the utilization of 2D materials to extend Moore’s law [436–438]. On the one hand, fundamental research efforts at the device level involving 2D materials, such as material growth, optimization of contact resistance, dielectric integration, and device miniaturization, aim to substantiate the optimization potential and performance advantages offered by 2D materials. Intel demonstrated and characterized MOCVD of 2D materials directly on a 300-mm Si platform [437]. TSMC reported the epitaxial growth of single-crystal h-BN monolayers on a Cu film across a two-inch sapphire wafer [438]. Furthermore, Intel explored 2D channel FET scaling down to source-to-drain contact spacing of 25nm, comparable to state-of-the-art Si technology [439].

On the other hand, continuous endeavors focus on verifying the scalability of 2D materials manufacturing and their compatibility with silicon-based production lines. Intel presented multiple applications of 2D materials in the BEOL [440]. We also noticed that IMEC reported on integration challenges and optimized process uniformity for a single-device yield higher than 90% across full 300mm wafers [441]. The efforts of these companies make the devices suitable for hybrid 2D integration in the BEOL.

It is evident that the current state of industrialization for 2D materials is less than ideal, with a predominant focus on low-value-added goods and scientific research. Furthermore, their presence in the semiconductor industry remains relatively limited. In our opinion, for 2D materials to find application in the semiconductor industry, they shall meet the following requirements: (1) Device Performance: The advantages of using 2D materials in transistors, especially in scaling down, have been reported. However, for chip applications, it is essential not only to harness the excellent electronic properties of these materials but also to ensure the stability of performance and the coordinated optimization of various parameters. This entails control over material mobility, carrier concentration, and defect management. Currently, much of the research on 2D materials has focused on optimizing individual device performance, while studies on doping processes and stability remain relatively lacking. (2) Process Compatibility: considering the performance and fabrication capabilities of 2D materials fall short of replacing silicon, they are more likely to be integrated heterogeneously with silicon. Therefore, it is crucial that 2D materials and their processing methods are compatible with existing silicon-based fabrication processes. The preparation of high-quality 2D materials mainly relies on high-temperature processes and specific precursor materials. While progress has been made in the low-temperature synthesis processes, the quality of prepared 2D materials still requires further optimization. Compatibility with silicon-based production and its impact on silicon-based manufacturing processes require further evaluation. (3) Scale Manufacturing Capability: achieving scalability and mass production relies on device yield and uniformity, particularly in the context of chip manufacturing. Consistency in device performance and the matching of module-level circuits are critical factors limiting circuit scalability.

Industrialization differs from laboratory-scale scientific research, requiring a more comprehensive and streamlined approach. In addition to laboratory-level research on 2D materials, there is a need to further enrich and develop the technological expertise related to industrialization. We believe that efforts should be made in the following aspects:

1. Cost-effective synthesis technologies: Substantial challenges still exist in controlling key quality parameters such as thickness, uniformity, continuity, defects, grain size, phase, and polarity that directly impact the device yield and performance. Moreover, due to the variations in equipment conditions in different laboratories, there is no unified material fabrication strategy or quality evaluation system now. Achieving large-scale production in the future will necessitate the establishment of comprehensive assessment standards. Furthermore, traditional ion implantation methods are not feasible due to the atomically thin nature of 2D materials. And the tuning of carrier polarity remains a major challenge. Furthermore, the insertion of 2D materials into high volume manufacturing may require the development of new cost-effective fabrication technologies, despite their proof of concept and potential solution.
2. Factory integration: Ensure that the manufacturing infrastructure of the 2D material chip production line incorporates essential or specialized components to fulfill the demands of automated and intelligent processing of 2D material chips. Due to the extremely high growth temperature of 2D materials, it is necessary to add a unique process area for 2D materials in traditional CMOS process lines, such as large size CVD growth systems, full-automatic transfer equipment, and so on.
3. Circuit Design: 2D devices exhibit distinct material properties and performance characteristics compared to silicon-based devices. Therefore, the design of 2D circuits cannot simply adopt the schemes and methods used for silicon-based circuits. To achieve fully functional 2D circuits in the future, further development of a comprehensive set of design processes, methodologies, and accompanying software specific to 2D materials is necessary. Besides, whenever a new semiconductor process is required, the initial step involves developing a PDK. The PDK encompasses comprehensive documentation of the fabrication process in the foundry’s language, playing a pivotal role in ensuring successful chip manufacturing. Additionally, there may be challenges related to thermal load and inter-chip crosstalk when implementing circuits. Redundancy design methods can be considered during circuit design to enhance chip yield.
4. Package and test: The susceptibility of 2D materials, such as graphene, to pollution and oxidation in ambient air environments leads to performance degradation, necessitating the implementation of packaging and other protective measures. Last but not least, the evaluation and testing of wafers, which span the entire process of wafer production, constitute a pivotal stage in the commercialization of 2D materials. The microstructure characterization, composition and phase analysis, failure behavior analysis, and physical and chemical performance testing of 2D materials require further improvement.
5. Metrology and characterization: Real-time observations during the growth of 2D materials and device fabrication processes are essential for understanding the material growth mechanisms and ensuring quality control [67]. Given the atomically layered nature of 2D materials, the development of non-destructive characterization techniques, such as optical monitoring, is of paramount importance. On the one hand, it is crucial to leverage existing traditional characterization techniques while adapting them to the unique characteristics of 2D materials. On the other hand, there is a need to develop more targeted or specialized characterization methods to provide precise information about material structure and morphology. Furthermore, there is a requirement to establish comprehensive material quality and monitoring assessment systems to enable in situ monitoring of the structural quality and overall properties of these materials, which is indispensable in the fabrication workflow.

Considering the current state of 2D materials development and the concerted efforts within the industry, we identify three potential pathways for the future of 2D materials:

Firstly, the development of an all-2D system, particularly in the realm of analog–digital circuitry, capitalizes on the advantages of 2D materials in terms of size reduction. While significant progress has been made in device-level research, the primary challenge lies in seamlessly integrating 2D materials into advanced node technologies and establishing a comprehensive ecosystem for 2D chip fabrication. Compared to the mature silicon-based technologies that have been developed for decades, this pathway offers substantial room for enhancement and optimization.

Secondly, the development of heterogeneous integration chip technologies involves the creation of silicon-based—2D material-based stacked chips or partitioned integration, such as chiplet technology. This direction leverages existing silicon-based ecosystem technologies to further develop silicon-compatible integration processes for 2D materials. It represents one of the most promising technological pathways currently available.

Thirdly, in mainstream applications, 2D materials are less likely to completely replace mature bulk materials such as Si and GaN. Instead, they are more suitable for use in more selective application domains with relatively lower material quality requirements, while harnessing the unique advantages of 2D materials, such as low operating current and leakage current, as well as the multifunctionality of 2D materials. Examples of such application domains include low-power flexible sensors and neuromorphic computing. Utilizing existing cost-effective manufacturing processes, there is a promising potential to expedite the industrialization of 2D materials in the near future.

In summary, the advancement of 2D technologies should progress within the foundation of the well-established silicon-based processing framework. The commercial applications of 2D materials are still in an early stage. The growth of WSSC materials remains a roadblock hindering their industrialization. Furthermore, there is a need for further development and improvement in supporting manufacturing equipment, design software, material characterization, and evaluation systems, as well as research into 2D-compatible doping or encapsulation processes. Additionally, it is important to broaden the research focus beyond the development of advanced logic chips. As discussed in this section, heterogeneous integration chips and specific application directions should receive significant attention as they offer more potential and achievable development paths. Moreover, it is crucial to emphasize that the commercialization of 2D materials should leverage the exploratory advantages of academic sectors and technical foundations of industrial sectors. Close collaboration and coordination between these two sectors can facilitate smoother development for more seamless progress.
